# High hydrostatic pressure specifically affects molecular dynamics and shape of low-density lipoprotein particles

**DOI:** 10.1038/srep46034

**Published:** 2017-04-06

**Authors:** M. Golub, B. Lehofer, N. Martinez, J. Ollivier, J. Kohlbrecher, R. Prassl, J. Peters

**Affiliations:** 1 Univ. Grenoble Alpes, IBS, Grenoble, F-38000, France; 2 Institut Laue Langevin, Grenoble, F-38000, France; 3 Institute of Biophysics, Medical University of Graz, Graz, A-8010, Austria; 4 Paul-Scherrer-Institut, Villigen, CH-5232, Switzerland; 5 Univ. Grenoble Alpes, LiPhy, Grenoble, F-38044, France

## Abstract

Lipid composition of human low-density lipoprotein (LDL) and its physicochemical characteristics are relevant for proper functioning of lipid transport in the blood circulation. To explore dynamical and structural features of LDL particles with either a normal or a triglyceride-rich lipid composition we combined coherent and incoherent neutron scattering methods. The investigations were carried out under high hydrostatic pressure (HHP), which is a versatile tool to study the physicochemical behavior of biomolecules in solution at a molecular level. Within both neutron techniques we applied HHP to probe the shape and degree of freedom of the possible motions (within the time windows of 15 and 100 ps) and consequently the flexibility of LDL particles. We found that HHP does not change the types of motion in LDL, but influences the portion of motions participating. Contrary to our assumption that lipoprotein particles, like membranes, are highly sensitive to pressure we determined that LDL copes surprisingly well with high pressure conditions, although the lipid composition, particularly the triglyceride content of the particles, impacts the molecular dynamics and shape arrangement of LDL under pressure.

Human plasma lipoproteins are macromolecular assemblies of lipids and proteins, which function as lipid transporter in blood circulation. Lipoproteins have shown a distinct dynamical behavior as function of temperature^1^, which is very similar to membranes, and we assume that lipoprotein particles, like membranes, are highly sensitive to pressure as well. Thus, the conception of a dynamic landscape for lipoprotein species under high hydrostatic pressure (HHP), similar to natural membranes, is very appealing, but has never been evidenced before. Here we report for the first time on the application of HHP on human plasma low-density lipoprotein (LDL), whose main function is the transportation of cholesterol from the liver to peripheral tissue. We applied HHP to compare the dynamical behaviour of LDL particles with a healthy normolipidemic composition (N-LDL) to a triglyceride-rich form of LDL (TG-LDL), which mimics pathologically modified LDL. The chemical compositions of LDL particles are well described in the literature^2^,^3^,^4^,^5^. Each particle (Fig. 1) consists of a complex combination of lipids and a single copy of a protein, termed apolipoprotein B100 (apoB100). LDL has a hydrophobic core and an amphiphilic shell. The core basically harbors cholesteryl esters, triglycerides and parts of the unesterified cholesterol. The shell consists of a phospholipid monolayer, unesterified cholesterol and the amphipathic apoB100 molecule wrapped around the surface^6^. It is well known that lipoproteins play a key role in atherosclerosis, which is a dynamic and progressive inflammatory disease that promotes lipid deposition in the arteries^7^,^8^. Moreover, the lipid composition of the LDL core becomes modified in case of certain diseases such as *hyperlipidemia*, where LDL is enriched with cholesterol or triglycerides^8^,^9^. Such variations in the lipid core composition may influence the overall particle size and the molecular arrangement of surface components^10^, however, little is known how the particles compensate for changes in the surface area and surface pressure depending on the chemical composition of the core lipids^11^.

Pressure is a thermodynamic variable which, besides temperature, enables to investigate physical parameters of macromolecules, and to explore dynamical and structural properties of supramolecular assemblies. The choice to use HHP instead of temperature is based on the following considerations: about 30 years ago, researchers found that not only the structure of a protein is mandatory for its functioning, but also its dynamics^12^,^13^. However, temperature variations influence the thermal energy and the volume of a system through the thermal expansion factor simultaneously, which makes it difficult to separate these two effects. On the contrary, pressure affects only the volume of the investigated system and therefore induces controlled changes of intermolecular distances^14^. Pressure gives access to the partial molar volume, a parameter intimately related to structure and dynamics of biomolecules. As biological systems might be in different conformational sub-states according to their free energy^15^, this latter quantity determines the degree of freedom of the possible motions and consequently the flexibility of the sample.

To probe the molecular dynamics of LDL in the ps-ns time range we used elastic incoherent neutron scattering (EINS) and quasi-elastic neutron scattering (QENS). As hydrogens are typically homogeneously distributed in biological systems, their motions provide averaged information about molecular movements at atomic scale, which can be determined for different pressure values. The hydrogen contribution is dominating the spectra, since the incoherent neutron cross section (NCS) of hydrogen is much larger than the NCS of any other atom present in biological objects^16^. In addition to the dynamical investigations, we used small-angle neutron scattering (SANS), which is one of the most suitable techniques to answer structural questions of biomolecules in solution, since it furnishes information on shape, domain organization and molecular interactions^17^,^18^,^19^,^20^. The results of these techniques have been combined to gain a general picture of LDL dynamics and structure under high hydrostatic pressure.

All experiments were carried out at body temperature (310 K), which is a temperature above the phase transition temperature of the lipids, and covered the pressure range from 20 to 3000 bar to determine molecular motions and structural remodelling upon HHP. The maximum pressure was far beyond pressure values found on Earth, but the idea was to highlight effects which are hardly distinguishable at lower pressure. QENS data allowed to assign motions to different dynamical populations, associated to specific molecules or molecular groups within LDL. To perform the studies, we took advantage of the HHP equipment developed specifically for biological samples in solution at two different neutron scattering facilities. Our data suggest that an interplay between structural flexibility of the particles and intrinsic molecular dynamics of lipids and proteins partially compensates for variations in the chemical composition of the particles, even at extreme external stress conditions.

## Results

### TG-LDL dynamics is sensitive to HHP application in contrast to N-LDL dynamics

**Figure 2:**
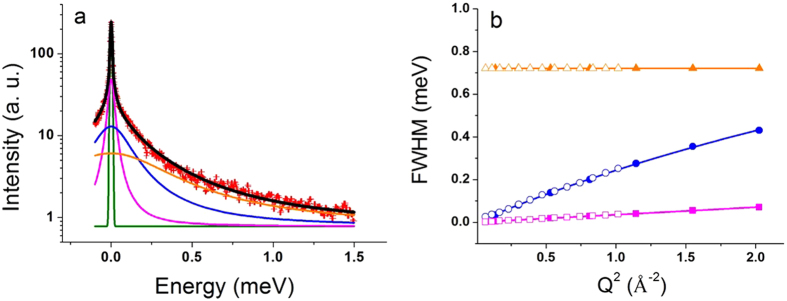
QENS data and fits. Logarithmic plot of a typical QENS spectrum (IN5) of N-LDL at 310 K and 20 bar measured at Q = 1.00 Å^−1^ (red points) and quasi-elastic contributions determined by the fit: the green line corresponds to the elastic contribution, the magenta, blue and orange lines to Lorentzian functions. The black line shows the final fit (**a**); FWHM values of the three Lorentzian distributions as function of Q^2^ obtained from IN5 (smaller Q-range, open symbols) and IN6 (full symbols) and their fits according to a translational diffusive model (magenta points), a jump-diffusion model (blue points) and a rotational diffusive model (orange points) (**b**).

QENS data analysis was performed through a “model free” approach (see Materials and Methods) by taking into account three distinct relaxation processes. It allowed to distinguish motions of different dynamical sub-groups, called populations in the following, which are associated with specific molecules such as lipids or certain molecular groups within LDL. A scenario which motion corresponds to which kind of population is suggested. Representative data of the global fit at a constant Q-value are shown in Fig. 2a (data from the neutron time-of-flight spectrometer IN5) and in the Electronic Supplementary Information (ESI) in Figure S1, which contains all TG-LDL data and fits from the neutron time-of-flight spectrometers IN5 and IN6 including residuals. For N-LDL the curves are virtually the same. To extract more precise information on the type of motion the full width half maxima (FWHM) Γ_n_ of the Lorentzian curves as function of Q^2^ were determined^21^ (Fig. 2b and ESI Table S1).

First, we analysed the data for the low pressure point (20 bar). The elastic contribution and the Lorentzian functions are shown in Fig. 2a. On IN5 we distinguished a sharp peak (magenta line), which was too narrow to be resolved on IN6 due to the lower energy resolution. The Q-dependence of the corresponding FWHM (see Fig. 2b) showed a translational motion for this population with the diffusion coefficient of D_T_ = 0.11*10^−5^ cm^2^/s. The second quasi-elastic contribution (blue line) was well resolved on both spectrometers. It corresponded to fast motions in the range of 2 to 15 ps. In particular, the analysis of the IN6 data at high Q values allowed us to detect a change of the FWHM’s behavior from a linear to an asymptotic slope at Q^2^ values above 1.3 Å^−2^ (see Fig. 2b). Thus, the Q-dependence of the FWHM could successfully be ascribed to a jump diffusion model with a diffusion coefficient of D_JD_ = 2.1*10^−5^ cm^2^/s; the residence time τ was found to be equal to 1.7 ps. The last and broadest quasi-elastic peak (orange line) could be resolved in the spectra obtained at the neutron spectrometer IN6. According to our analysis, its FWHM remained constant at all Q values, what corresponds to a rotational motion, and was determined to be Γ_rotation_ = 0.72 meV (Fig. 2b). The parameters extracted from the FWHM were identical for N-LDL and TG-LDL at 20 bar and are summarized in Table 1. We concluded that the structural differences between the two forms of LDL did not lead to any significant changes in their dynamics (on the ps time scale) at low pressure.

**Figure 3:**
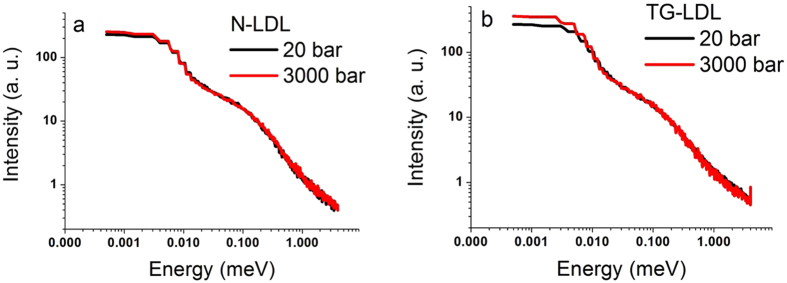
Double-logarithmic plot of QENS spectra taken on IN5. Pressure effect on N-LDL at Q = 1.00 Å^−1^ measured at 20 bar (black curve) and at 3000 bar (red curve) (**a**), same for TG-LDL (**b**).

**Figure 4:**
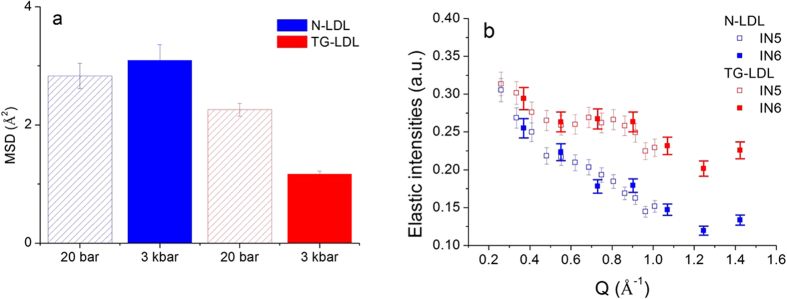
Comparison of the MSD values extracted from elastic data taken on IN13 for both samples at 20 and 3000 bar and 310 K (**a**). Elastic intensities extracted from QENS data as function of Q: N-LDL (blue) and TG-LDL (red) at 3000 bar. All data were measured at 310 K on IN5 and IN6 (**b**).

The situation changes, however, in case of HHP applied to the same samples. Analysis of the QENS data recorded at HHP (3000 bar) revealed no change in the dynamics of N-LDL (see Fig. 3a), whereas a significant difference for TG-LDL was found: the elastic part became much more pronounced, that is to say that some of its components were slowed down by pressure in this sample due to volume reduction, while the shape of the quasi-elastic broadening was not affected (see Fig. 3b). This indicates that HHP affects the elastic intensity extracted from QENS data and the Mean Square Displacements (MSD), which can be extracted from EINS measurements (Fig. 4a and ESI, Figure S2 and Table S2), but not the quasi-elastic broadening given in Table 1, which remains identical at high pressure.

The EINS results shown in Fig. 4a represent MSD values extracted from data taken on the thermal backscattering spectrometer IN13. This spectrometer has the same energy resolution as chosen on the time-of-flight spectrometer IN5, therefore the results are fully comparable. We found that the MSD of N-LDL are equal within error bars, whereas those of TG-LDL differ significantly between the two pressure points, being much lower at 3000 bar. The elastic intensities extracted from QENS data (Fig. 4b and ESI, Table S3) support the same conclusion, as no difference was found at 20 bar (Figure S2), but the values for TG-LDL at 3000 bar lie significantly above those of N-LDL. The value of the elastic intensities at highest Q-value is indicative of the proportion of immobile molecular groups, which was determined to be about 13% for N-LDL and 23% for TG-LDL.

### The shape of TG-LDL is modified by high pressure application

**Figure 5:**
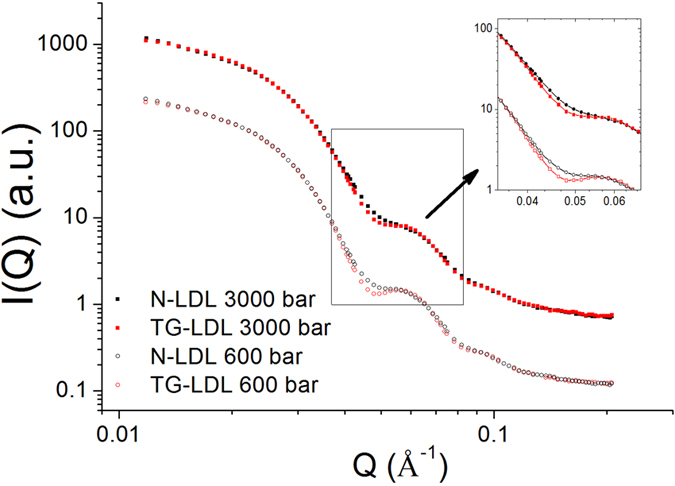
SANS curves of N-LDL and TG-LDL measured at 600 and 3000 bar and at 313 K.

To study whether HHP has an effect on the overall structural features of LDL particles we measured SANS. We started the measurements at 600 bar, as no differences were seen between 1 and 600 bar, and we will call this point hereafter “low pressure”. The Guinier region in the SANS curves indicates that both N-LDL and TG-LDL were rather monomeric systems at the two pressure values shown here (600 and 3000 bar). The radius of gyration was approximately 78 Å for both samples, which is in good agreement with previous studies^22^,^23^. The SANS curves were quite similar for all types of LDL nanoparticles under different pressure conditions, in the sense that all of them had a characteristic peak at the Q-position of 0.060 Å^−1^ (Fig. 5). Such a peak position in the reciprocal space is indicative for a characteristic radius of the system under study, which is of about 105 Å here in agreement with the radius of LDL particles of about 100 Å. The access to the internal structure of an LDL nanoparticle was limited due to missing contrast between shell and core as no deuterated lipids were incorporated.

**Figure 6:**
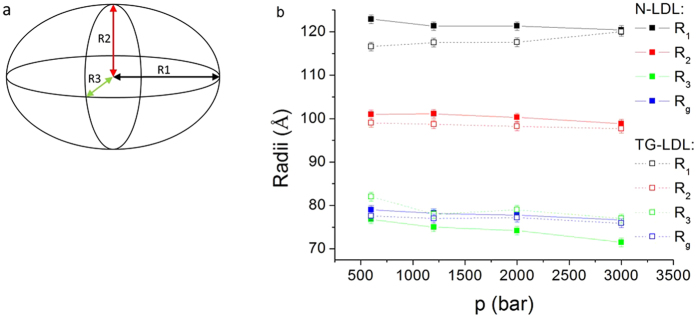
Schematic representation of the fitted ellipsoidal model with the three axes R_1_, R_2_ and R_3_ (**a**). Fitted radii of a triaxial ellipsoid extracted from the SANS curves. The continuous lines correspond to N-LDL, the dashed lines to TG-LDL (**b**).

To fit the overall structure of LDL particles we used an ellipsoidal model to extract the radius of gyration and the three radii of the ellipsoid (R_1_-R_3_) as presented in Fig. 6a. The fit indicates that the shape of N-LDL at low pressure is slightly more elongated compared to the shape of TG-LDL (see Fig. 6b and ESI Table S4). Still, the difference in the overall structure was rather small at low pressure. This is in good agreement with the QENS data, where the difference in the dynamics was also negligible at low pressure.

With increasing pressure, the shape of the SANS curve changed. For instance, the peak around Q = 0.060 Å^−1^ became less pronounced at high pressure (Fig. 5). According to our fits of the SANS curves (Fig. 6b), the smallest radius R_3_ of the ellipsoid decreased from 76.8 Å at 600 bar to 71.5 Å at 3000 bar for N-LDL and from 82.2 Å at 600 bar to 77.2 Å at 3000 bar for TG-LDL (separate graphs for the individual radii are shown in ESI Figure S3). Most interestingly, the largest radius R_1_ decreased for N-LDL and increased for TG-LDL, so that the shape of the TG-LDL at high pressure resembled much more the shape of N-LDL. The radius R_2_ and the radius of gyration were only slightly affected by pressure.

## Discussion

Analysis of the QENS data revealed three dynamical contributions reflected by the presence of three Lorentzian functions, each describing a distinct relaxation process to fit the spectra. Each function was related to a certain motion or dynamic behaviour of the investigated system. The challenge to interpret QENS results of a sample that has never been studied before consists in identifying the kind of motions and to which population they belong to, and, in the present case, to investigate how they are influenced by pressure application. We compared the obtained dynamical parameters with other results in the literature and suggest corresponding motions according to the composition of the sample to get a better picture of the functioning of the biomolecule.

**Figure 7:**
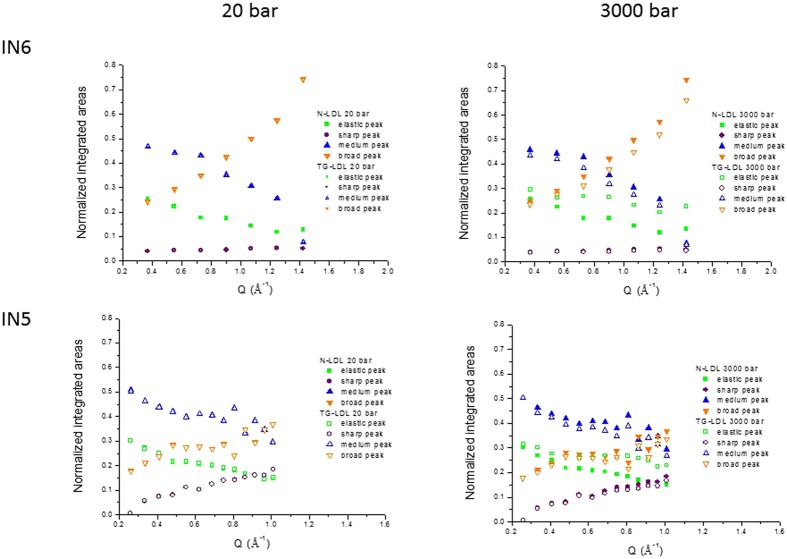
Spectral weights or Q dependence of the integrated areas of the elastic peak and the Lorentzian functions on IN5 and IN6 for both pressure values. For 20 bar the data points are almost completely superposed.

First, we calculated the hydrogen content of the different components of the samples (see Table 2) to get information about their corresponding scattering contribution and consequently about the ratios of the components which participate to the different motions. Second, we determined the normalised spectral weights of the scattering contributions of the different populations (see Fig. 7) for both instruments and pressures.

The spectral weights represent the Q-dependence of the integrated areas of the elastic peak and the Lorentzian functions and are indicative for the contribution of the corresponding population at a given Q value (see Fig. 7). However, the total spectral weight corresponding to a certain part of the sample has to be Q-independent. Indeed, the contributions from the medium and broad peak and from the elastic and sharp peak appear pairwise highly correlated as they are almost symmetric with respect to an average line, what indicates that they could belong to the same population, which executes two motions simultaneously.

Without extensive contrast variation using partly and/or fully deuterated components it is not possible to assign more precisely the different motions in such complex systems as are lipoproteins. However, we evaluated that an exchange of a hydrogenated lipid population with a deuterated one would be too small to lead to significant contrast variation of these components and to permit a more precise assignment of the motions. On the other hand, several kinds of lipids were already investigated by neutron scattering methods, for instance DMPC, POPC^25^, DOPS and DOPC^26^. They showed up at least two different motions which resulted in characteristics of the same order of magnitude as those found in the present work, that is to say a jump diffusion coefficient of about 2.0 × 10^−5^ cm^2^/s together with a residence time of about 1 ps and another translational diffusion coefficient, associated to a centre-of-mass diffusion of the lipids, which is 10–20 times smaller. Without going into details to which part of the lipids such motions can be precisely assigned here, we emphasize that diffusional or confined motions of this order of magnitude exist in lipids at temperatures around 310 K and that we can refine these in the analysis performed. In addition, different kind of lipids behave differently and their motions are highly dependent on the presence of cholesterol^27^,^28^. It is however not possible to distinguish all movements of such a highly complex system, but we present a serious proposition to describe LDL under HHP which includes both dynamical and structural effects.

The other relaxation process is associated with rotational diffusion according to the form of the FWHM Γ_rotation_, which has a width of 0.72 meV very similar to values found in deep sea prokaryotes^29^ or in neural tissue^30^. Fast rotational diffusion could originate from different locations in the sample, for instance from protons in CH_2_ groups within lipids and the apoB100 protein. It is thus representative for typical motions in the sample, which can be seen in the time window of the spectrometer. Surprisingly, all these movements are not much influenced by pressure application. However, the proportion of molecular groups participating to these motions changes. In the present case, the dynamical populations are almost constant for both samples in spite of pressure application, but the proportion of the elastic peak is enhanced for TG-LDL, in compliance with the EINS and elastic intensities’ results, at the expense of the population undergoing rotational diffusion (by about 7%). Therefore, some of the slowest detected motions, corresponding to the local movements within the TG-LDL particle, are slowed down by HHP and become finally undetectable by QENS.

Consequently, our results indicate that the dynamic landscape of LDL includes motions of lipids and rotational diffusion of smaller molecular sub-groups as for instance CH_2_ groups. Indeed, nearly 80% of LDL are lipids and cholesterol (see Table 2), but their motions can be influenced by the presence of the protein. As shown recently by Knoll *et al*.^26^, proteins can render lipids more flexible or in contrary reduce the dynamics of parts of them due to more or less confinement compared to the pure system. According to the QENS analysis we could not detect any changes of the quasi-elastic motions neither among the samples nor with the measured pressure points, but the proportion of molecular groups participating to the dynamics has been found to be different.

**Figure 8:**
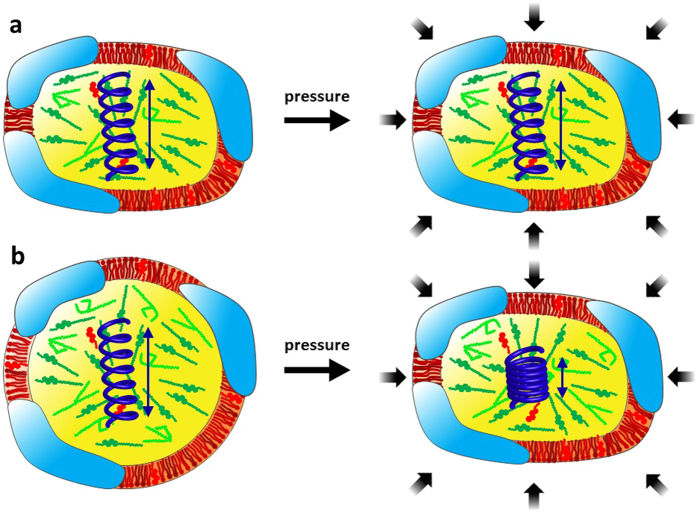
Sketch of the effect of high hydrostatic pressure on LDL particles (caption see Fig. 1); N-LDL (**a**) and TG-LDL (**b**). The blue spring symbolizes the altered dynamics within the particle.

The observation that the geometry of the motion changes, with a reduction in the typical confinement size in TG-LDL, could be expected according to Le Chatelier’s principle, saying that compression by HHP will favor a volume reduction and thus lead to reduced dynamics. This behavior was also reflected in the structural data, as shown by SANS. N-LDL appeared slightly elongated and had a discoid shape at the lowest pressure point, whereas TG-LDL was more spherical at the same conditions. Similar characteristics for normolipidemic and triglyceride-rich LDL particles have already been shown with cryo-electron microscopy before^31^,^32^. At HHP we found that the N-LDL particles did not change their overall shape and stayed in an ellipsoidal conformation. However, the TG-LDL particles adapted their shape from a more spherical to a more elongated form similar to N-LDL (see Fig. 8). In both samples the particle partial molar volume decreased and the surface-to-volume ratio increased with pressure, but the changes were less pronounced for TG-LDL, which on the other hand became deformed. The surface-to-volume ratio change in LDL could be due to differences in the compressibility between shell and core. Other reasons could be a pressure dependent conformational change of the apolipoprotein, which could become more stretched or flat by pressure to cover a larger surface area. Another effect could be a possible lipid phase change induced by pressure or a different equilibrium of unesterified cholesterol between the core and the phospholipid membrane, which could also lead to a different increase in surface-to-volume ratio. We speculate that a combination of several effects might occur. Such variation due to the protein moiety would be in compliance with the dynamical findings which point towards a higher pressure sensitivity of fast local motions within the apoB100 protein or CH_2_ subgroups.

Taken together, the N-LDL, representing the normolipidemic healthy system seemed to cope much better with the externally increased pressure stress than TG-LDL, which mimicked the hypertriglyceridemic form associated to pathological health conditions. The distinctions in the dynamics between N-LDL and TG-LDL became clearly visible at HHP: TG-LDL showed a lower MSD value indicative for the reduced flexibility of motions and increased stiffness. At low pressure TG-LDL particles were rather spherical but approached the ellipsoidal morphology of N-LDL at high hydrostatic pressure.

It could be shown that especially the apoB100 protein anchored in an artificial lipid interface is highly flexible under surface pressure and can cope for vast structural changes of the surface layer. Parts of the apoB100 can be pushed off the surface during compression and readsorb when the surface expands^33^. Moreover, the whole protein structure has a very flexible nature as seen in a low-resolution reconstruction of a SANS study of detergent solubilized apoB100^19^. The intrinsic mobility of apoB100 is certainly of major importance when it comes to structural adaptation. The influence of a TG-rich lipid core composition on LDL shape, as shown by cryo-electron microscopy^32^, is also reflected in the dynamic behaviour of LDL as shown by our results. We suggest that the structural adaptation of LDL with a TG-rich core is facilitated by the highly flexible apoB100.

In conclusion, our results indicate that LDL particles withstand HHP quite well, however, a TG-rich core lipid composition seems to have an impact on the molecular dynamics of LDL particles under pressure.

## Materials and Methods

### Sample preparation for neutron scattering experiments

LDL was isolated from human blood plasma by applying a multiple-step density gradient ultracentrifugation^1^. Blood plasma was obtained from the Department of Blood Group Serology and Transfusion Medicine of the University Hospital Graz (Graz, Austria) after written informed consent, according to a protocol approved by the Institutional Review Board of the Medical University of Graz. The blood plasma was free of pathogens (HBV, HCV, HIV). The isolated LDL was extensively dialyzed against buffer (10 mM NaPi, 0.1% EDTA, pH 7.4), concentrated with Amicon Ultra-15 centrifugal filter units (cut-off 100 kDa) and characterized for its protein and lipid composition. Sample purity was checked with SDS-PAGE. Chemical compositions and measured concentrations of normolipidemic N-LDL and triglyceride-rich TG-LDL are given in the ESI, Table S5 and Table S6.

### Elastic and quasi-elastic neutron scattering experiments and data analysis

LDL samples were measured at a constant temperature of 310 K, at 20 and 3000 bar. The samples were in solution to ensure a homogeneous transmission of hydrostatic pressure and to highlight the single particle dynamics. For the dynamical high pressure experiments, we took advantage of equipment developed and validated within the last years at the neutron scattering facility Institut Laue Langevin (ILL, Grenoble, France) for measurements of samples in solution^34^. We used the time-of-flight spectrometers IN5^35^ and IN6^36^ as well as the thermal backscattering spectrometer IN13^37^ at the ILL which permitted to combine elastic and quasi-elastic scattering investigations and to use two different time windows to identify various types of motions at the atomic scale. The instrumental energy resolutions were determined by a vanadium measurement, which is a completely incoherent scatterer, to be about 0.01 meV for IN5 and IN13 and 0.075 meV for IN6, corresponding to time windows of about 100 ps and 15 ps, respectively. The shorter time scale of IN6 permits to identify fast motions, whereas the longer time scale of IN5 and IN13 allows to see slower motions arising from the lipids and other local motions. We are summarising the instrumental characteristics of IN5, IN6 and IN13 in the ESI Table S7.

In order to quantify the contribution of the solvent, measurements of LDL in buffer and pure buffer (10 mM NaPi, 0.1% EDTA, pH 7.4 in D_2_O) were carried out separately. All measurements were performed at 20 bar and 3000 bar. Transmission values were measured on IN13. We got 89.0% for the empty cell transmission, 82.7% for the buffer and 74.4% and 77.0% for the N-LDL and TG-LDL samples, respectively. Each sample spectrum was corrected with respect to the incoming flux. Both the sample and the buffer were first corrected for the empty cell contribution by taking into account their respective absorptions, then the buffer was subtracted from the sample in a second step. The spectra were then normalized using the LAMP software available at ILL^38^. Absorption correction was based on the correction formula of Paalman-Pings^39^. To reduce the sample volume and to avoid multiple scattering, we included a cylindrical aluminum insert of 3 cm height and 4 mm diameter into the inner hollow volume of 6 mm diameter of the sample cell. Moreover, we used a cadmium mask limiting the region irradiated by the neutron beam to the height of 3 cm containing the aluminum insert, although the total height of the sample volume was about 5 cm. When increasing the pressure, more LDL particles were pushed into the beam, but we estimated the increase to a few percent, only, as most of the sample volume was outside the beam.

### Elastic neutron scattering experiments

Elastic incoherent neutron scattering (EINS) data were collected on the thermal backscattering spectrometer IN13. In a first approximation, the Gaussian approximation^40^, which assumes that the distribution of the atoms around their average position follows a Gaussian distribution, the scattered intensity is given by the dynamic structure factor at zero energy exchange


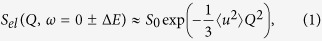


where < u^2^ > are the average atomic mean square displacements (MSD). ω and Q are the energy and momentum exchanged between the neutron and an atomic nucleus of the sample in units of ħ, respectively, and ΔE is the half width half maximum (HWHM) of the instrumental energy resolution. For Q → 0, the approximation is strictly valid, and it holds up to < u^2^ > Q^2^ ≈1 41. The MSD can be obtained for each pressure value by the slope of the semi-logarithmic plot of the incoherent scattering function through


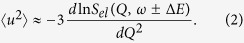


The MSD values are a measure for the flexibility of the biological system at a given condition of temperature or pressure^42^.

### Quasi-elastic neutron scattering experiments

Quasi-elastic neutron scattering (QENS), which accounts for small energy exchanges between neutron and target, can be described by the incoherent double-differential cross section^21^:


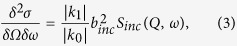


where *k*_*0*_ and *k*_*1*_ are the wave vectors of the incident and scattered neutron, respectively. b_inc_ is the incoherent scattering length and S_inc_(*Q*, ω) is the incoherent scattering function. In a real experiment, only the experimental scattering function S_exp_ is accessible which contains the instrumental resolution function R(*Q*, ω):


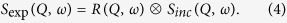


To get an idea about S_inc_(*Q*, ω), which is the theoretical model function describing the dynamics of the sample, neutron spectra have therefore to be deconvoluted by the experimentally obtained resolution function. To describe the scattering function, the following generic equation is applied:


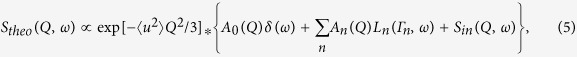


that contains the sum of three main contributions: (1) a factor proportional to δ(ω)– corresponding to the elastic component when no energy is exchanged; (2) the quasi-elastic contribution is represented by a sum of Lorentzian-shaped components L_n_(Γ_n_, ω), where Γ_n_ is the full-width at half maximum (FWHM); and (3) the inelastic part S_in_(*Q*, ω), corresponding to collective low-frequency vibrational motions. The term 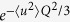 is the Debye-Waller factor characterized by the vibrational MSD < u^2^ > . The Q-dependent amplitudes A_0_(*Q*) and A_n_(*Q*) in equation (5) denote the elastic and the quasi-elastic incoherent structure factors (EISF and QISF), respectively. One can show^21^ that the EISF A_0_(Q) is given by the elastic intensity divided by the sum of elastic and quasi-elastic intensities at t → ∞ and it is indicative of the geometry of the diffusional process. Generally, if only long-range diffusive motions were present, it vanishes except for Q = 0. For the exclusive rotational diffusion of a particle, the EISF is unity at Q = 0 and falls to a minimum at a Q value which is inversely related to the radius of gyration of the rotating particle and directly related to the proportion of particles seen as immobile. In the present case, we have different motional contributions, which are treated additively.

### QENS model free analysis approach

To fit the QENS spectra by such a model scattering function a couple of approximations are applied after verification that they are well fulfilled for the two instruments: (1) the resolution function R(*Q*, ω) is assumed to have a Gaussian shape; and (2) inelastic contributions can be neglected within the energy transfer ranges considered here. Therefore, each spectrum was fitted by a sum of an elastic Gaussian function contribution and several Voigt functions, which were representing the convolution of a Lorentzian function with the Gaussian resolution peak.

Theoretically, the sum over the Lorentzian contributions in eq. (5) goes to infinity, but in practice the sum has to be limited to the minimum number of contributions which describe the data reasonably well and permit a physical interpretation of the results. Therefore, it was necessary to make a preliminary analysis in order to figure out the number of independent quasi-elastic contributions contained in the QENS spectra through an inverse Fourier Transformation of the scattering function S(*Q*, ω) to the intermediate scattering function I(Q, t). Such approach permits to plot the spectra obtained on IN5 and IN6 on the same graphics and to verify how many relaxation processes are necessary to describe the whole curve^43^ (see ESI Figure S4, at Q = 0.73 Å^−1^). The spectra originating from the two instruments show a discrepancy in the two overlapping regions. We thus determined three distinct relaxation processes to describe the QENS spectra in a satisfactory way.

QENS data analysis as presented here, as to say without any further assumption about the composition of the sample and the detected motions, is commonly called a “model free” approach. As mentioned above we determined three distinct relaxation processes to be taken into account, which were more or less well resolved on both instruments. Therefore, we used the data of IN5 and IN6 simultaneously for a global fit with Origin (http://www.originlab.com/) to gain in precision. IN5 and IN6 do not only differ in energy resolution, but also in the so-called dynamical ranges, that is in the accessible Q-ranges. The only Q-values existing on both instruments are (0.33, 0.55, 0.74, 0.91 and 1.0) Å^−1^ (cf. Fig. 2b and Fig. 4b), so we started by fitting them first for the two data sets simultaneously. In a second step, we fitted the intermediate Q-values of IN5 and the higher Q-values of IN6 to get consistent results. This is what we call a global fit.

The FWHM Γ_n_ of the Lorentzian curves as function of Q^2^ provided more precise information on the type of motion^21^. If some particles were performing Brownian motion without any restriction in space, the corresponding FWHM would present a linear increase with Q^2^ crossing the origin at Q → 0 and obeying the relation Γ_T_ = D_T_ Q^2^. If there were interactions between particles, the FWHM would start with a very similar slope at low Q^2^, but would flatten to a constant value at high Q^2^ 44. Such behavior is characteristic for jump-diffusion, where the particles perform a translational diffusive motion for a short time with a diffusion coefficient D_JD_ and then vibrate at their position for a certain time τ, called residence time, after which they perform another jump. Rotational diffusion corresponds typically to a FWHM which is constant in Q. To the best of our knowledge LDL particles have never been studied by QENS, thus we applied the model free approach, as no information was available about motions which could be distinguished, and used models (free Brownian diffusion^44^, jump diffusion^45^ and rotational diffusion^21^) for the fits of the FWHM of the Lorentzian curves.

### SANS experiments and data analysis

SANS experiments were performed on the SANS-II beamline^46^ at the Paul-Scherrer-Institute (PSI, Villigen, Switzerland) as function of pressure in the range from 20 bar to 3000 bar. For N-LDL we could not observe significant changes in the SANS curves below 600 bar (see ESI Figure S5), therefore 600 bar were taken as starting point.

High pressure equipment specifically developed for SANS experiments was used^47^. The sample thickness was fixed and did not change with pressure (less than 10 μm, which corresponds to a 1% variation in path length and, thus, in transmission). To get access to the broad Q-range of 0.006 to 0.12 Å^−1^ corresponding best to structural dimensions contained in the LDL particles in the reciprocal space, the data was collected at three different sample-detector distances. We also re-measured our samples after HHP application to check for denaturation. The curves were fully reproducible and the samples were still clear and did not show any turbidity. All measurements were performed in D_2_O buffer (10 mM NaPi, 0.1% EDTA, pH 7.4 in D_2_O).

The radius of gyration R_g_, which is a measure for the moment of inertia of LDL, was evaluated according to the Guinier approximation^48^ from the experimental scattering curves:


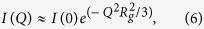


which is valid for small Q values such that Q*R_g_ < 1.5. Here I(0) is the forward scattering intensity, which is a shape independent function of the total scattering power of the sample.

For further analysis, we used the master formula of SANS for a diluted solution of monodisperse particles:


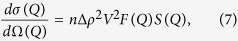


where n is the number of particles, ∆ρ is the difference in scattering length density between the particles and the solvent and V is the volume of the particles. F(Q) is the form factor, which is a function of the averaged shape and the averaged size of the scattering particles. The effective structure factor is called S(Q) and for diluted solutions with no interacting particles equal to 1. The scattering intensity pattern was simulated by the SASView software developed at NIST^49^. In our approximation, the scattering contributions of LDL nanoparticles were modelled as the intensity profile of a triaxial ellipsoid^50^. In this analysis we did some approximations: (1) the average scattering length density of the LDL nanoparticle was taken as about 3.7 10^−6^ Å^−2^ 51; (2) the polydispersity in radius was assumed to be the same for N-LDL and TG-LDL samples and accounts for about 10–15%.

## Additional Information

**How to cite this article**: Golub, M. *et al*. High hydrostatic pressure specifically affects molecular dynamics and shape of low-density lipoprotein particles. *Sci. Rep.* **7**, 46034; doi: 10.1038/srep46034 (2017).

**Publisher's note:** Springer Nature remains neutral with regard to jurisdictional claims in published maps and institutional affiliations.

## Supplementary Material

Supplementary Information

## Figures and Tables

**Figure 1:**
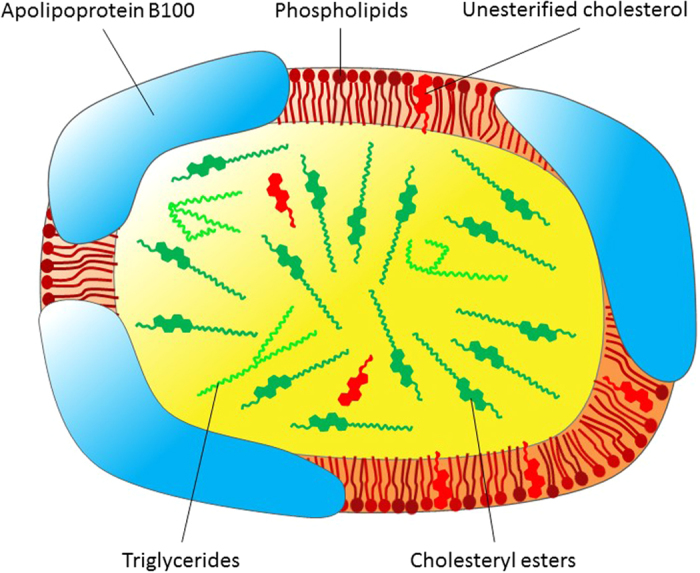
Scheme of an LDL particle including the various components.

**Table 1:** Parameters extracted from the global fits of the QENS curves.

D_T_	0.11 ± 0.02 (10^−5^ cm^2^/s)
D_JD_	2.1 ± 0.1 (10^−5^ cm^2^/s)
τ	1.7 ± 0.1 (ps)
Γ_rotation_	0.72 ± 0.05 (meV)

The parameters were identical for N-LDL and TG-LDL samples at low (20 bar) and high hydrostatic pressure (3000 bar).

**Table 2:** ^1^H-content (wt%) of the different components of LDL.

Component	QENS	
	N-LDL	TG-LDL
Protein	14.1	12.7
Phospholipids	22.5	20.2
Unesterified cholesterol	9.2	6.7
Cholesteryl esters	49.0	48.1
Triglycerides	5.2	12.2

The average ^1^H-content of the single components was calculated by using the chemical formulas and molecular masses. The relative composition of fatty acid residues in phospholipids, cholesteryl esters and triglycerides^24^ was considered. For the calculation of the ^1^H-content in the QENS samples the actual chemical composition was taken from Table S5.
