# Supplementary material for: High hydrostatic pressure specifically affects molecular dynamics and shape of low-density lipoprotein particles
Source: Sci Rep. 2017 Apr 6;7:46034. doi: 10.1038/srep46034 (PMC5382586; doi:10.1038/srep46034)
Supplement: Supplementary Information [file srep46034-s1.doc]

**Electronic supplementary information**

High hydrostatic pressure specifically affects molecular dynamics and shape of low-density lipoprotein particles

M. Golub, B. Lehofer, N. Martinez, J. Ollivier, J. Kohlbrecher, R. Prassl, J. Peters

IN5

|  | TG-LDL sample | |
| --- | --- | --- |
| Q (Å^-1^) | 20 bar | 3000 bar |
| 0.25 | 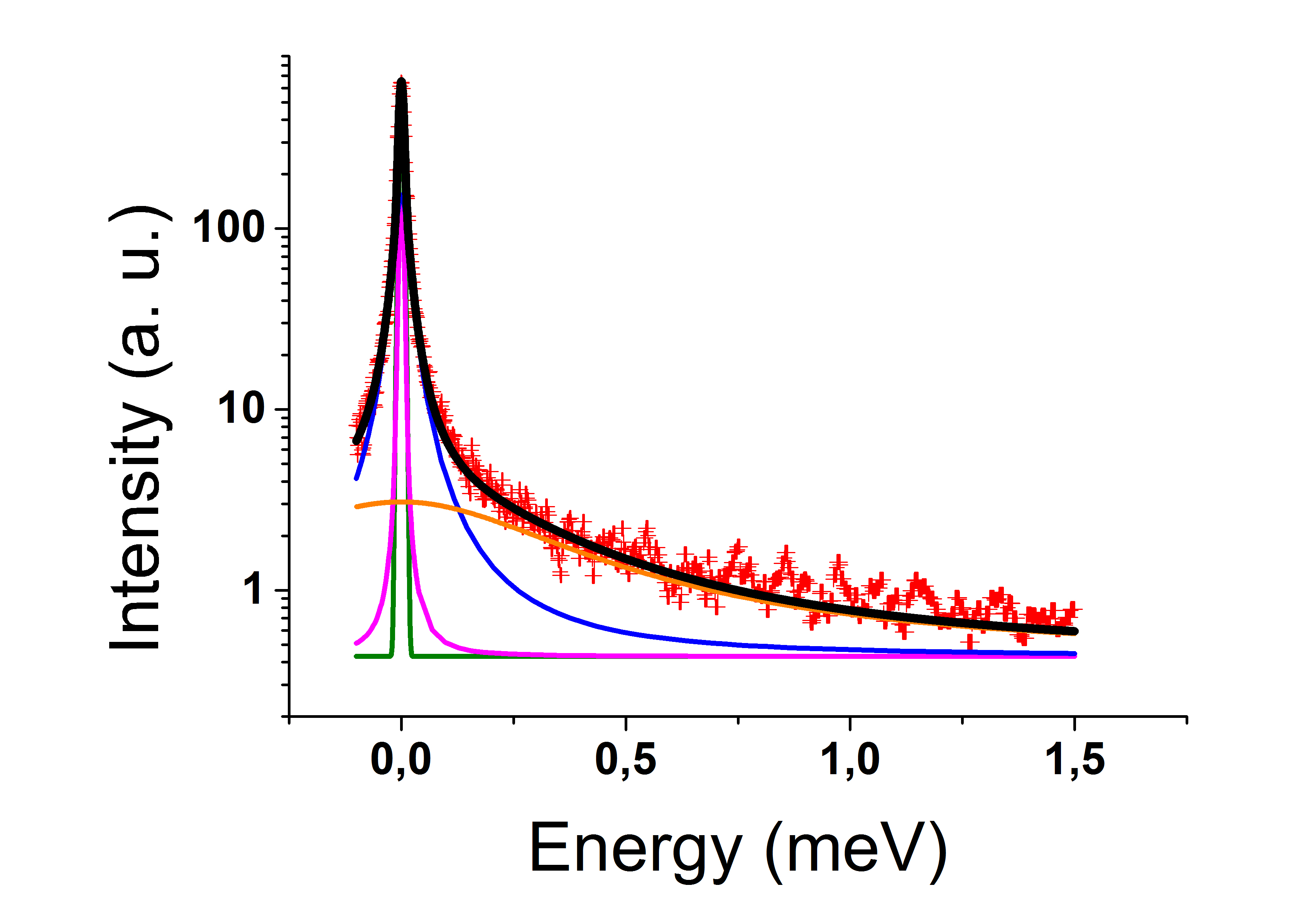 | 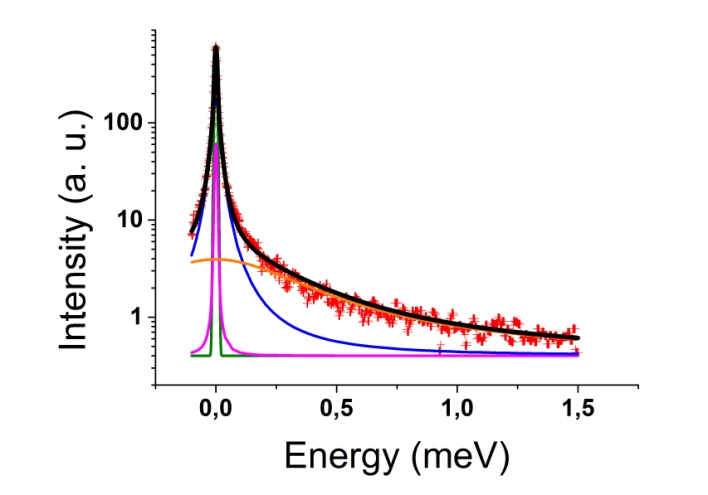 |
|  | 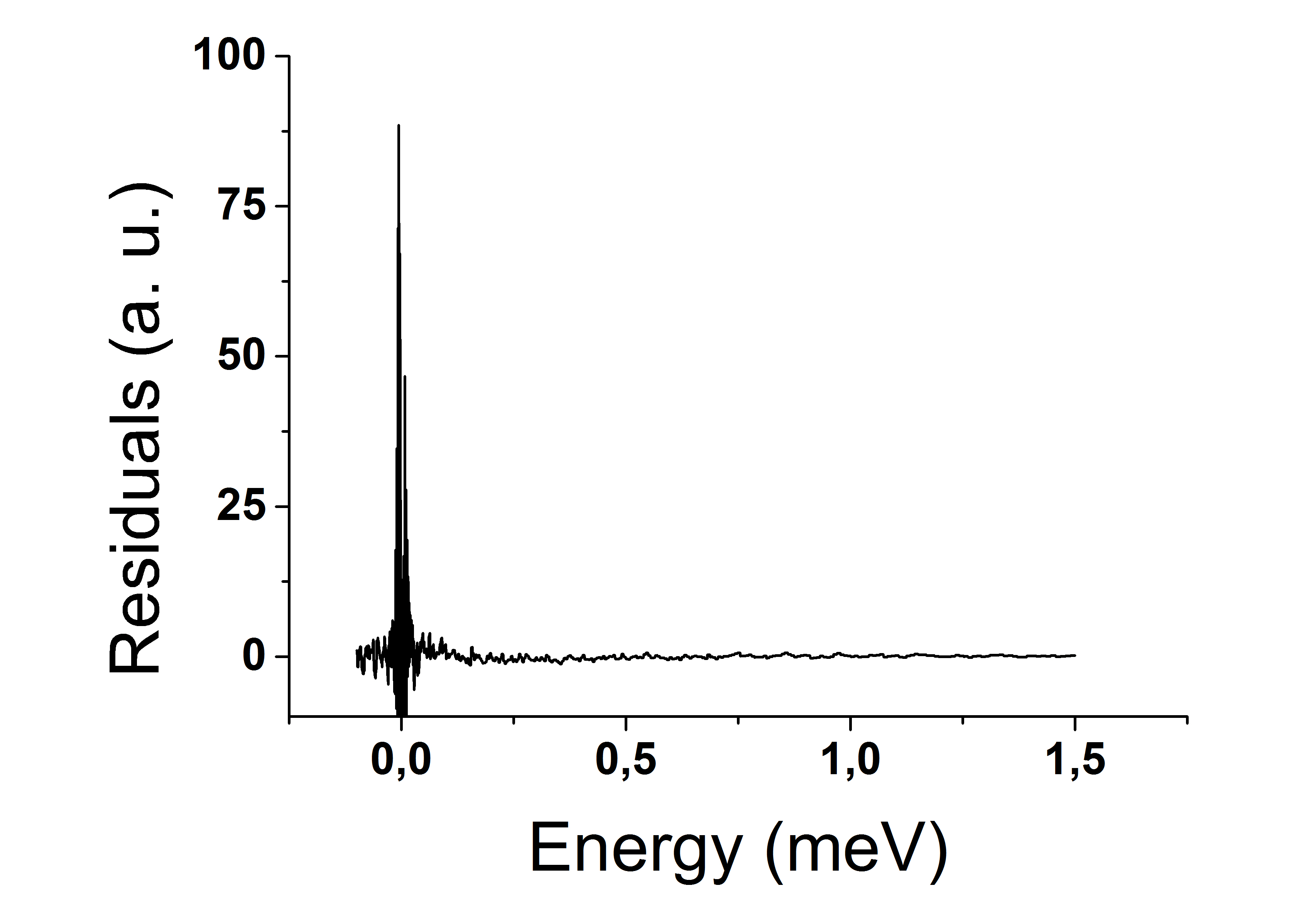 | 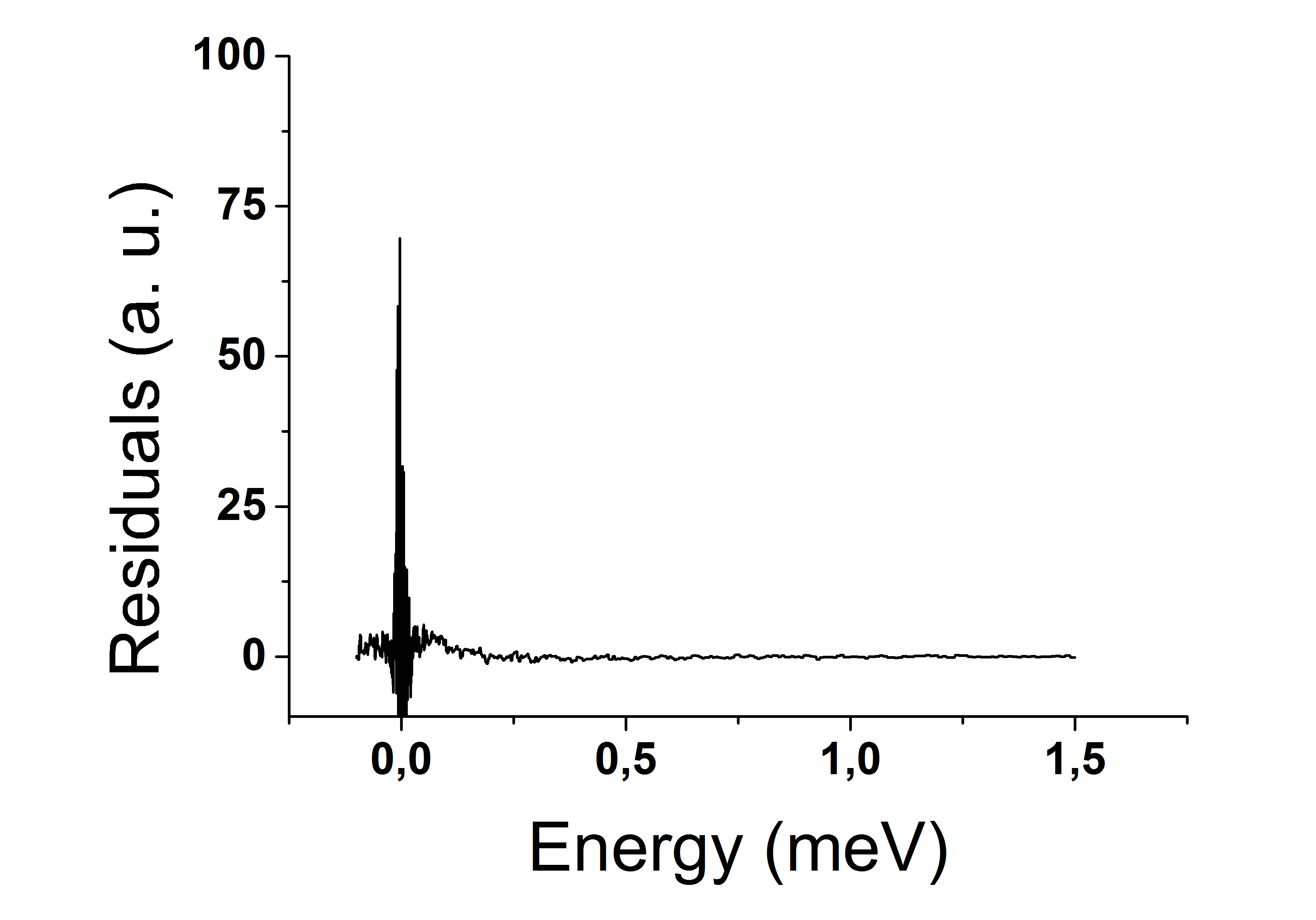 |
| 0.33 | 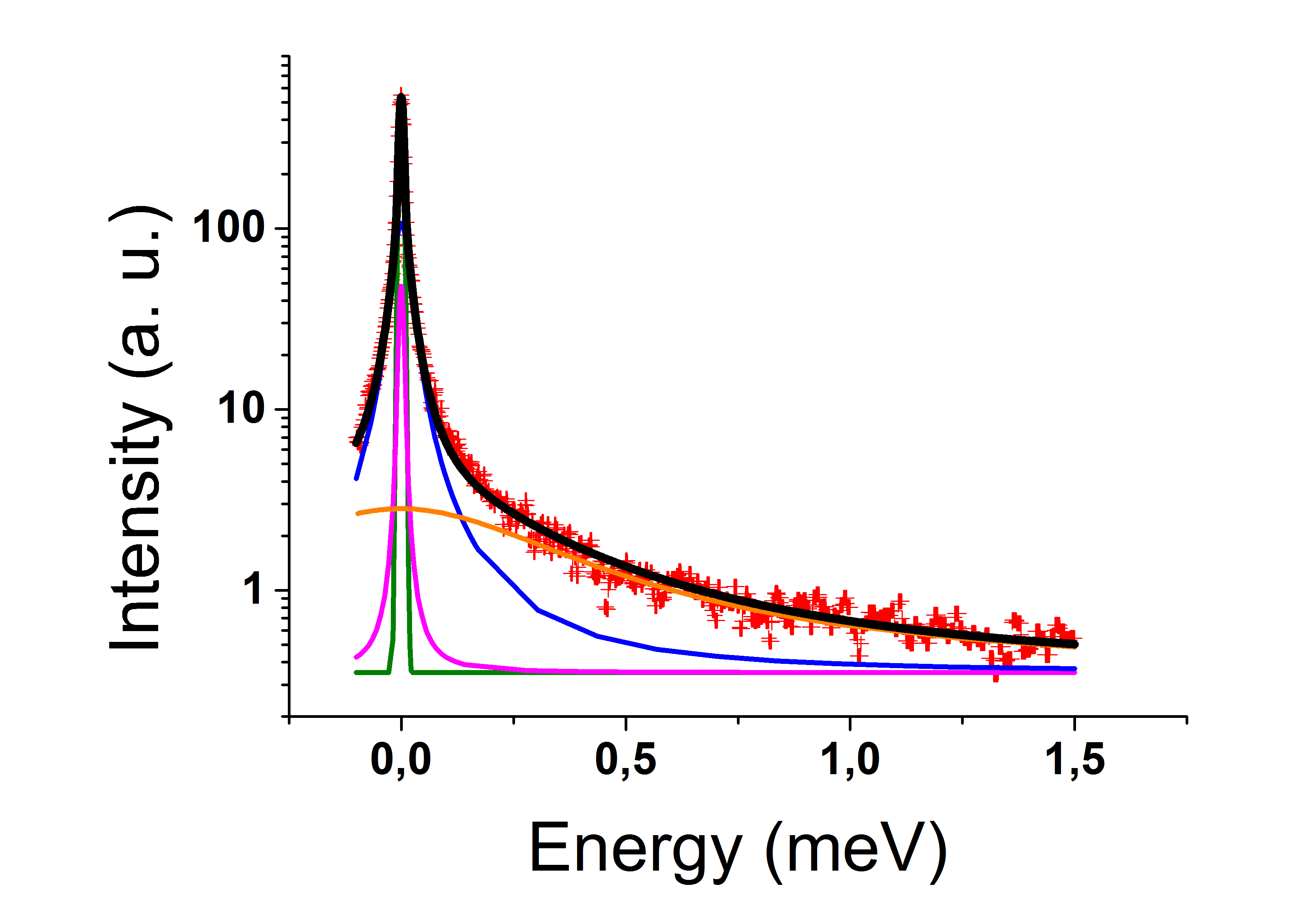 | 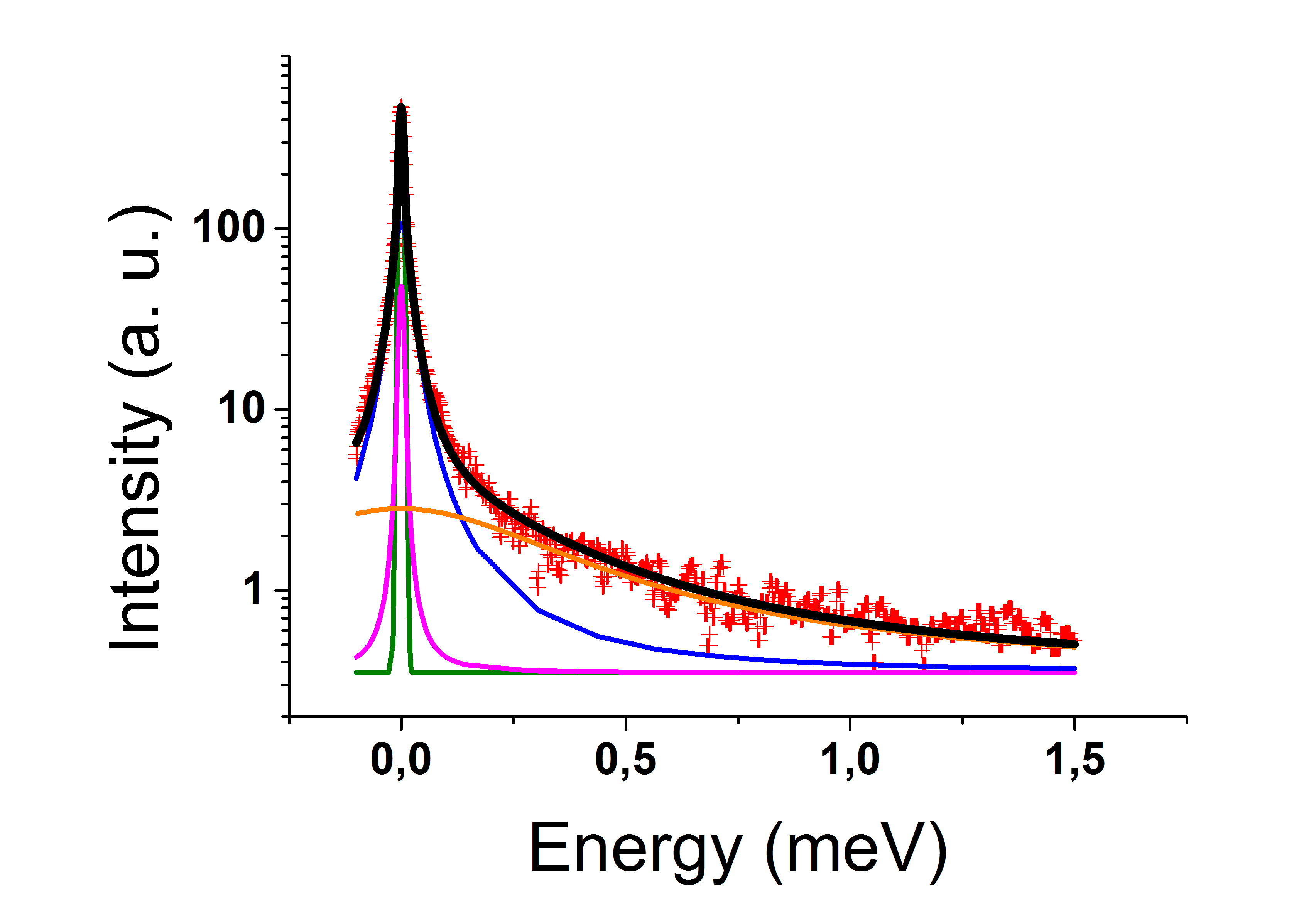 |
|  | 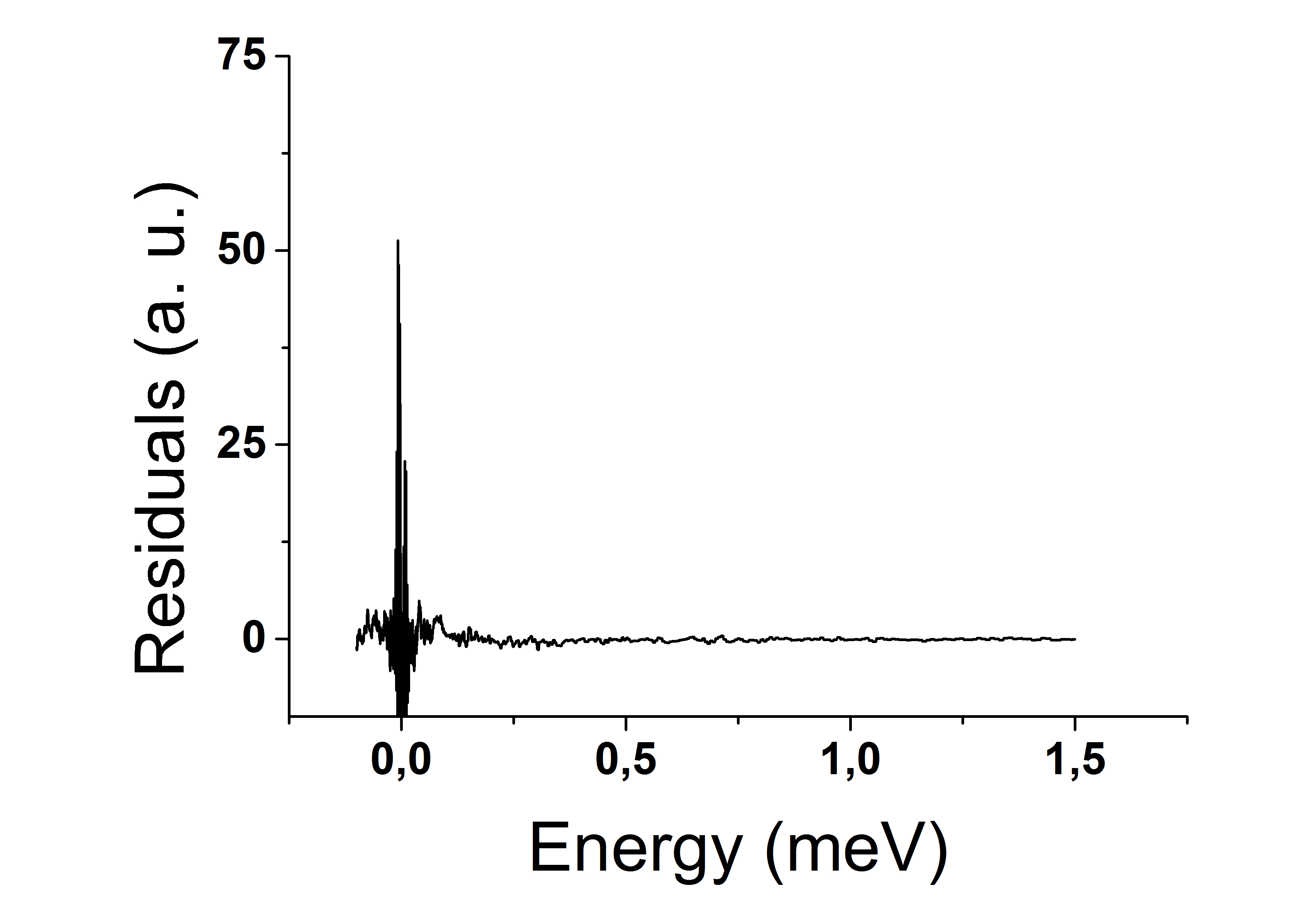 | 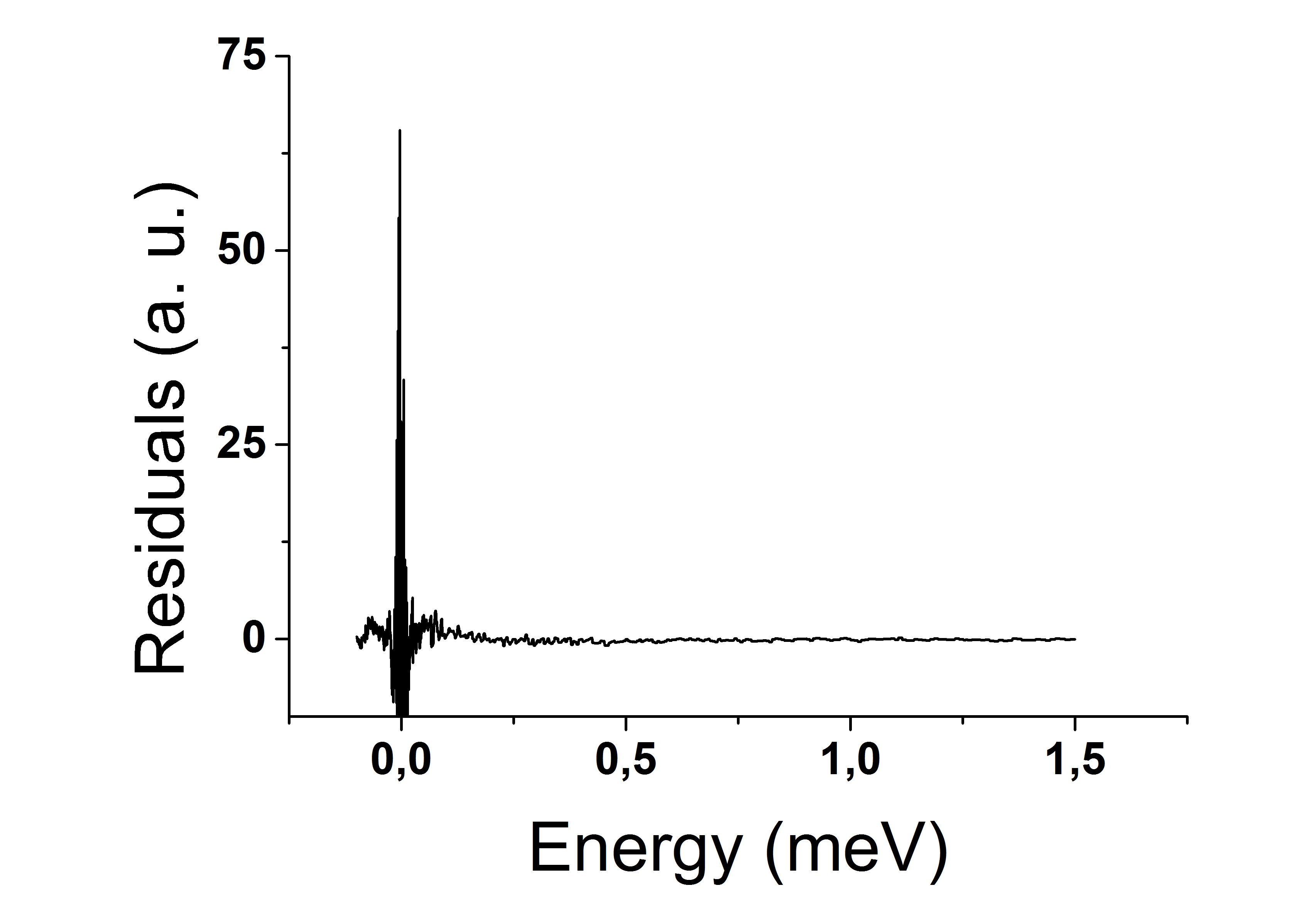 |
| 0.4 | 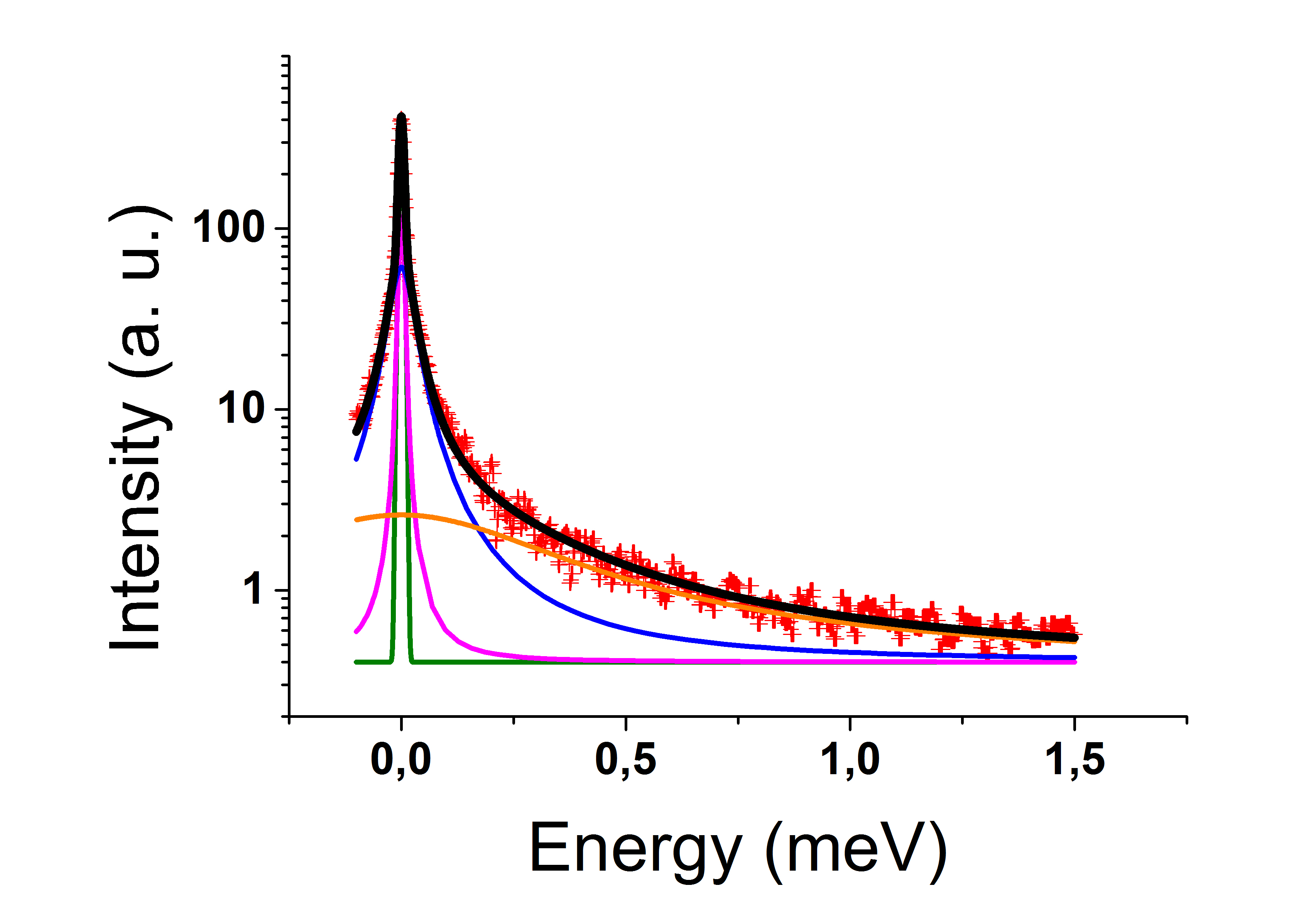 | 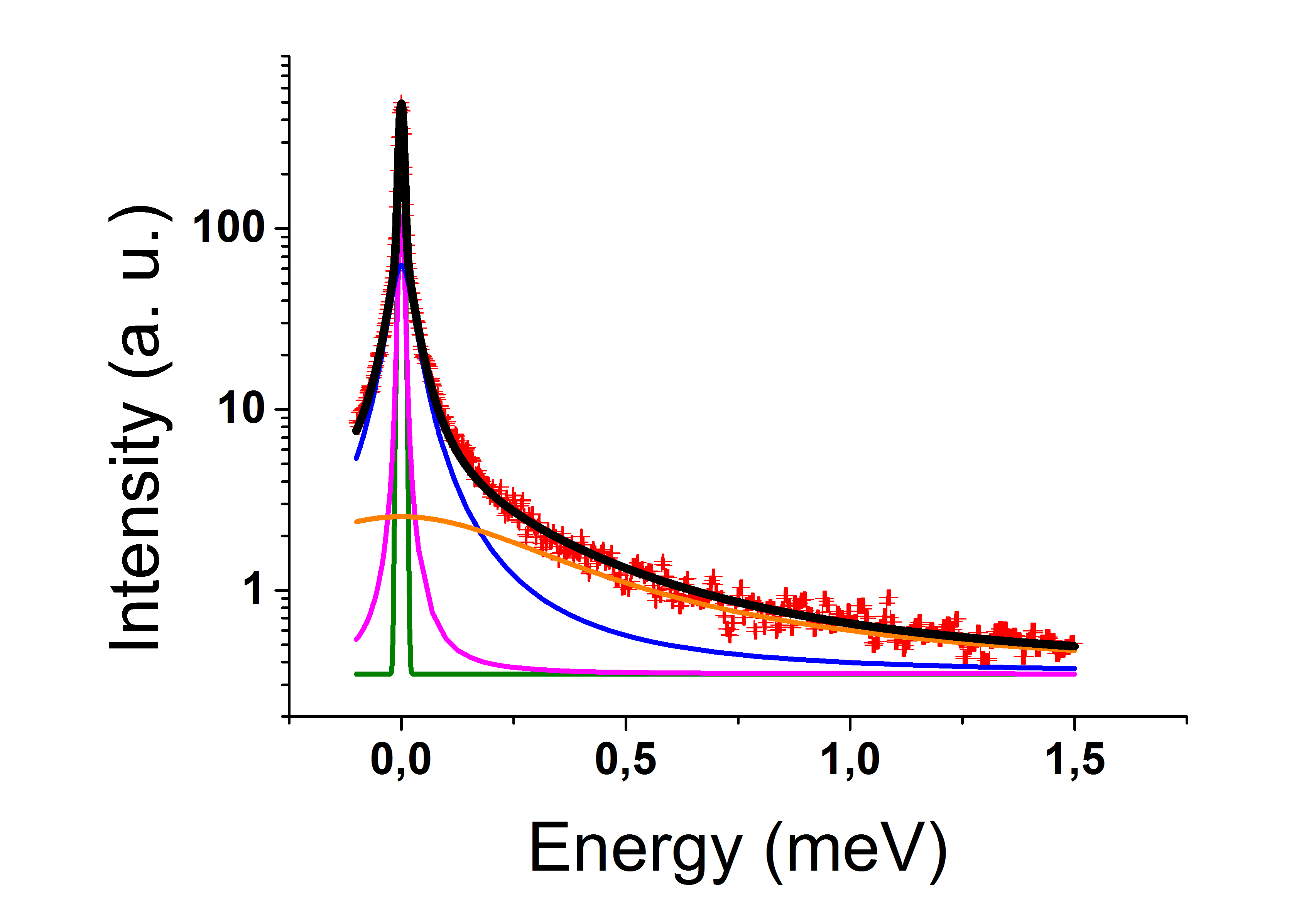 |
|  | 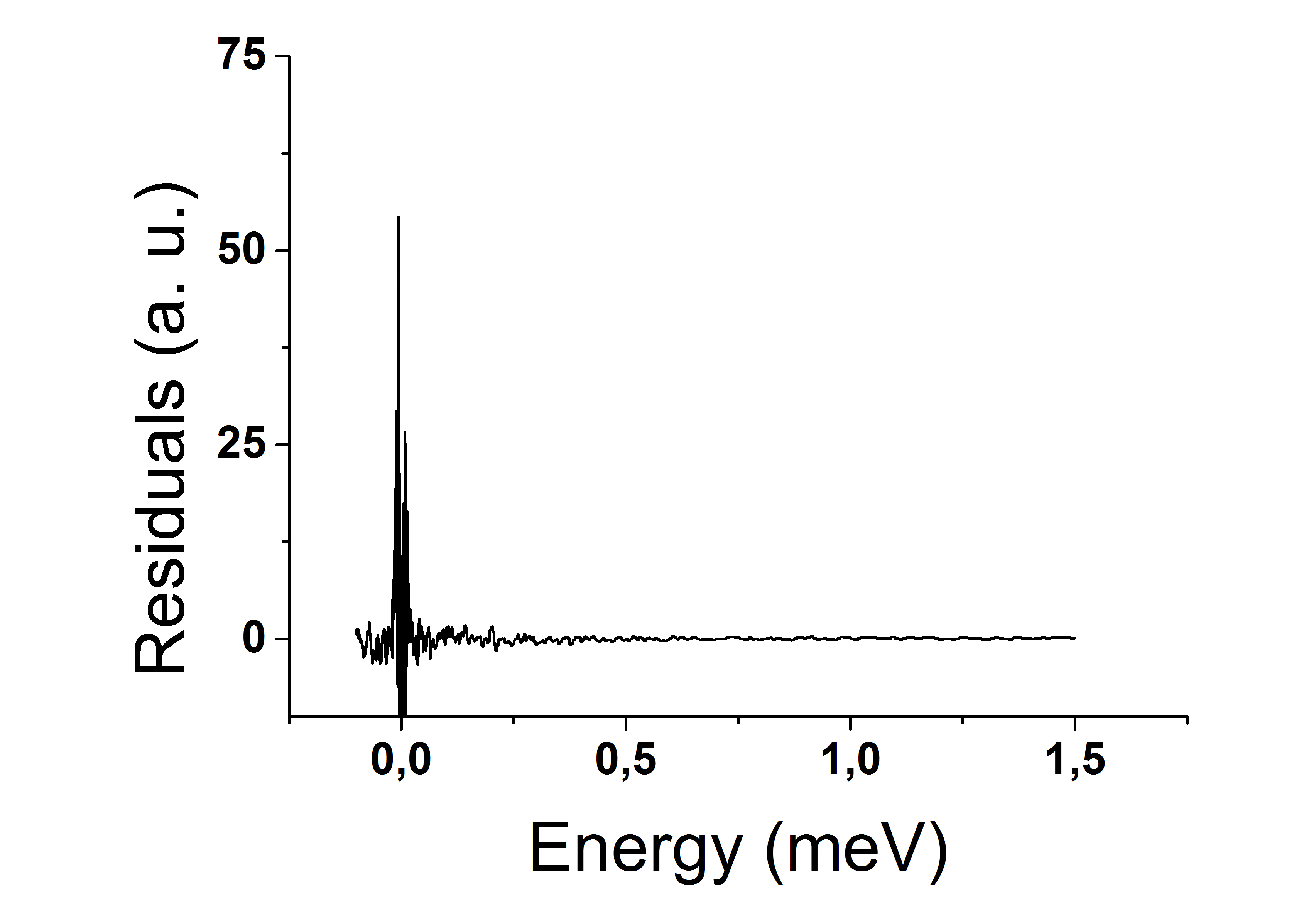 | 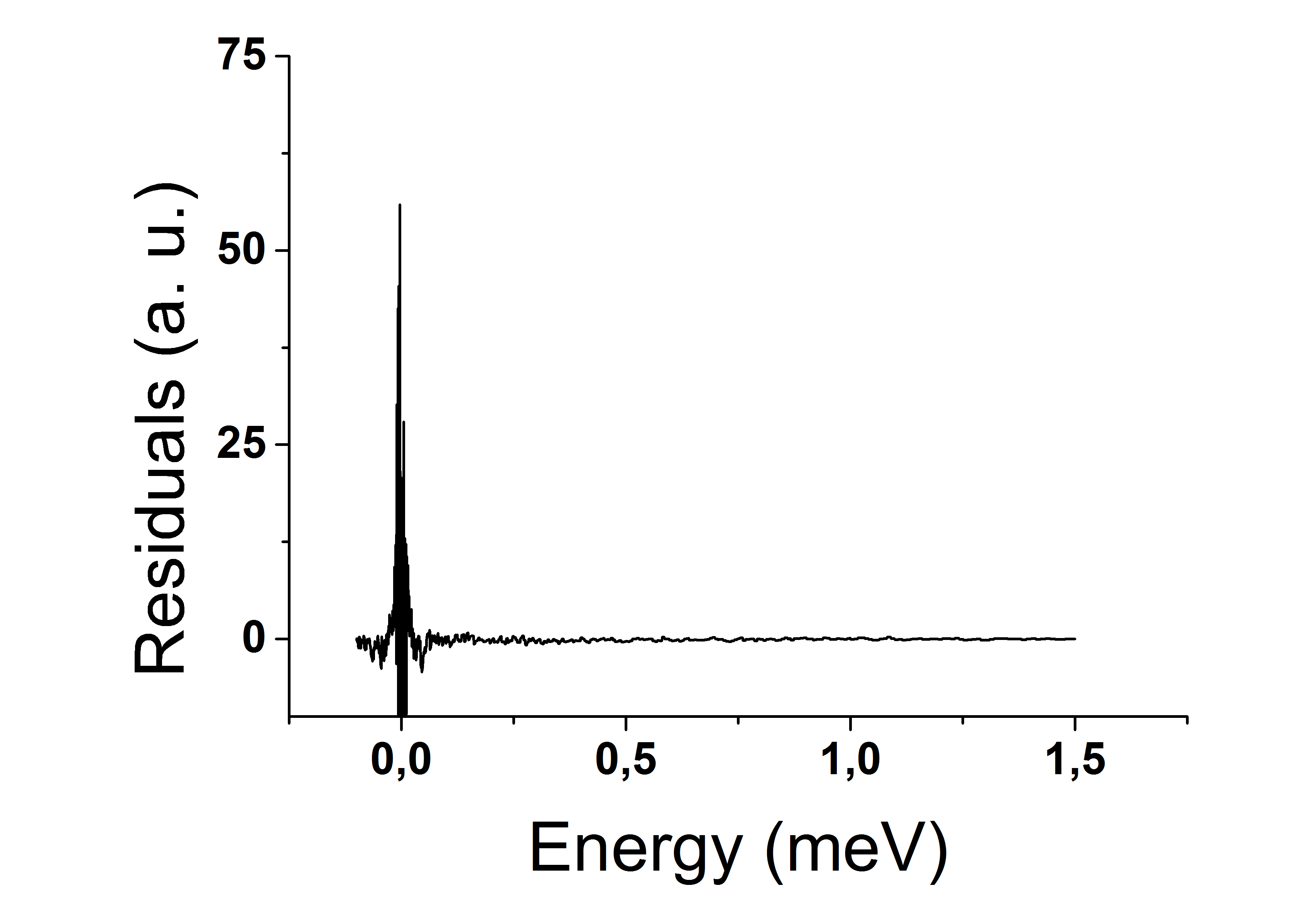 |
| 0.48 | 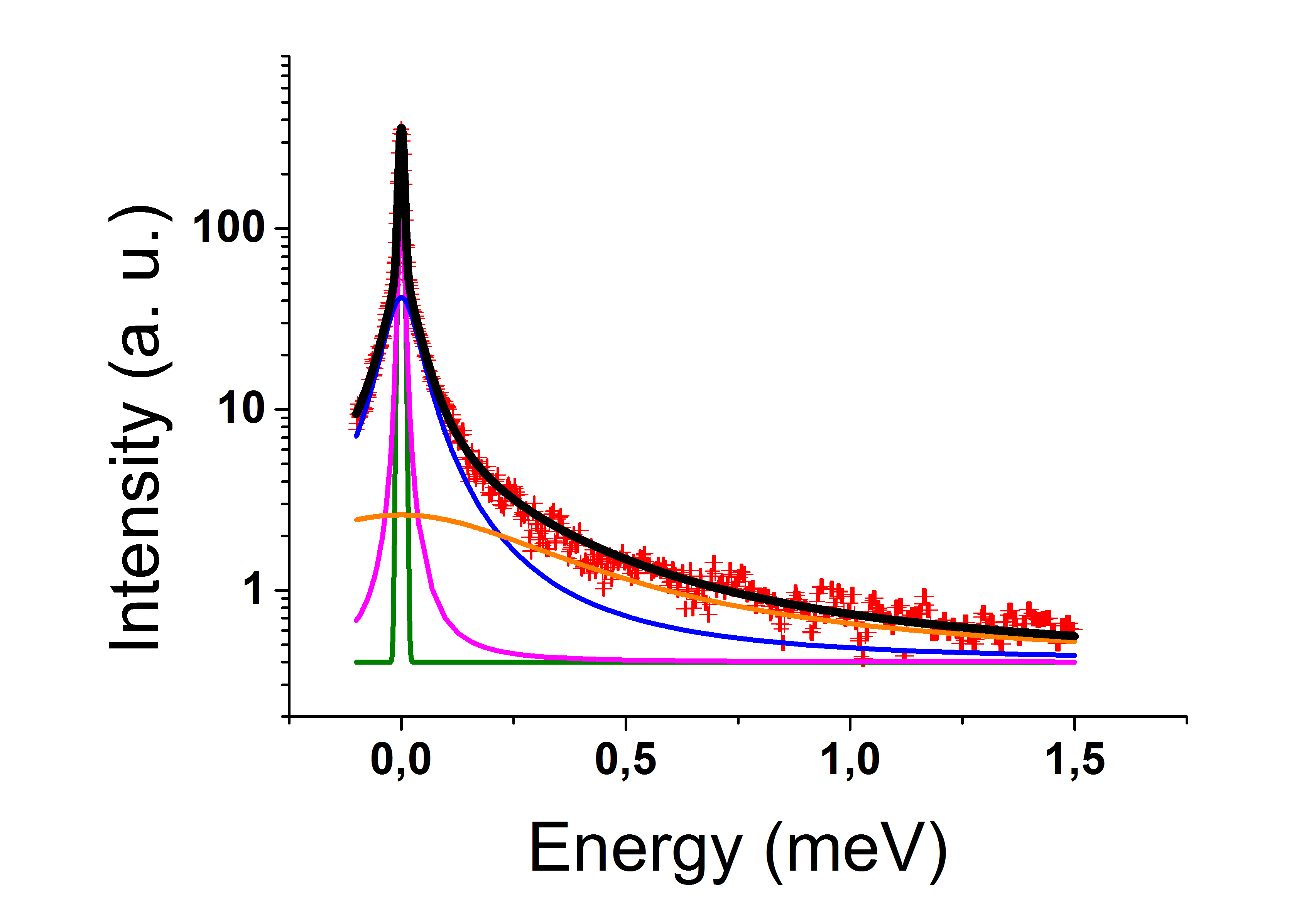 | 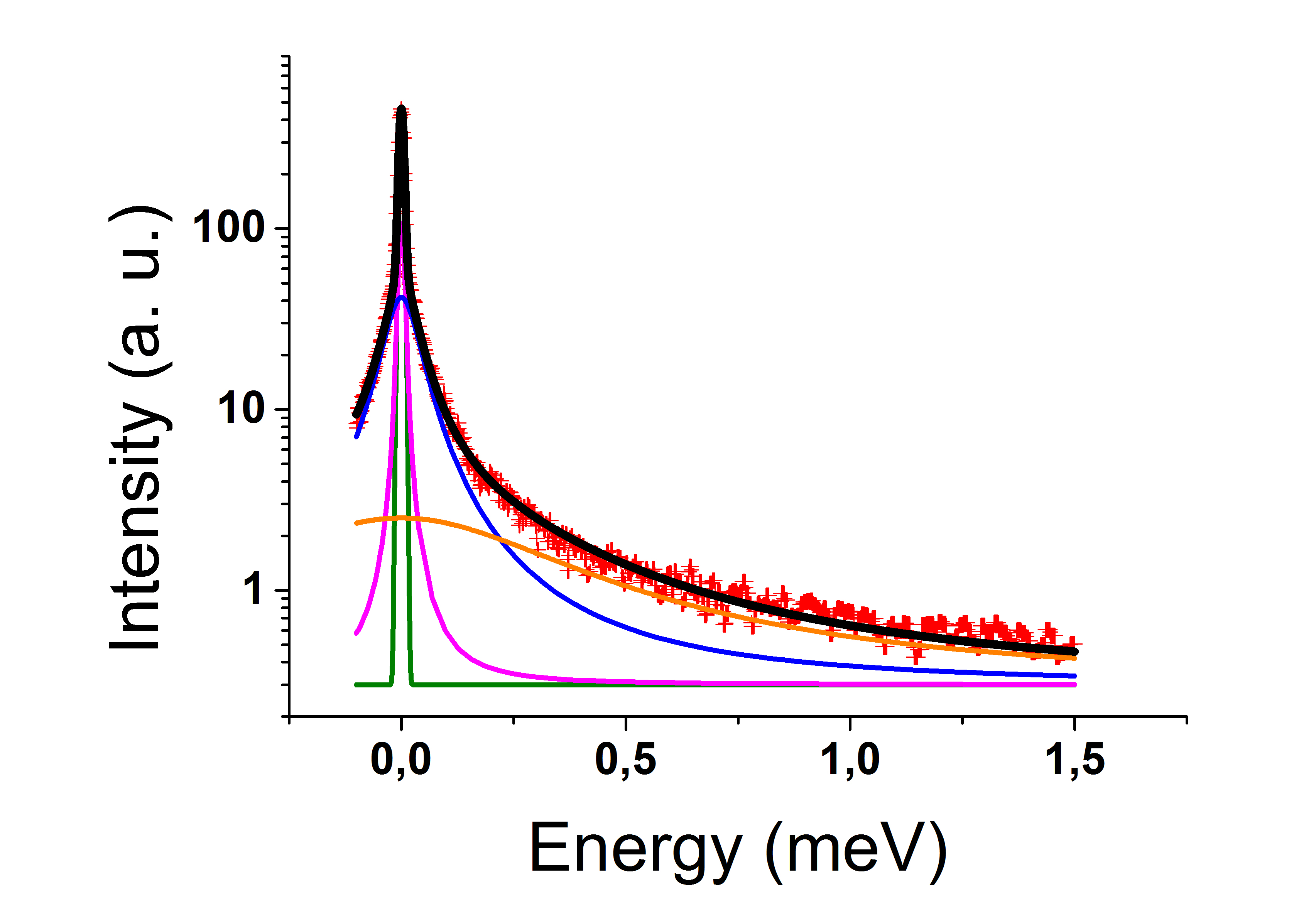 |
|  | 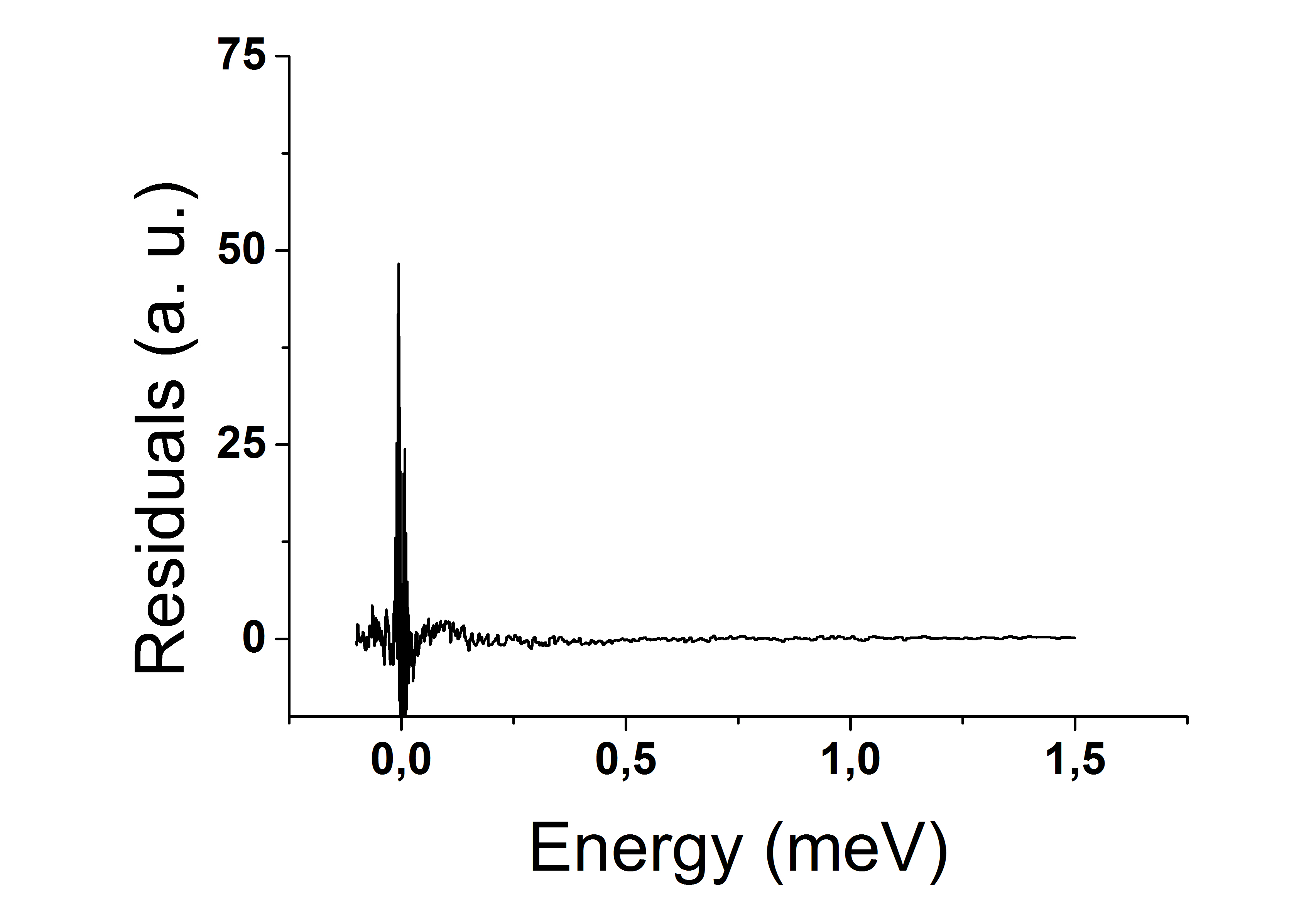 | 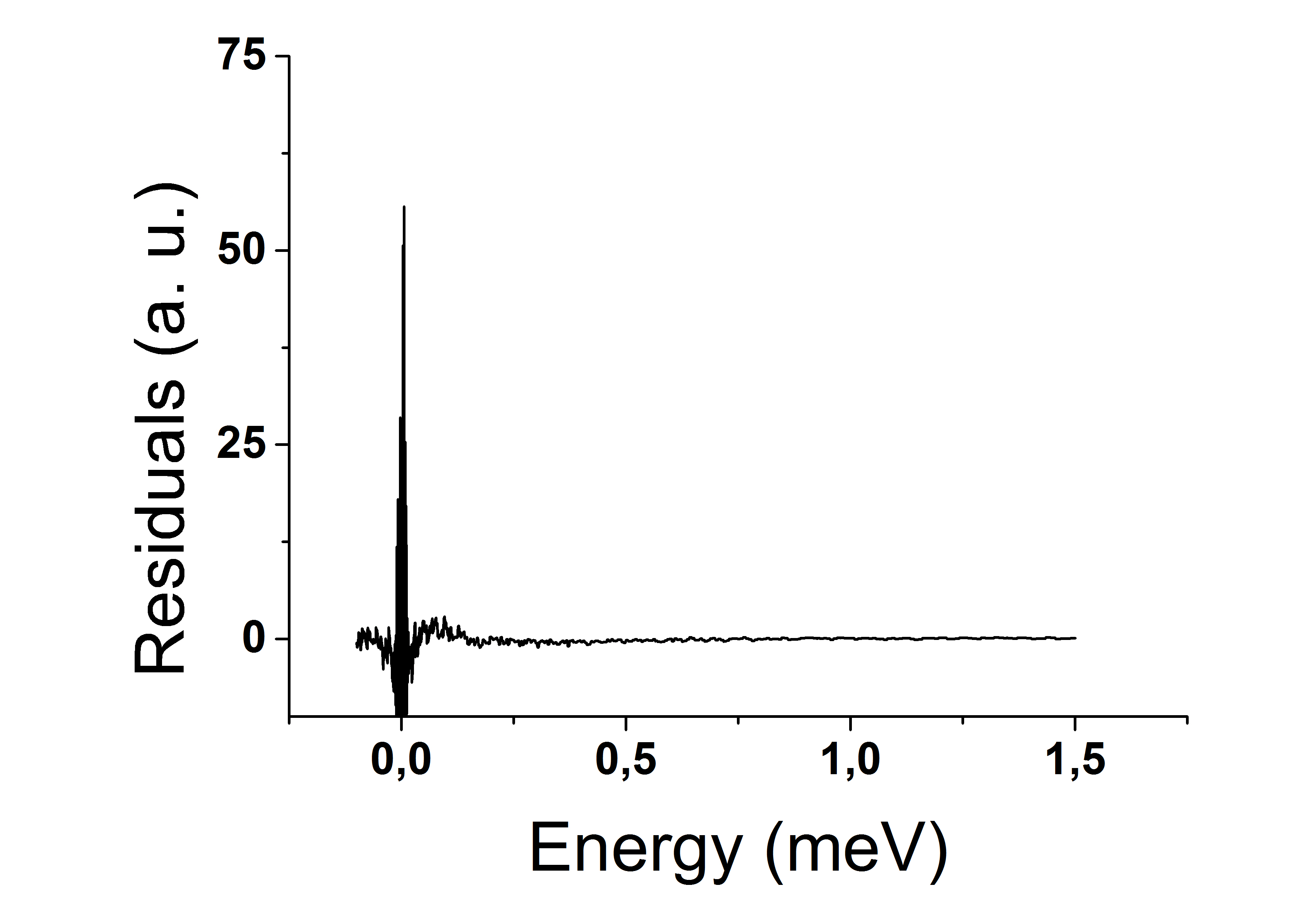 |
| 0.55 | 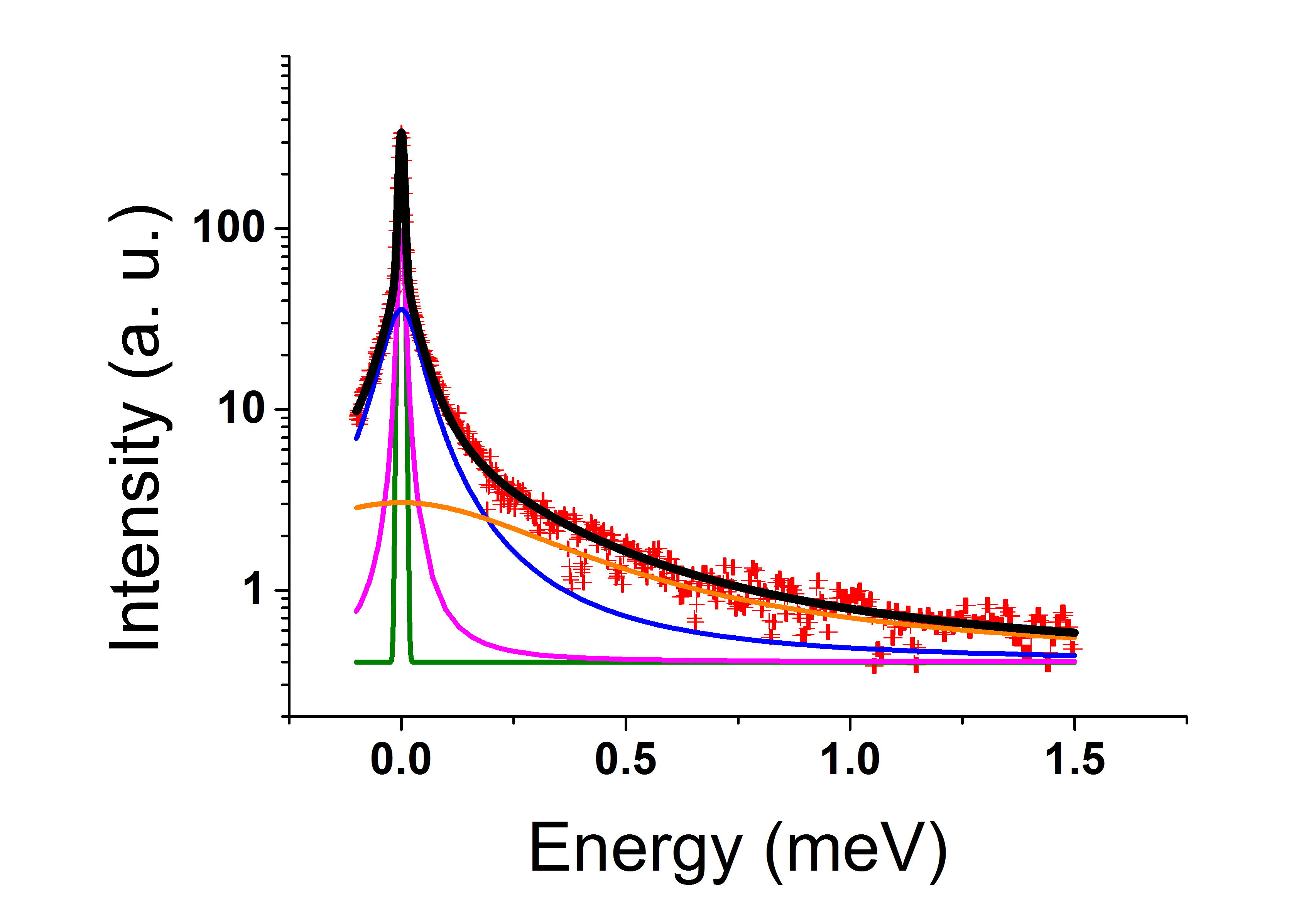 | 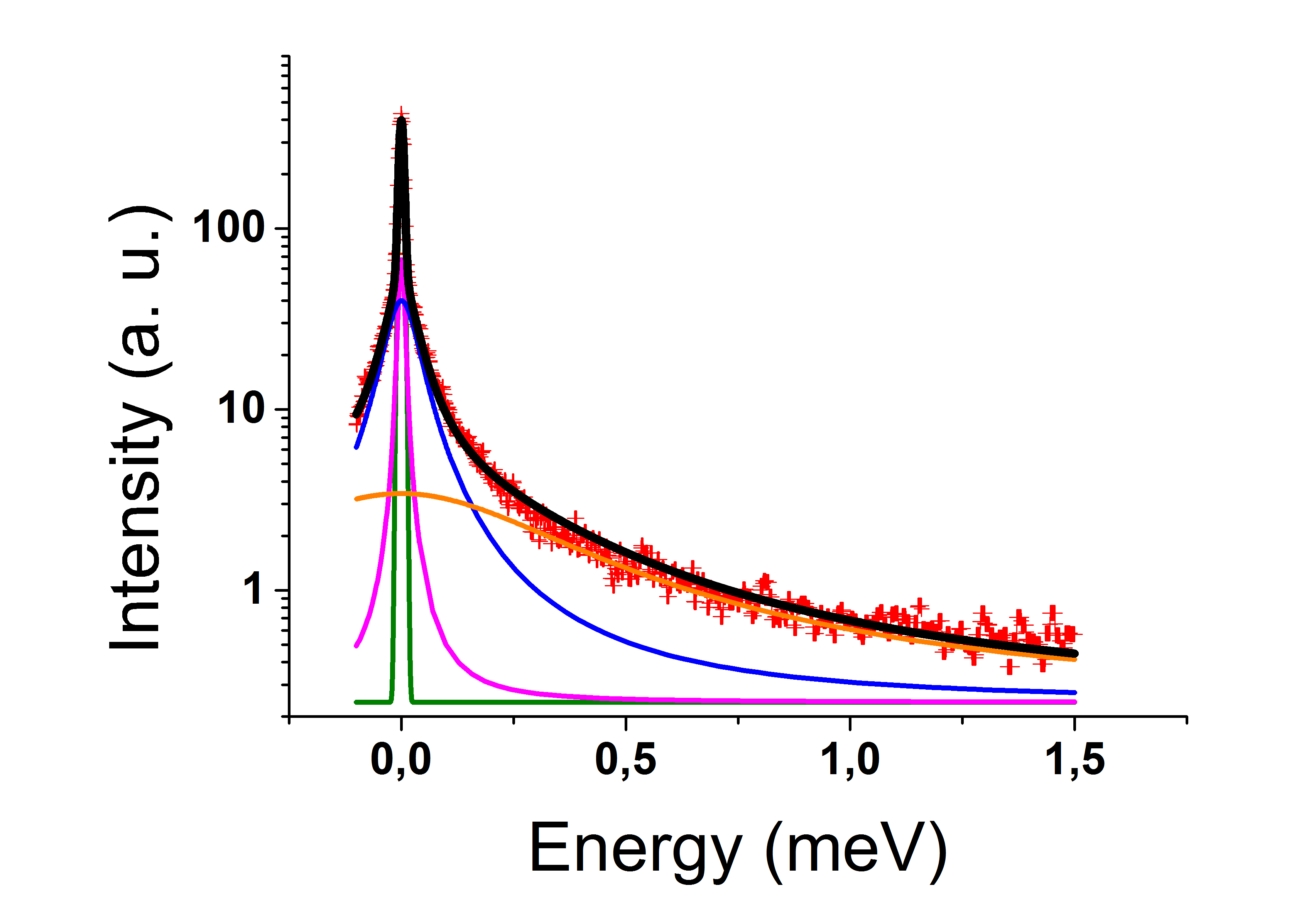 |
|  | 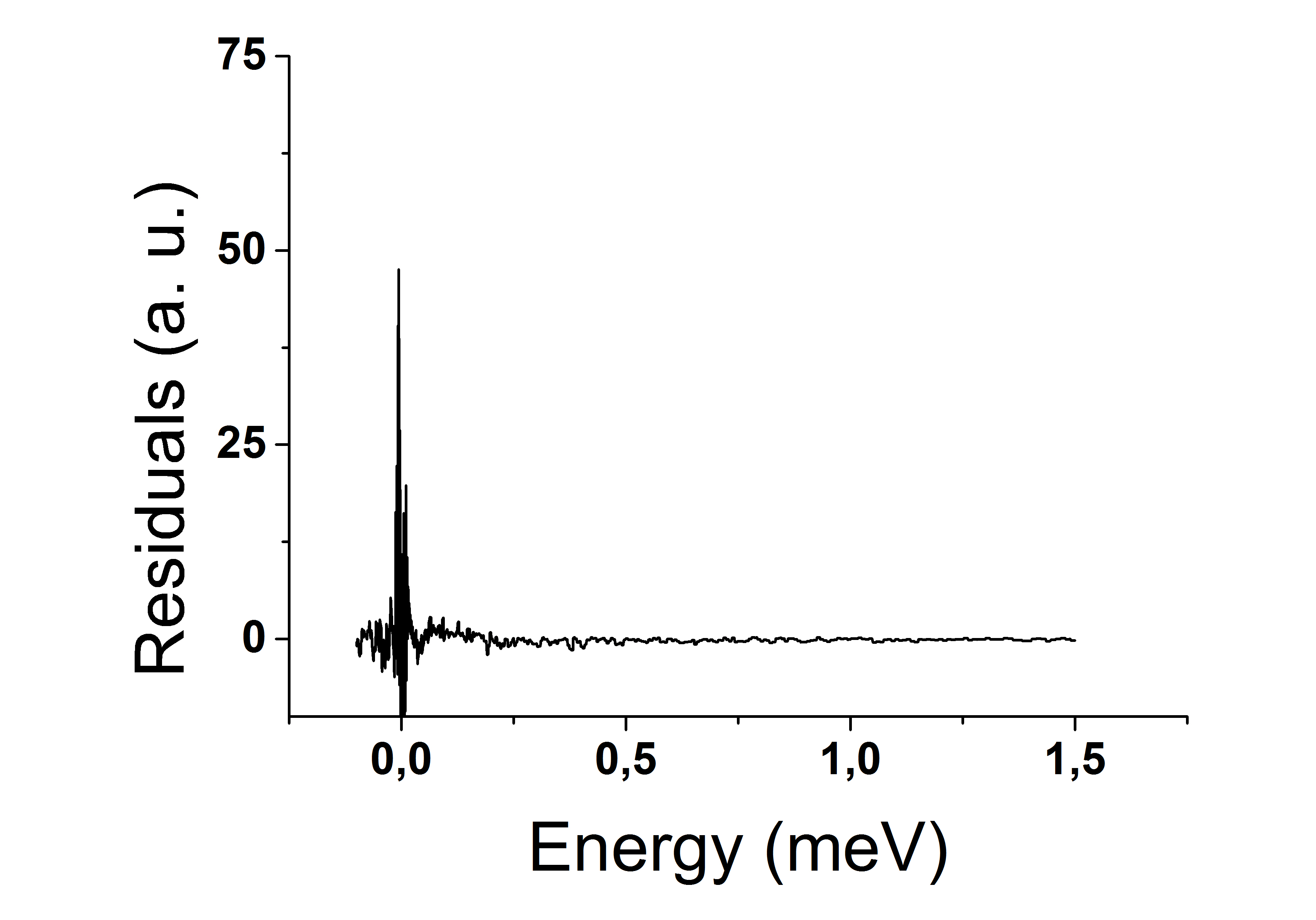 | 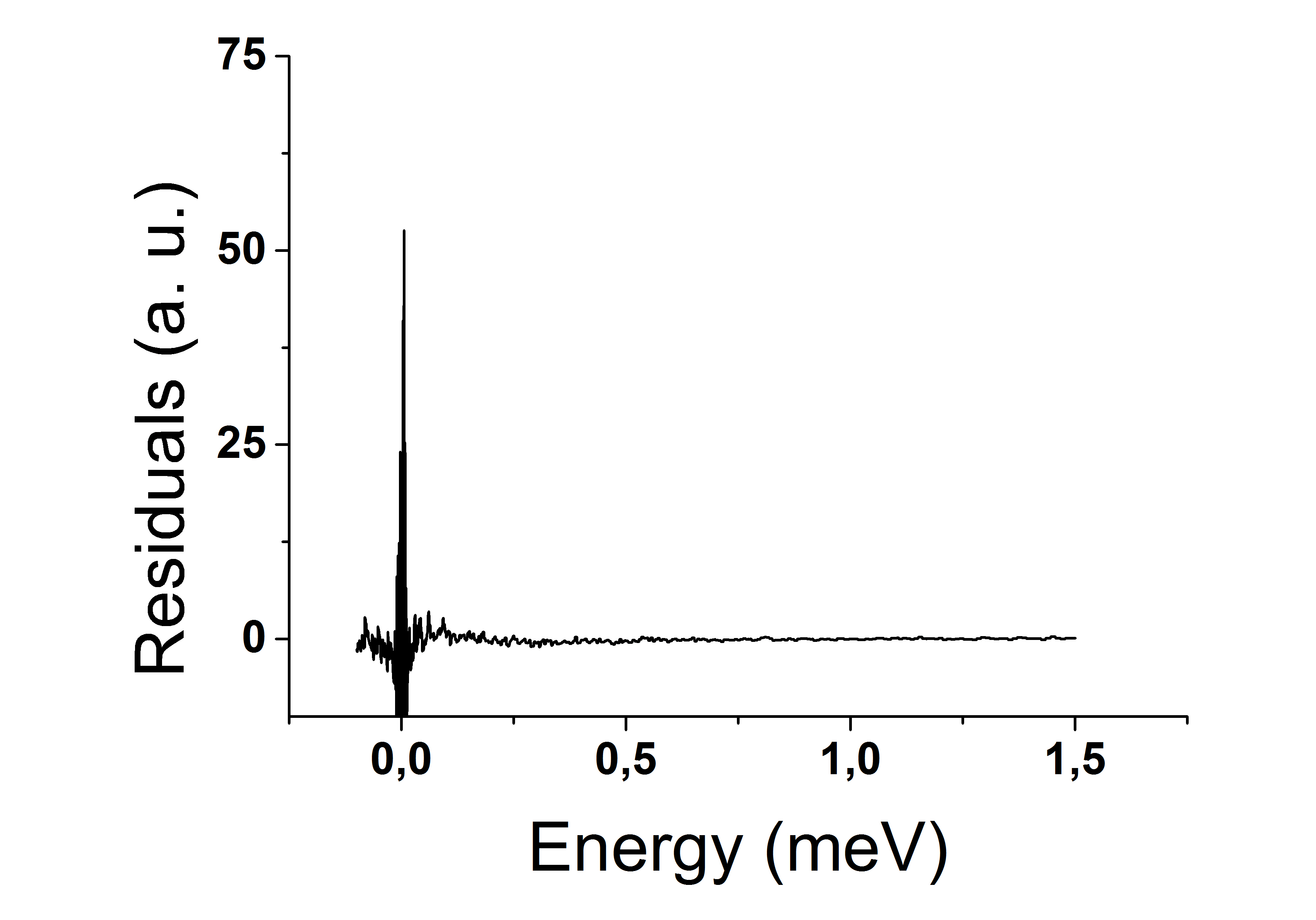 |
| 0.62 | 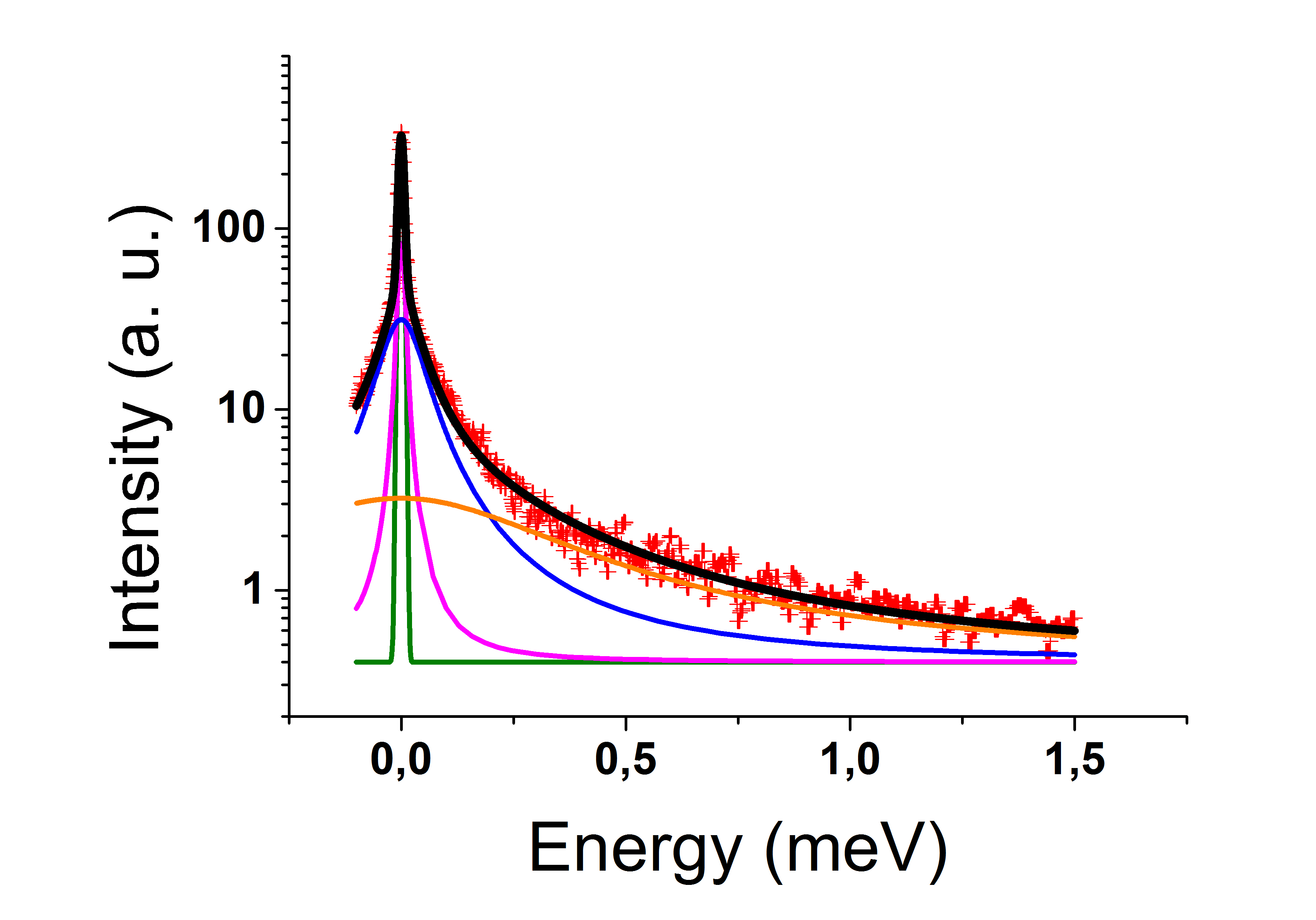 | 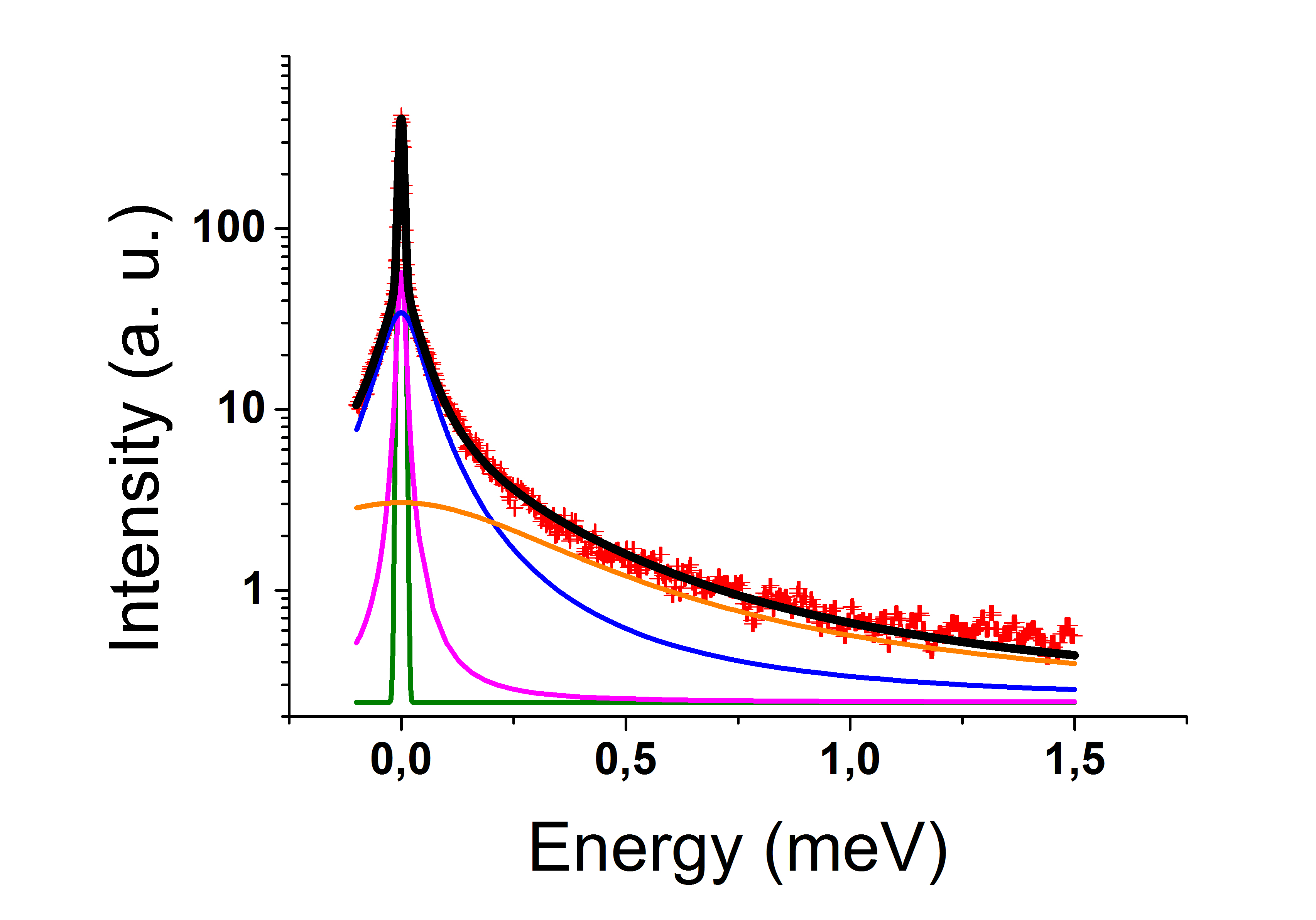 |
|  | 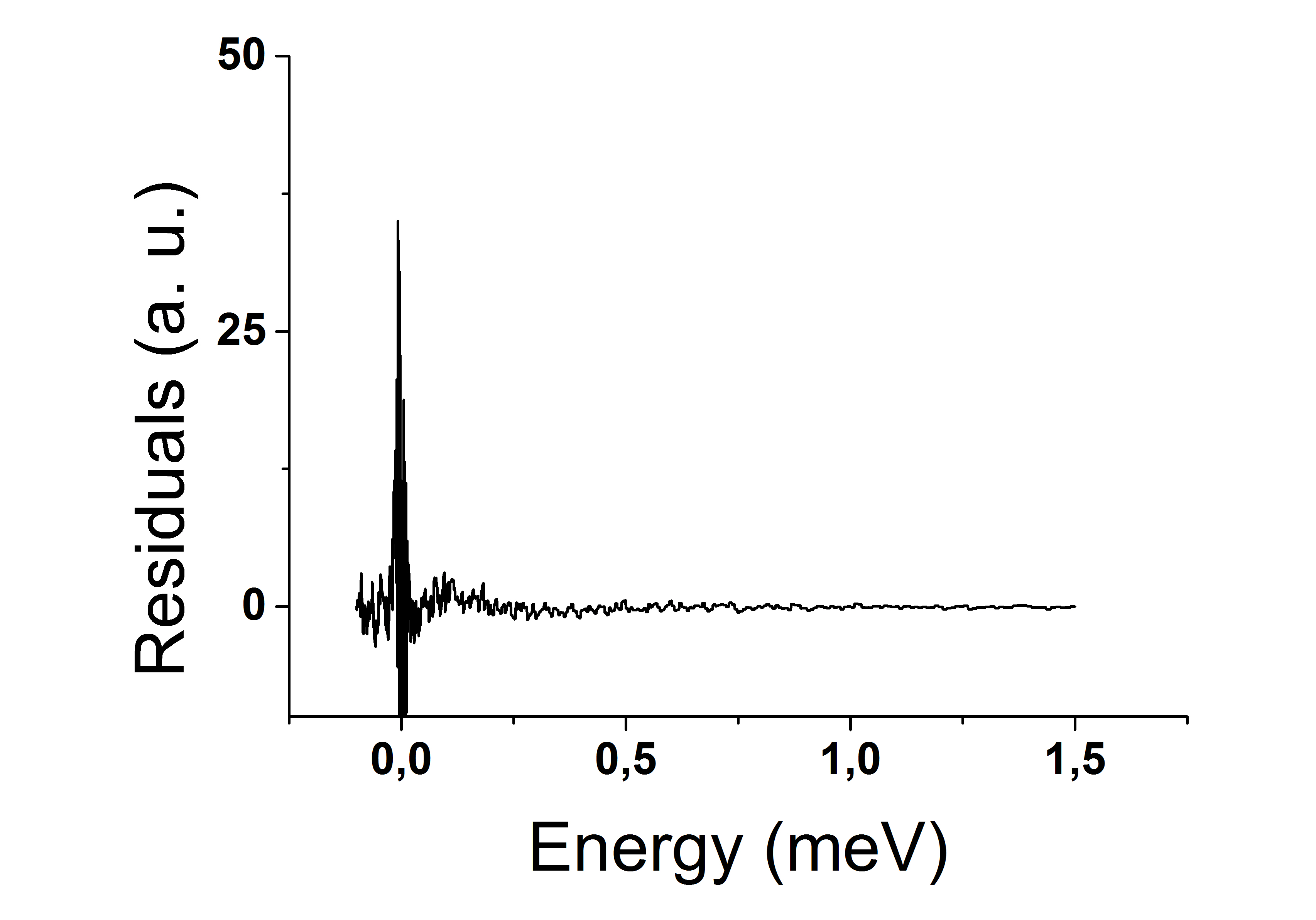 | 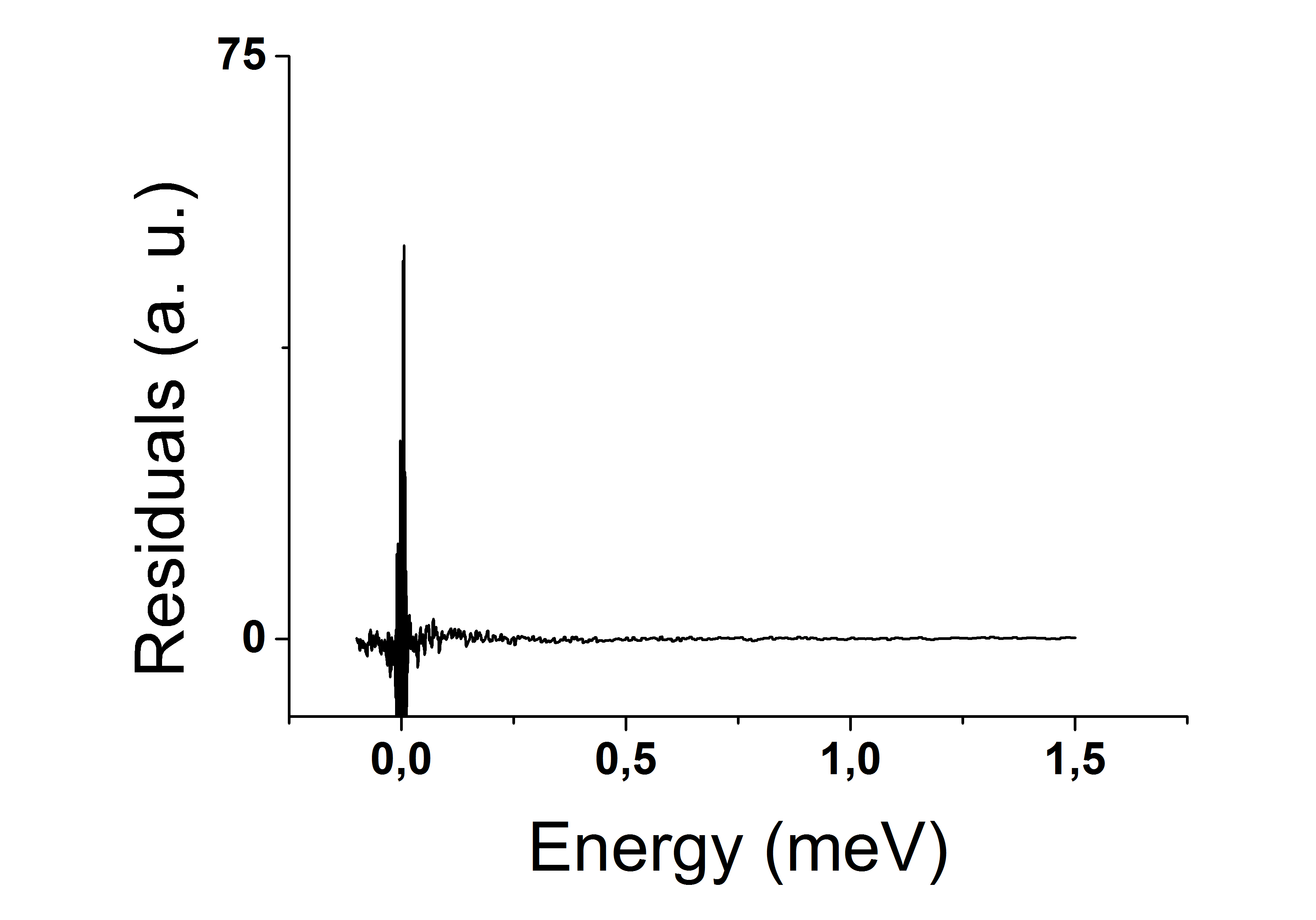 |
| 0.68 | 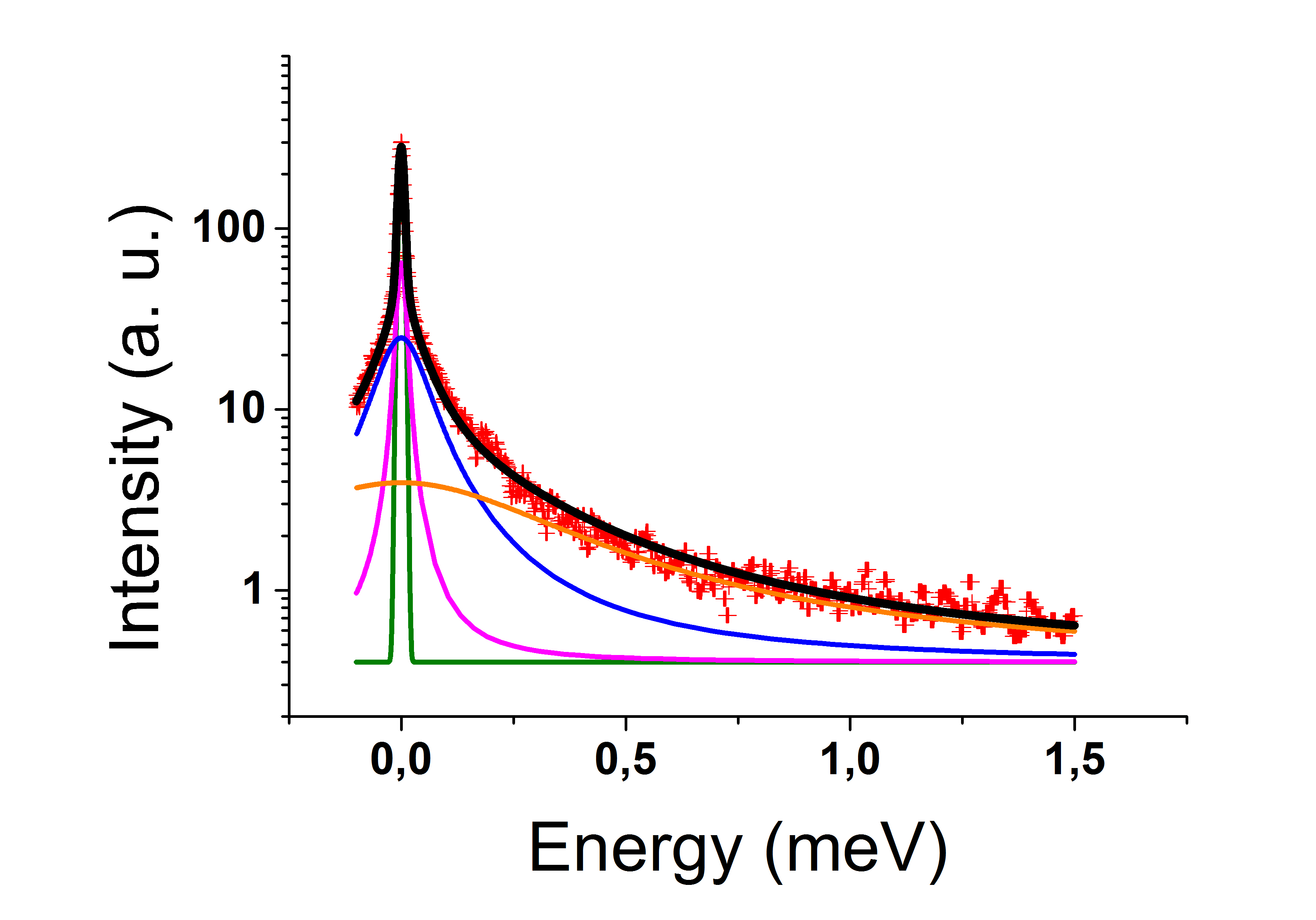 | 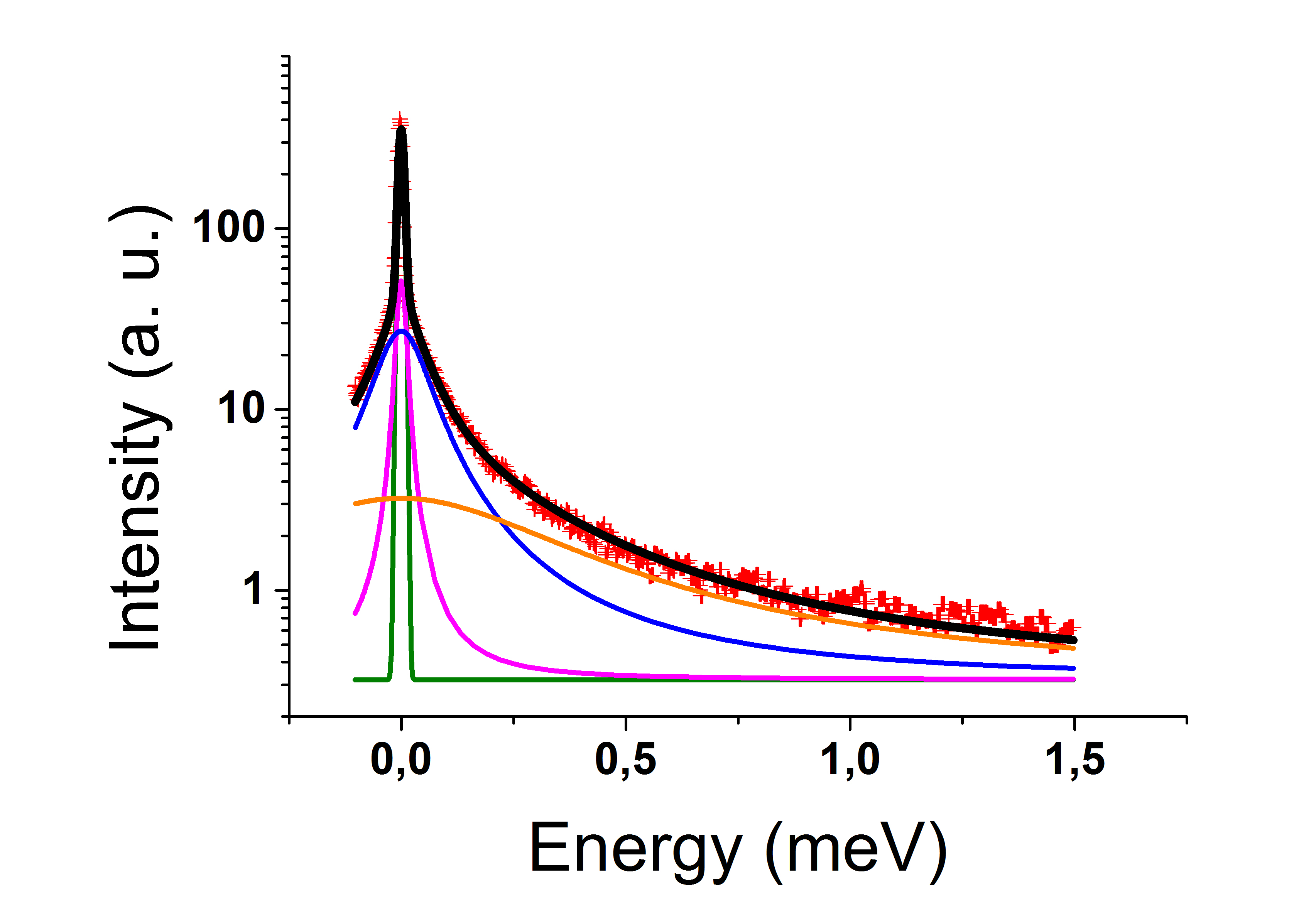 |
|  | 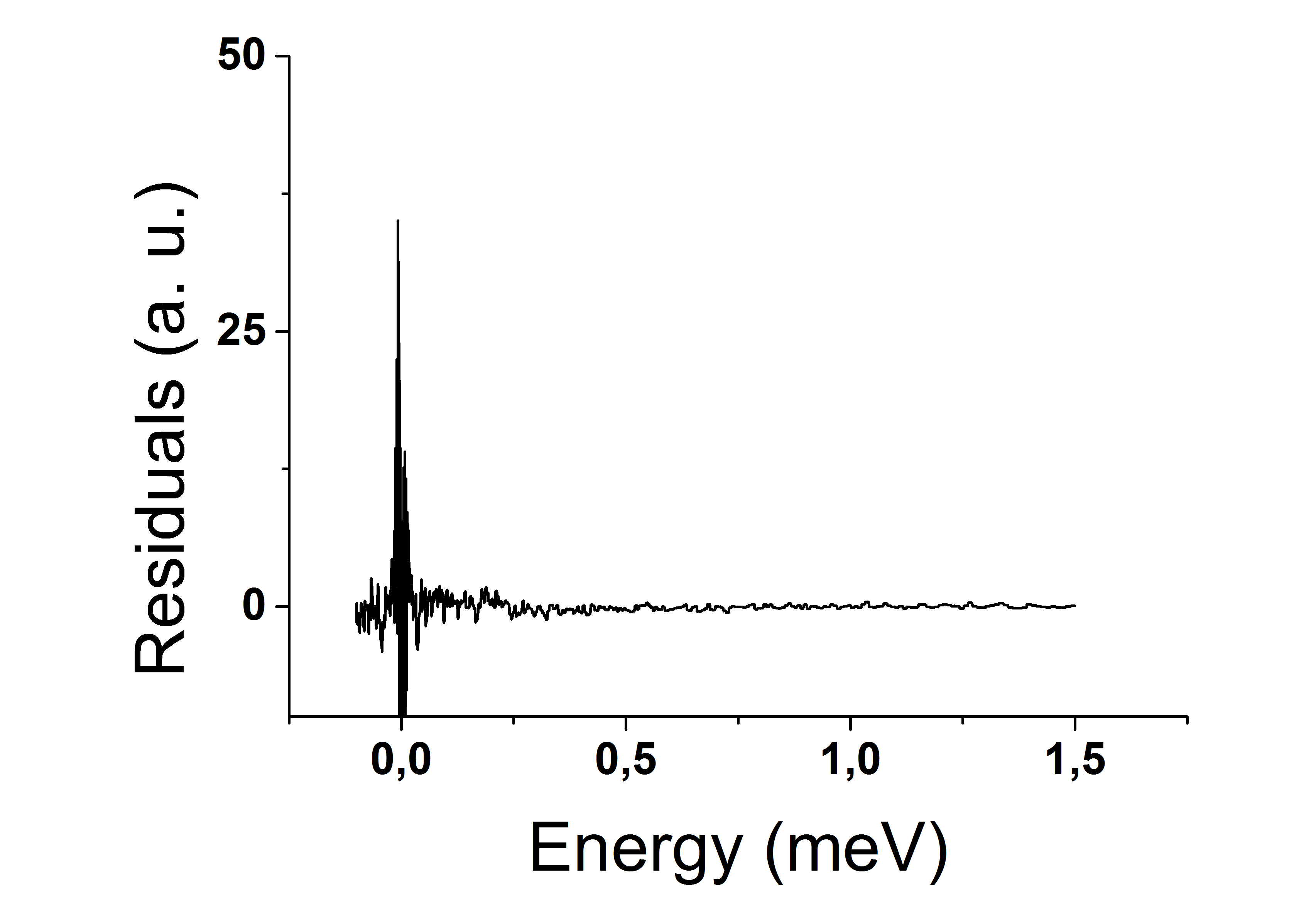 | 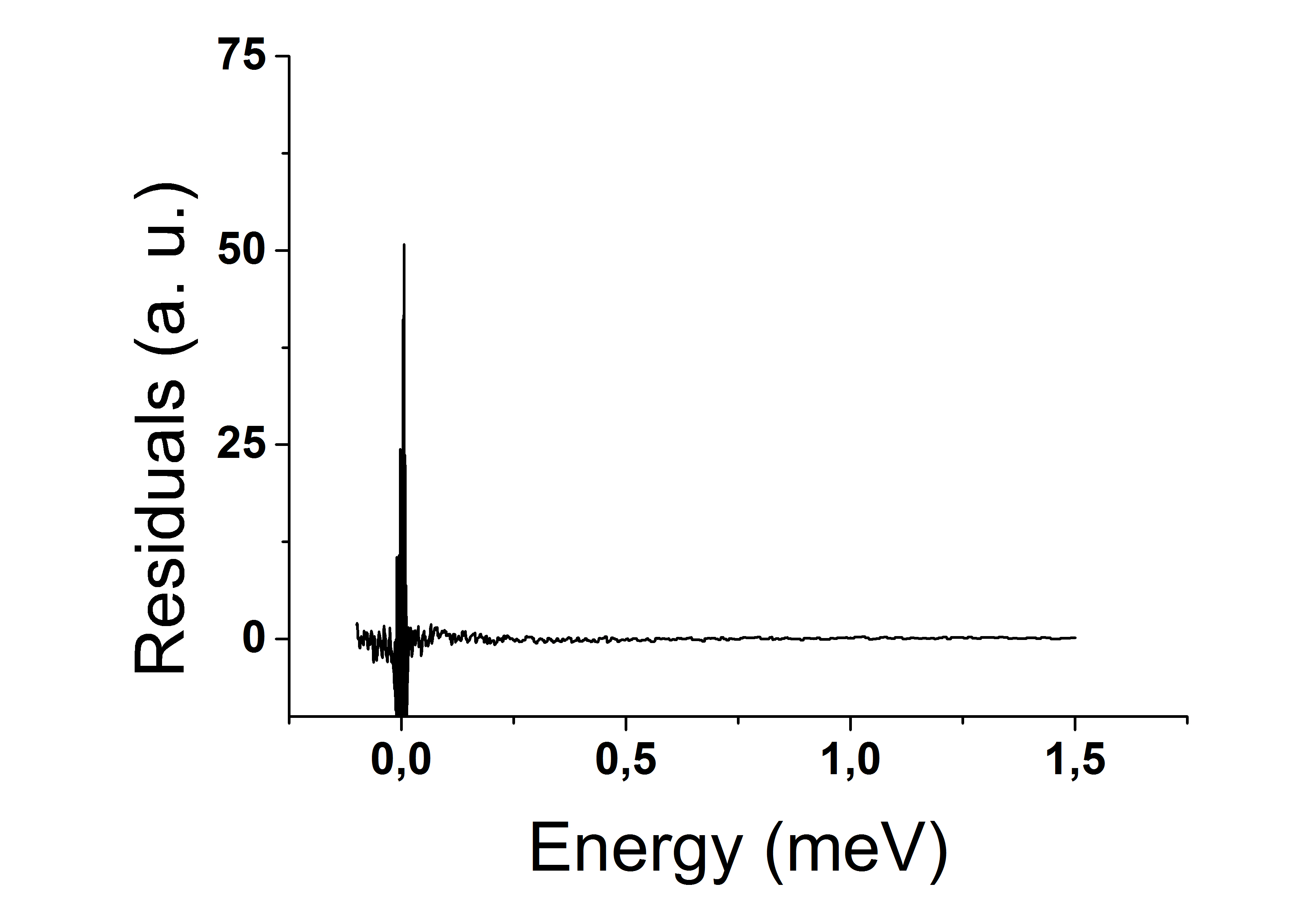 |
| 0.75 | 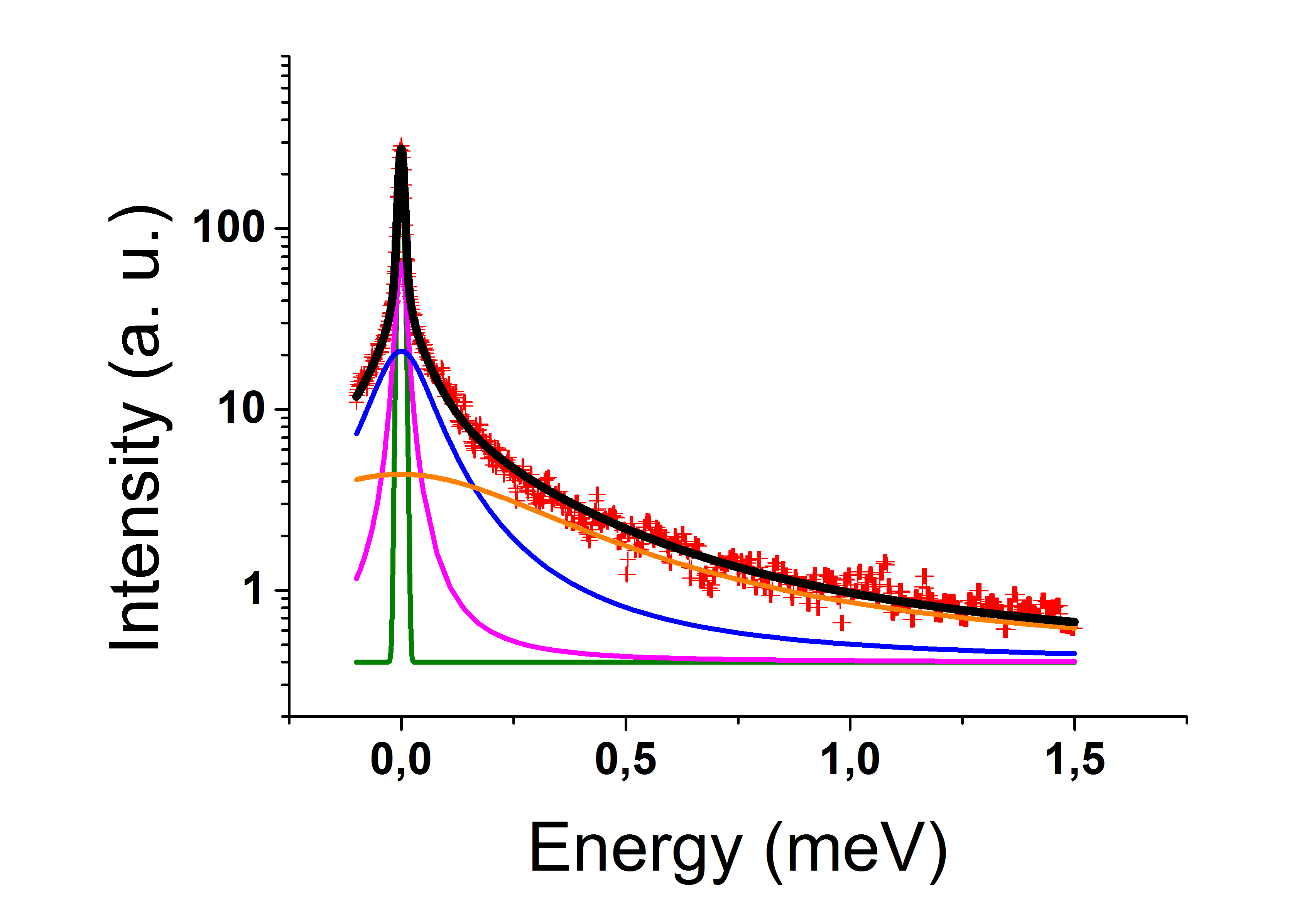 | 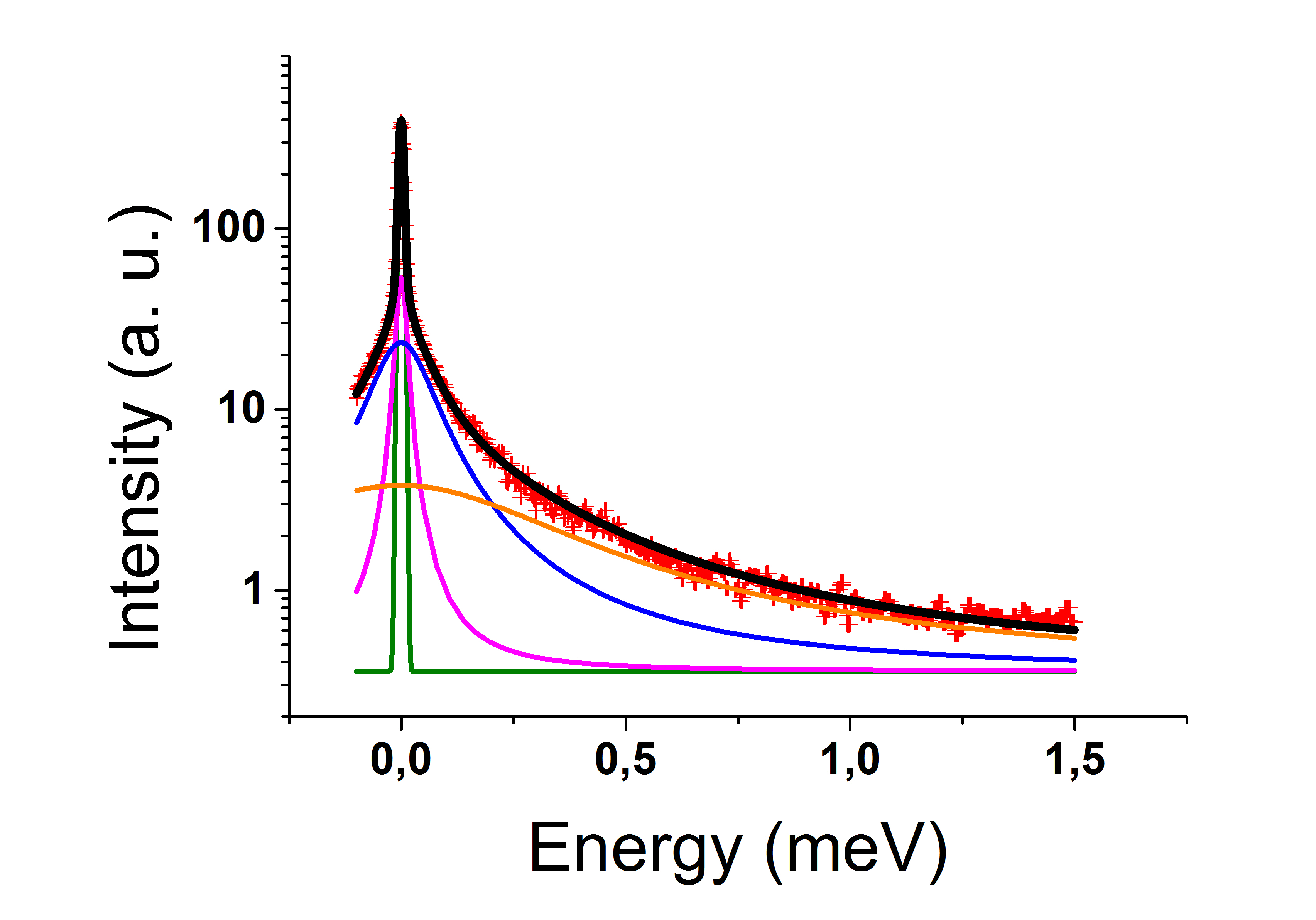 |
|  | 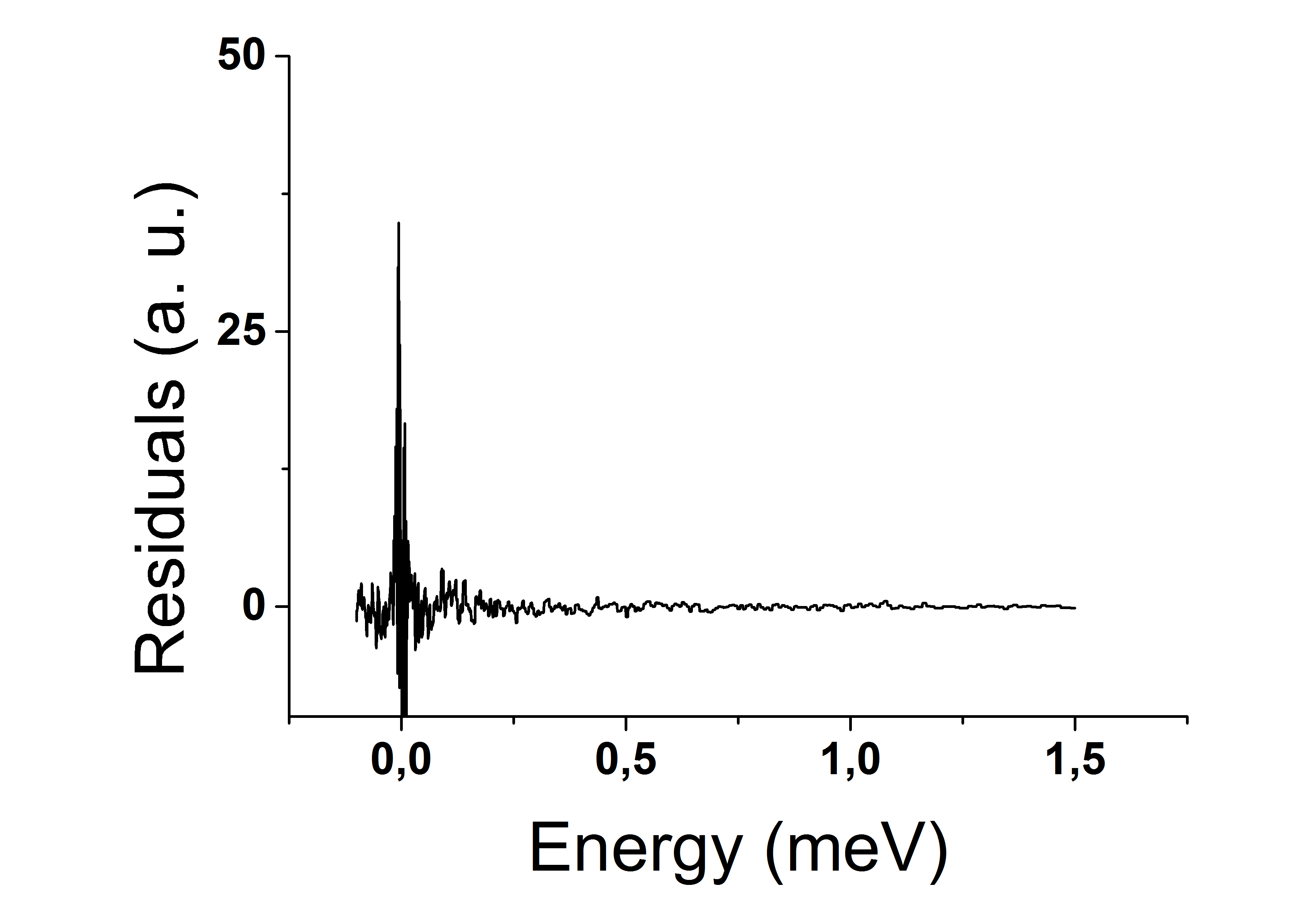 | 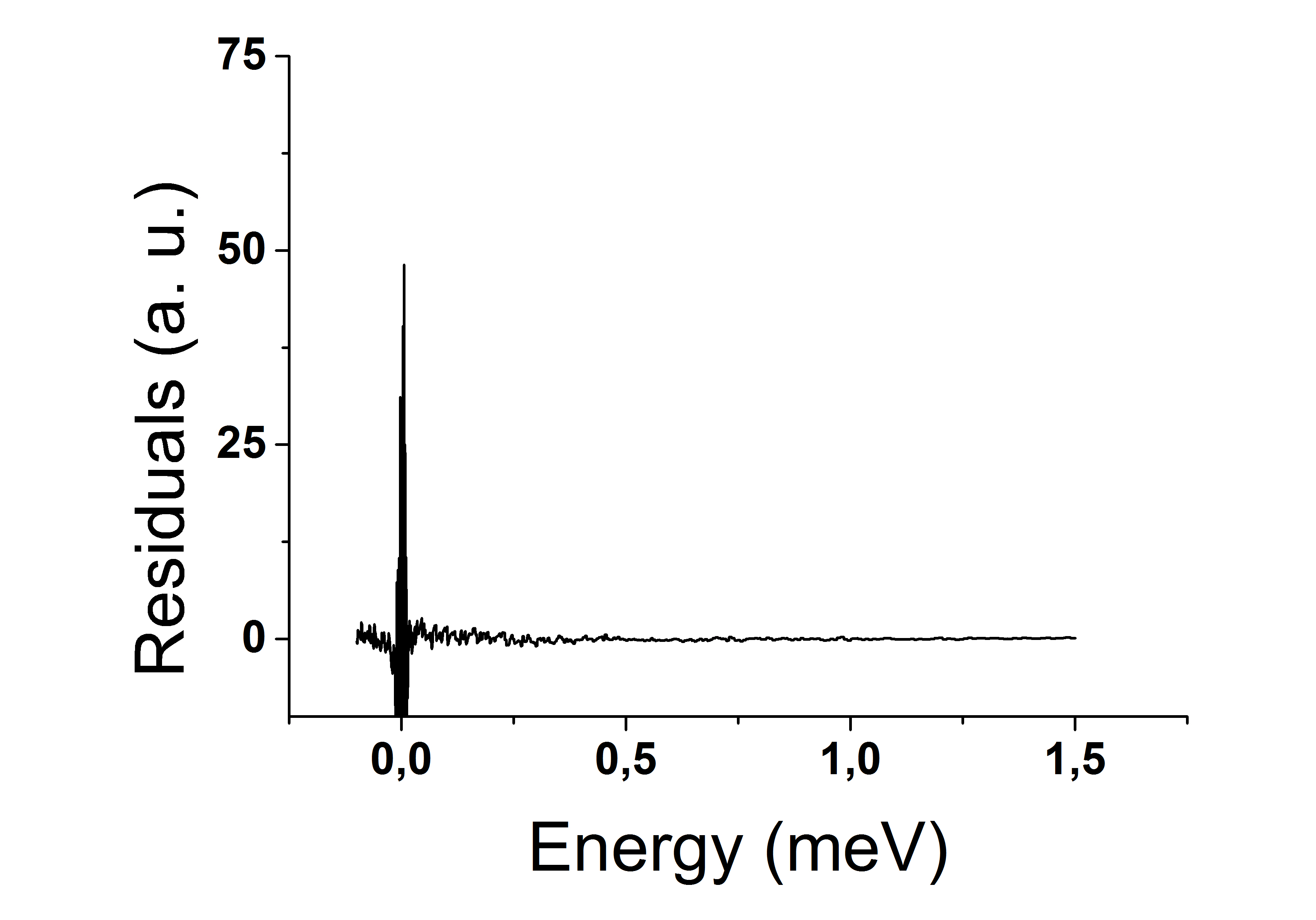 |
| 0.8 | 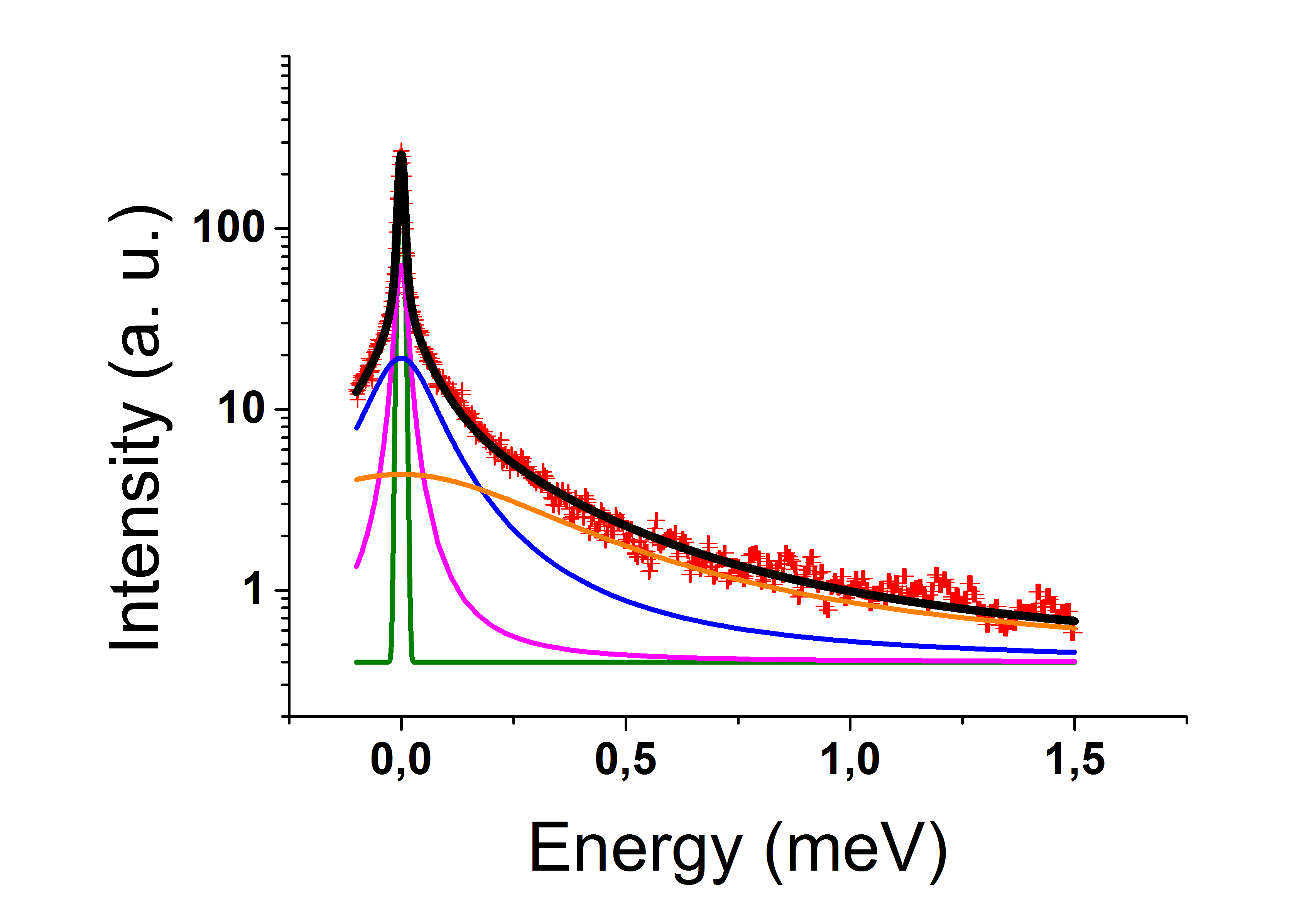 | 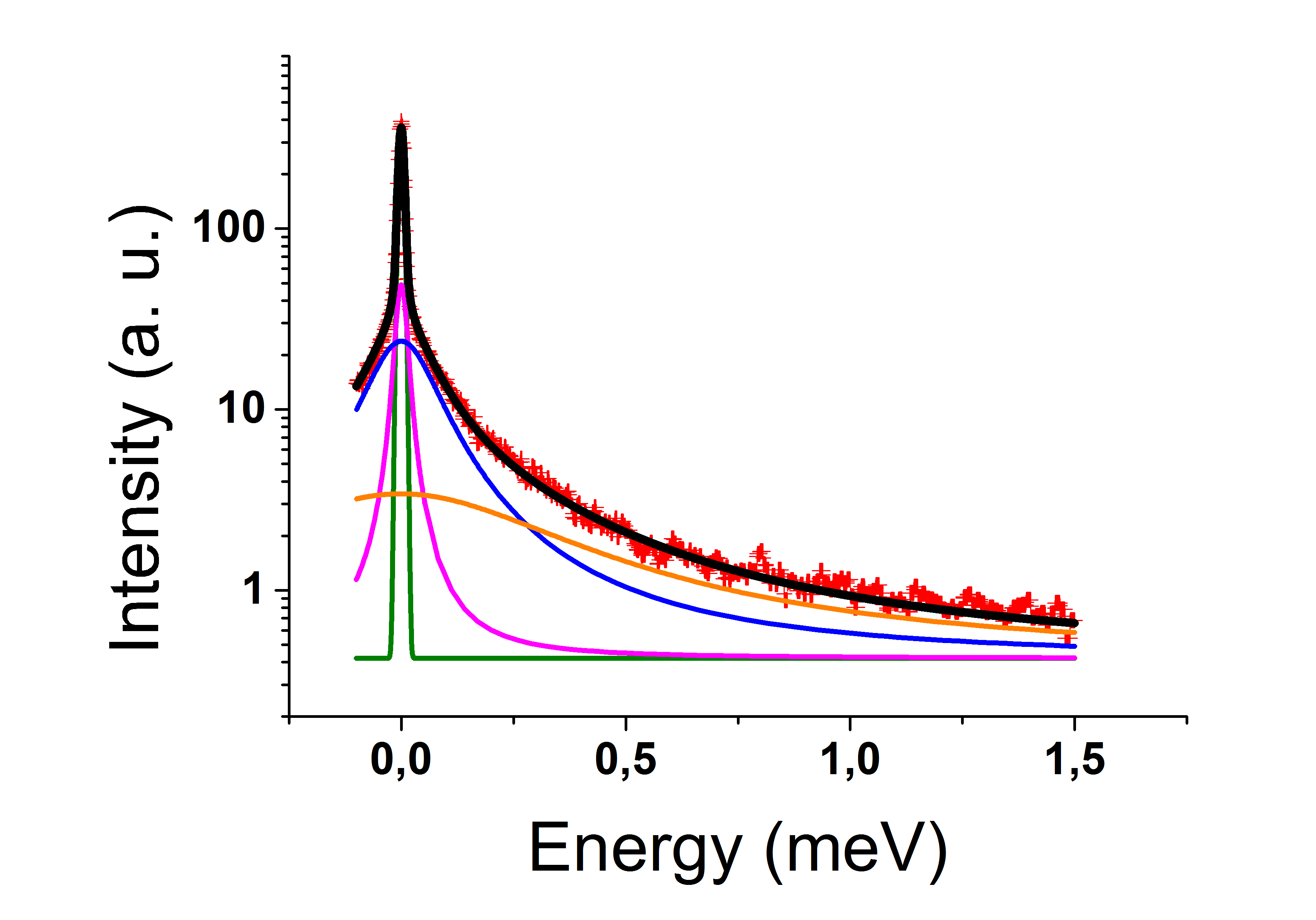 |
|  | 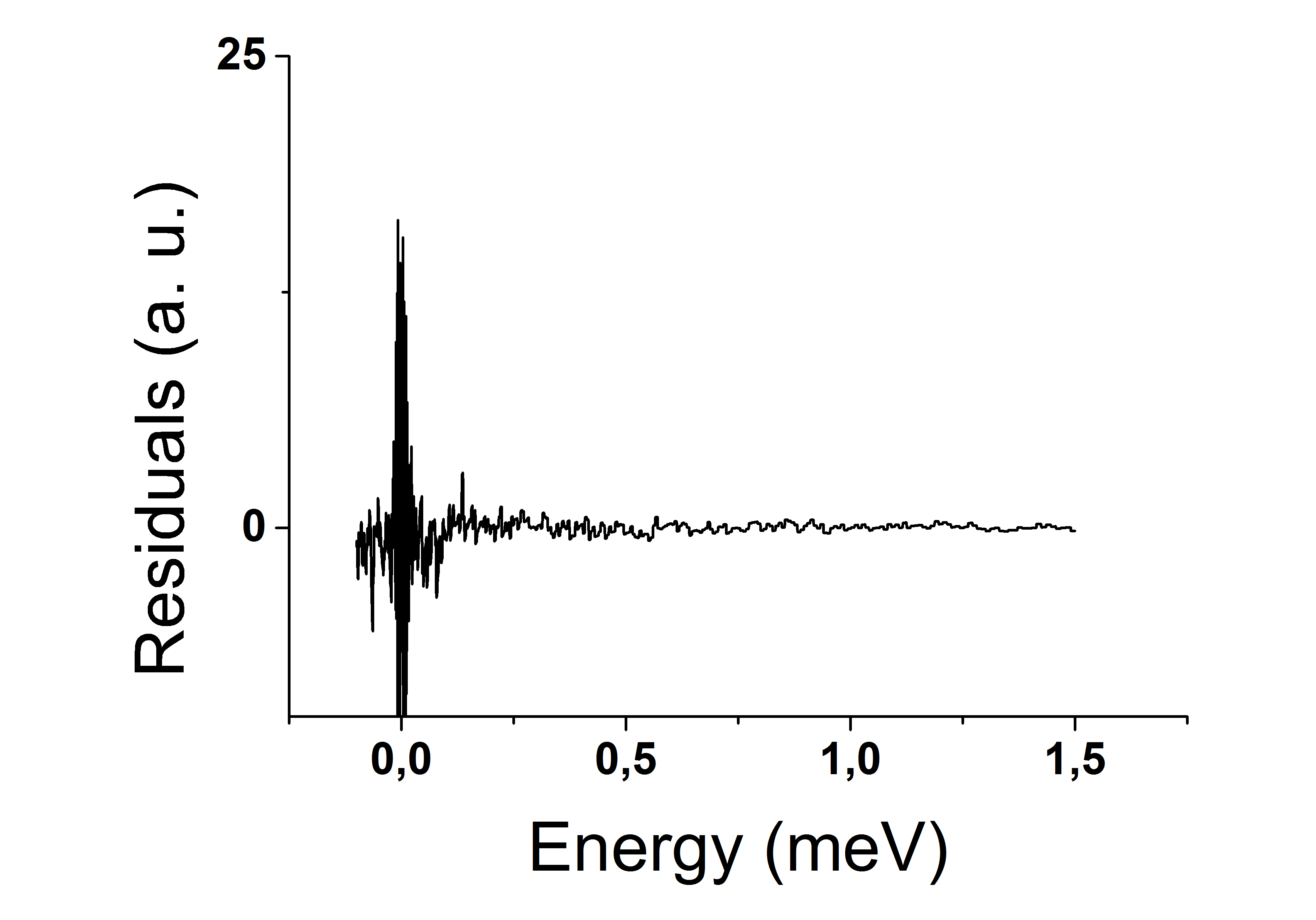 | 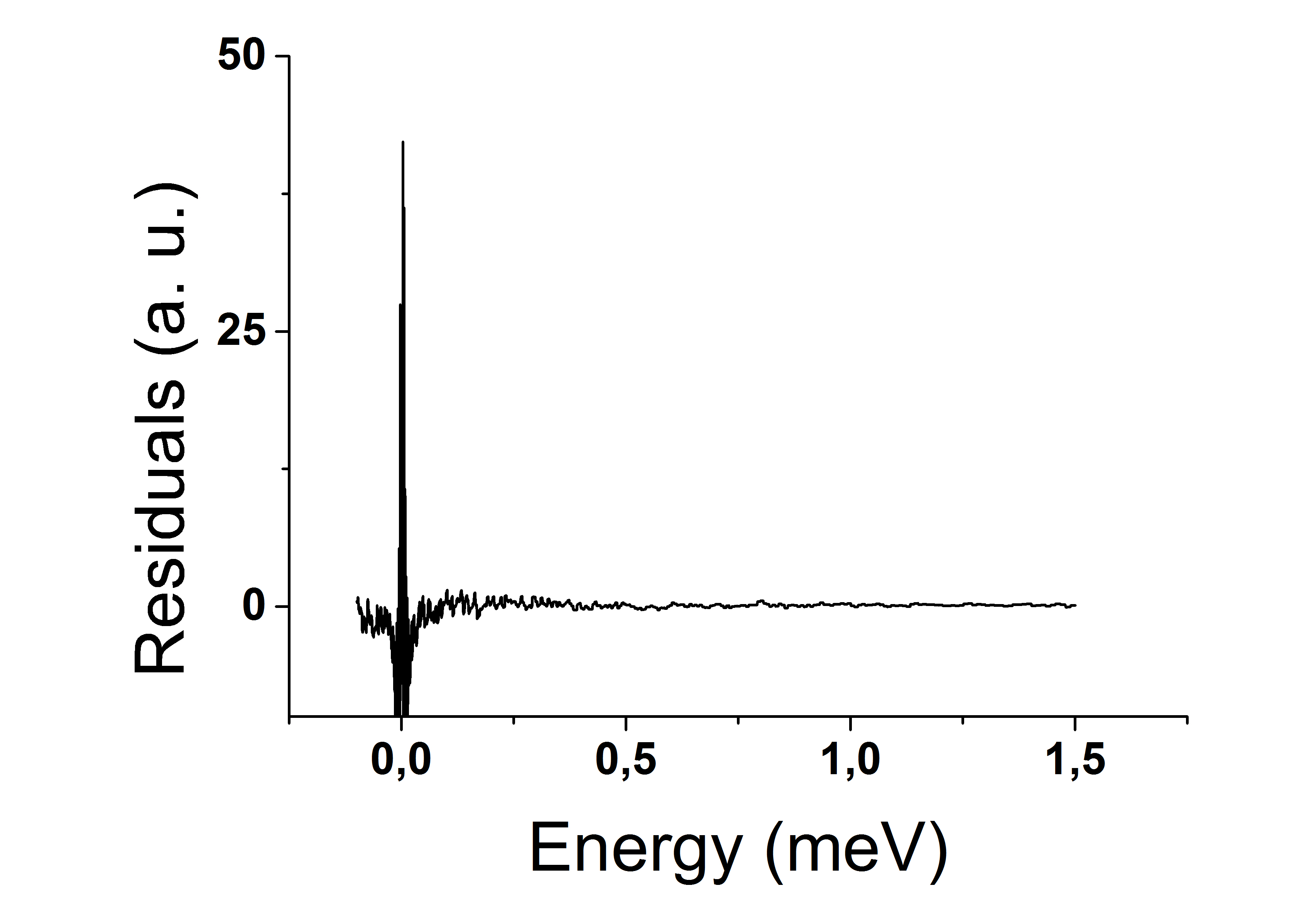 |
| 0.86 | 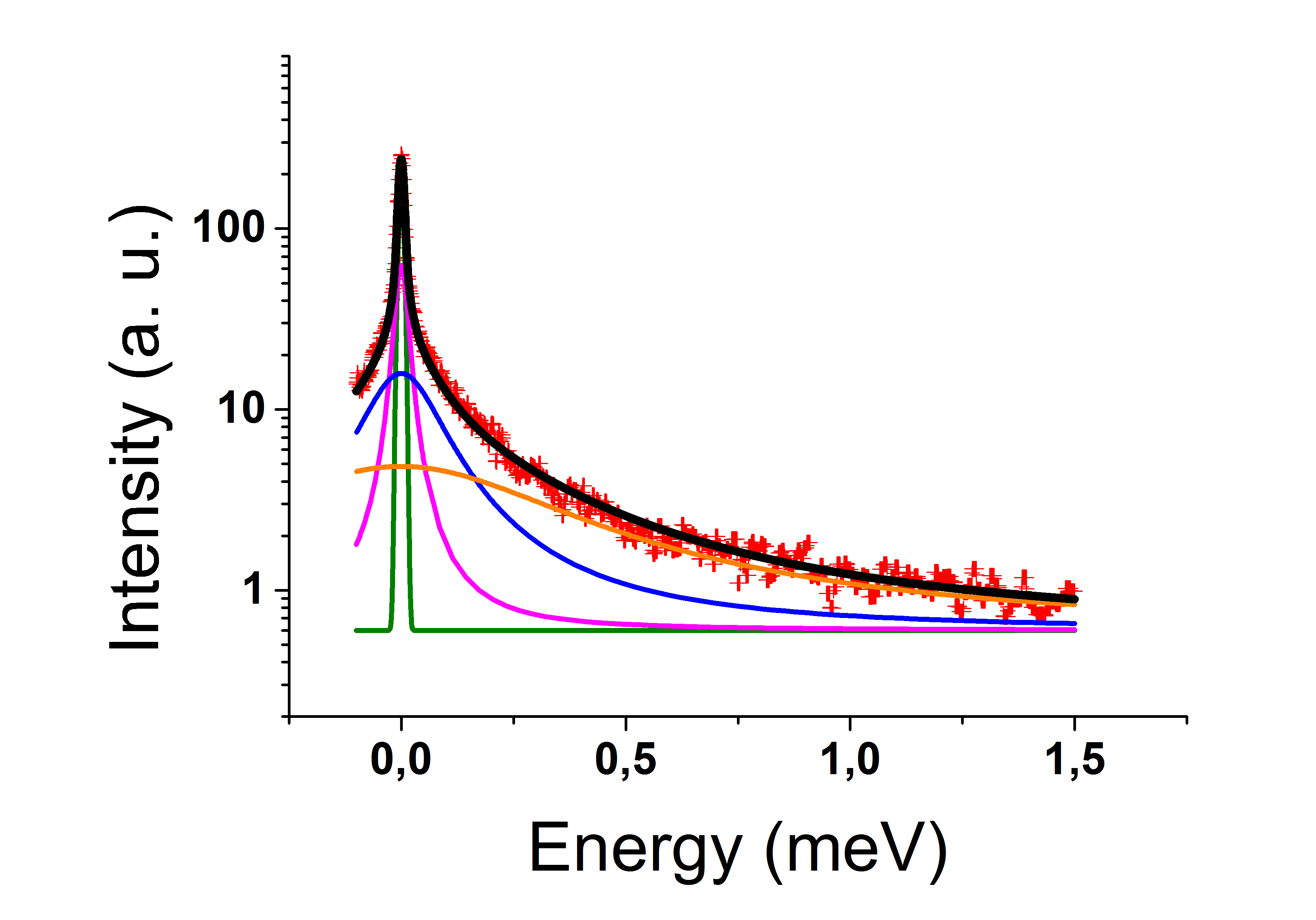 | 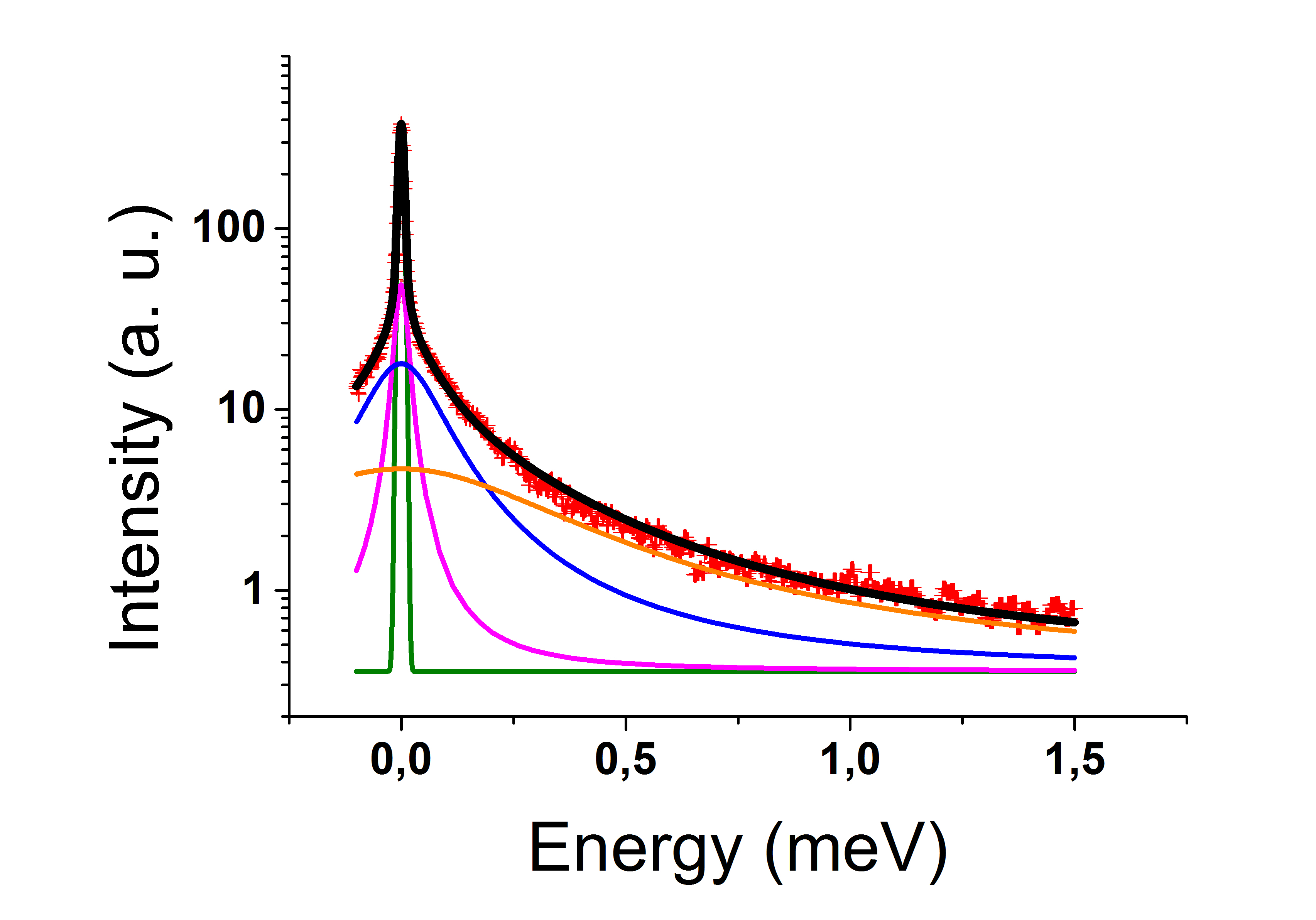 |
|  | 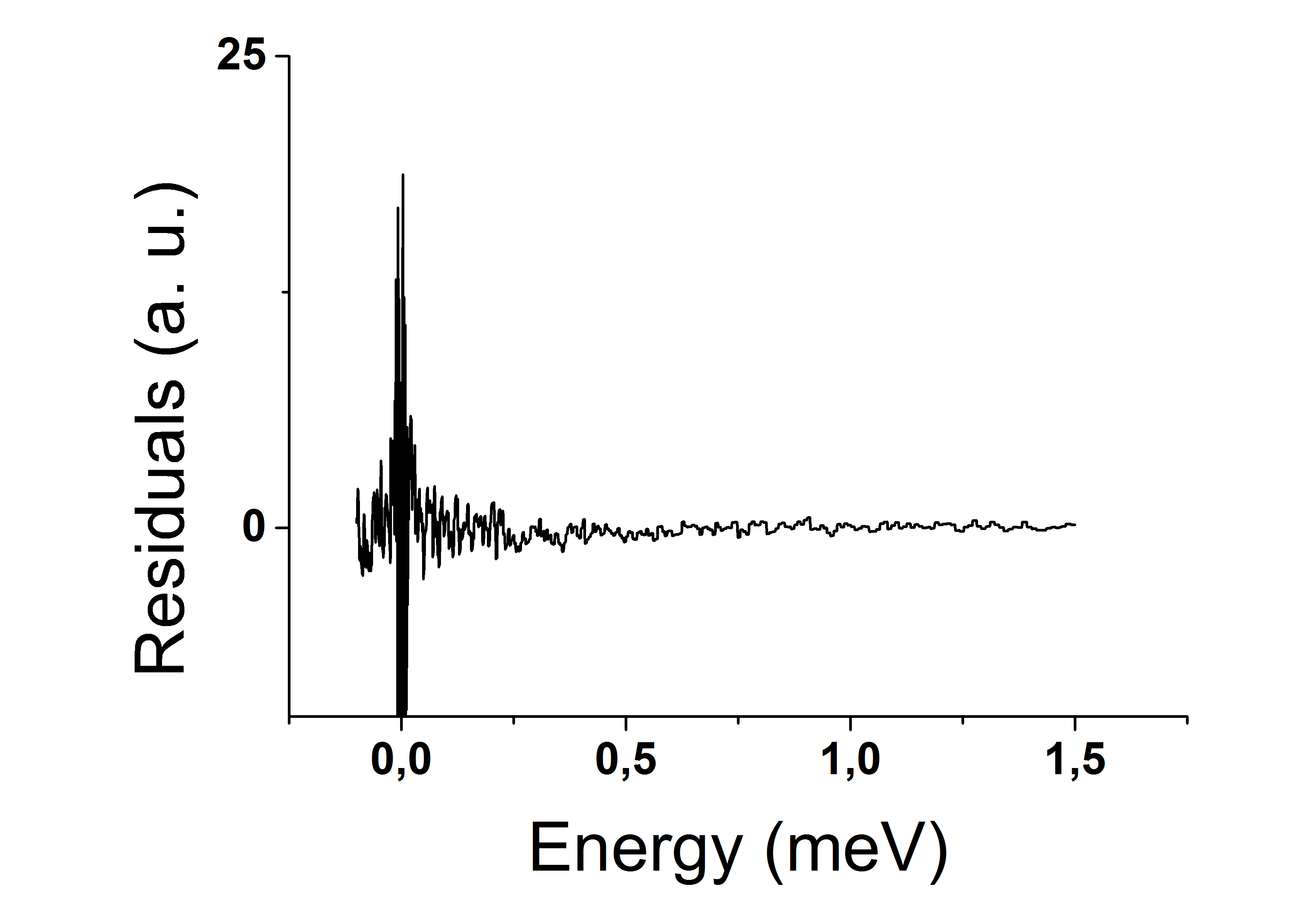 | 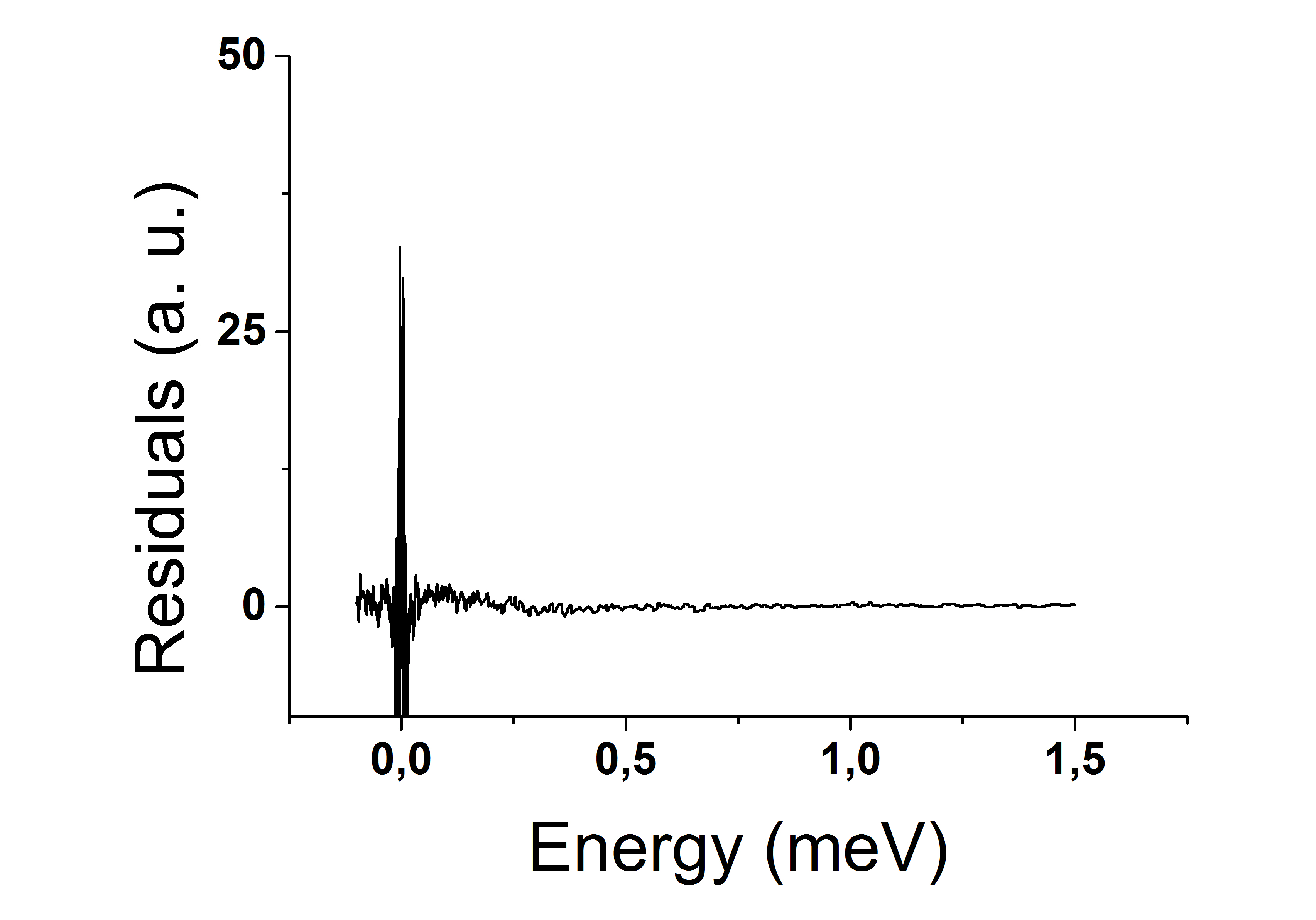 |
| 0.91 | 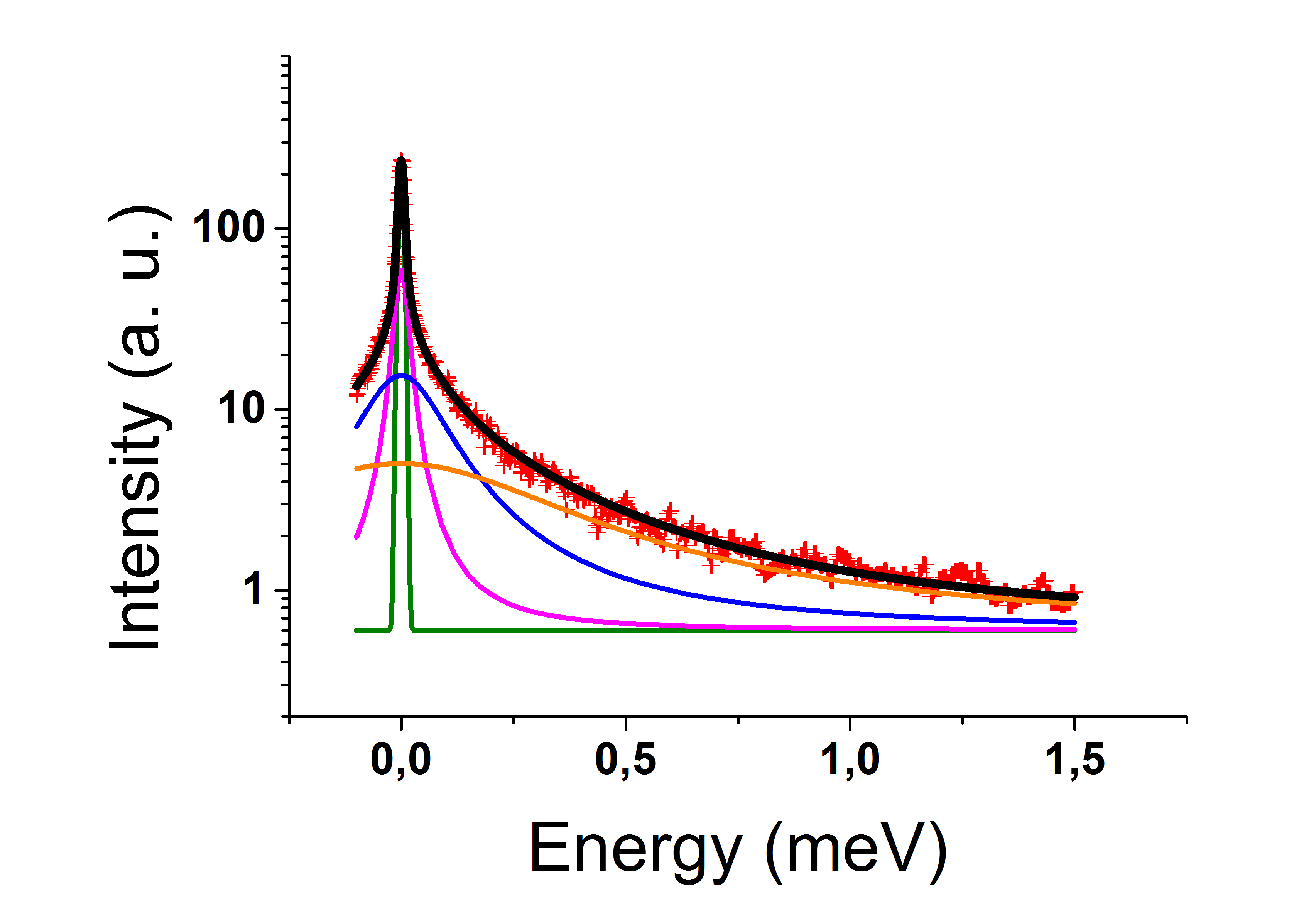 | 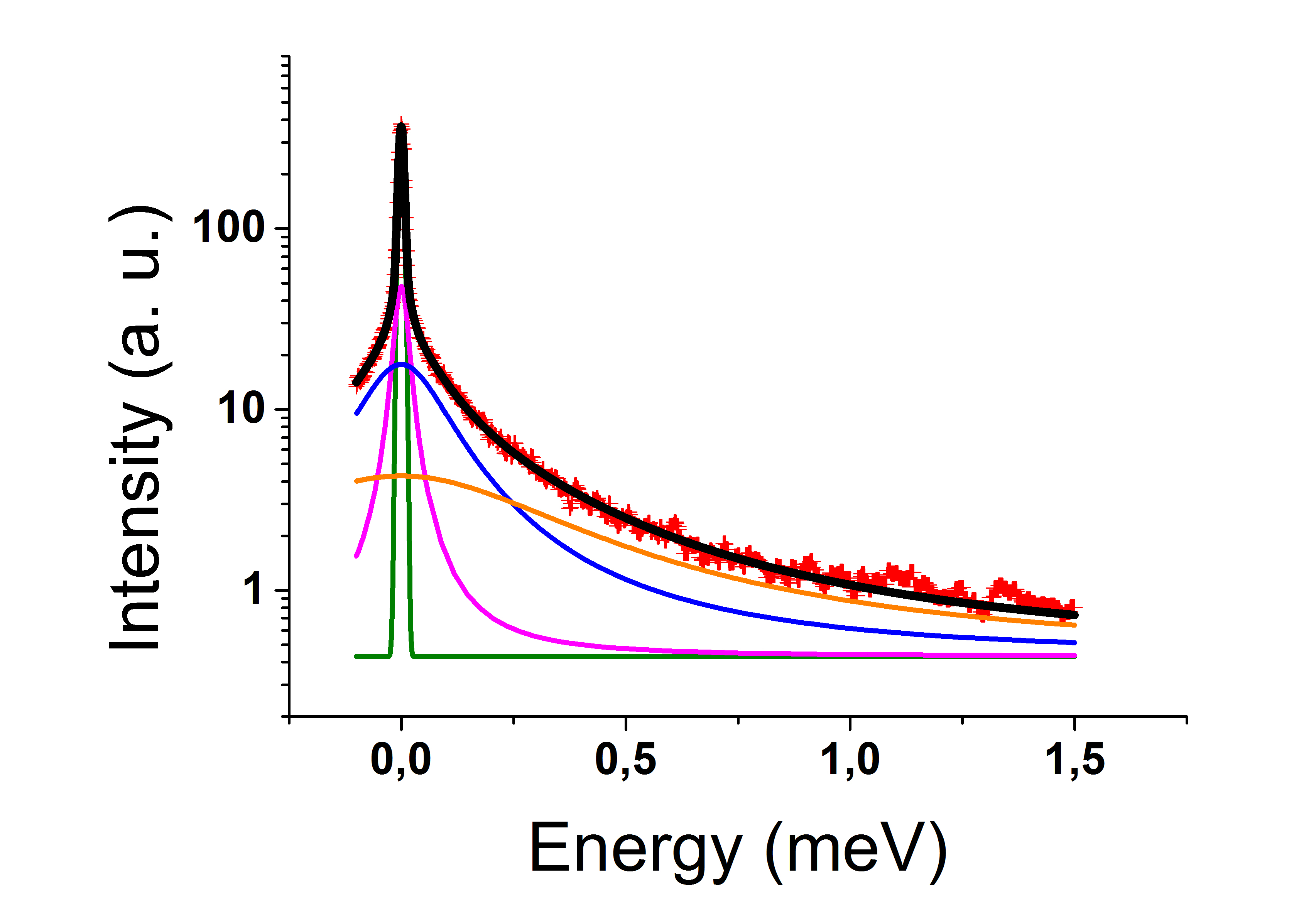 |
|  | 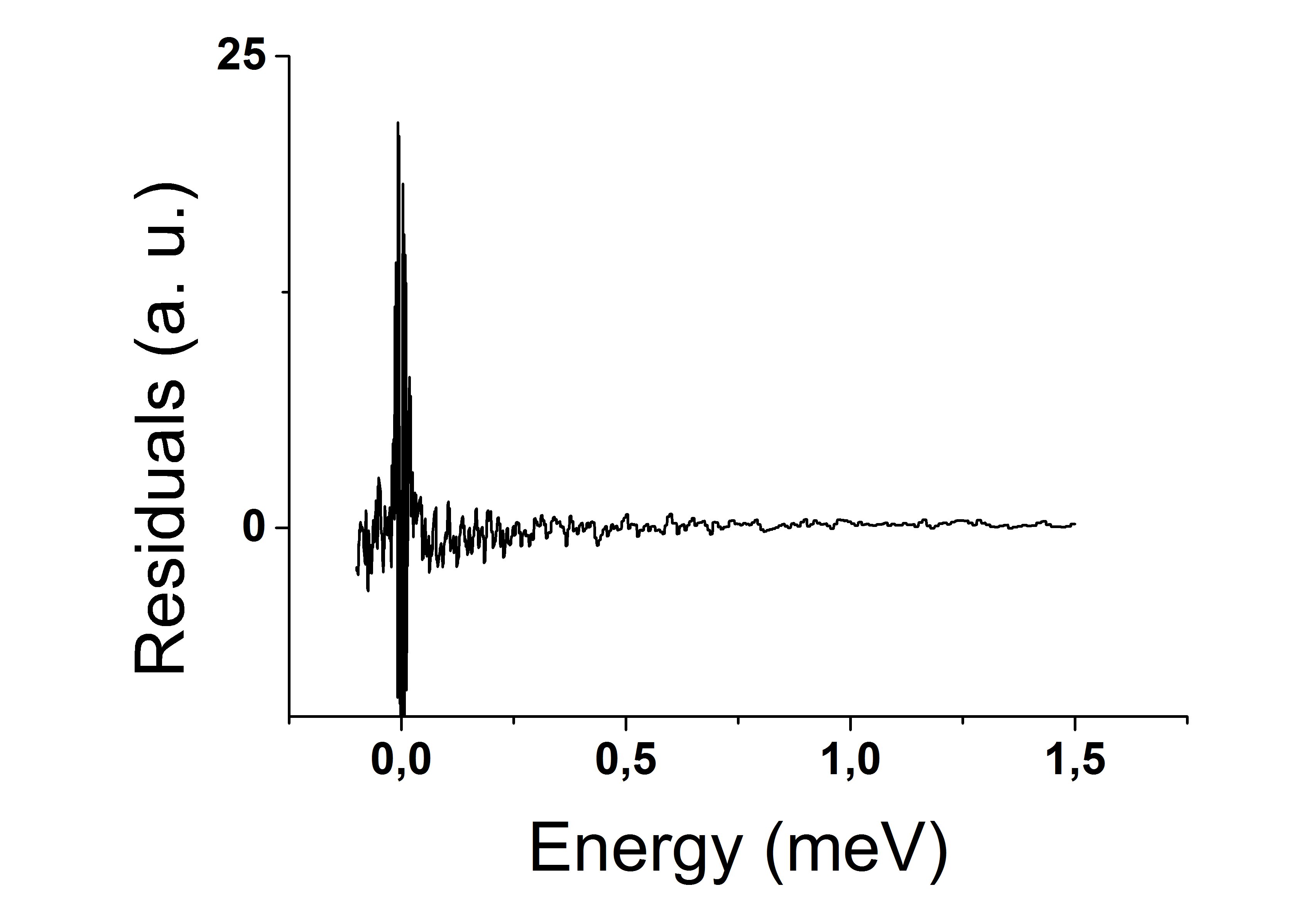 | 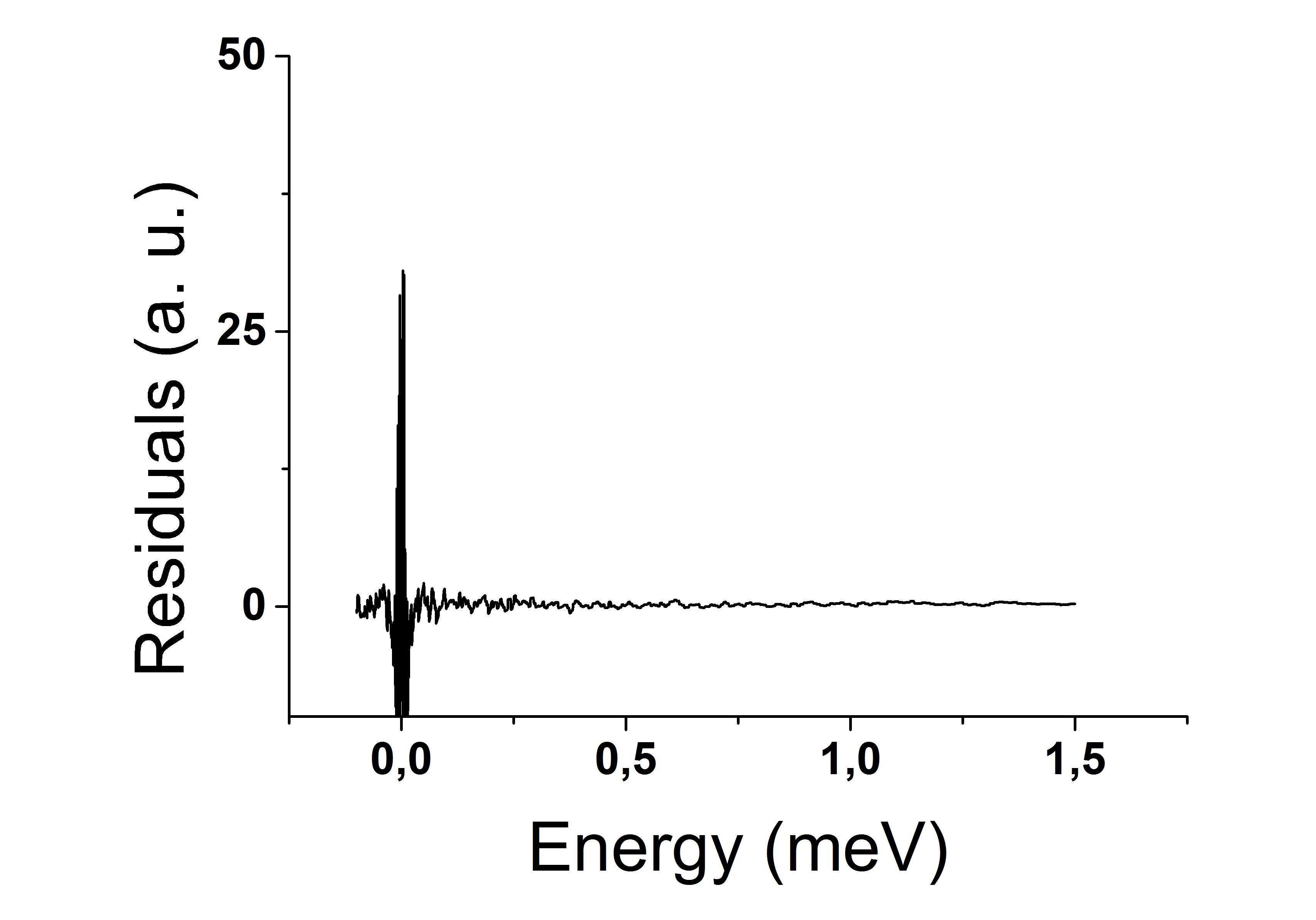 |
| 0.96 | 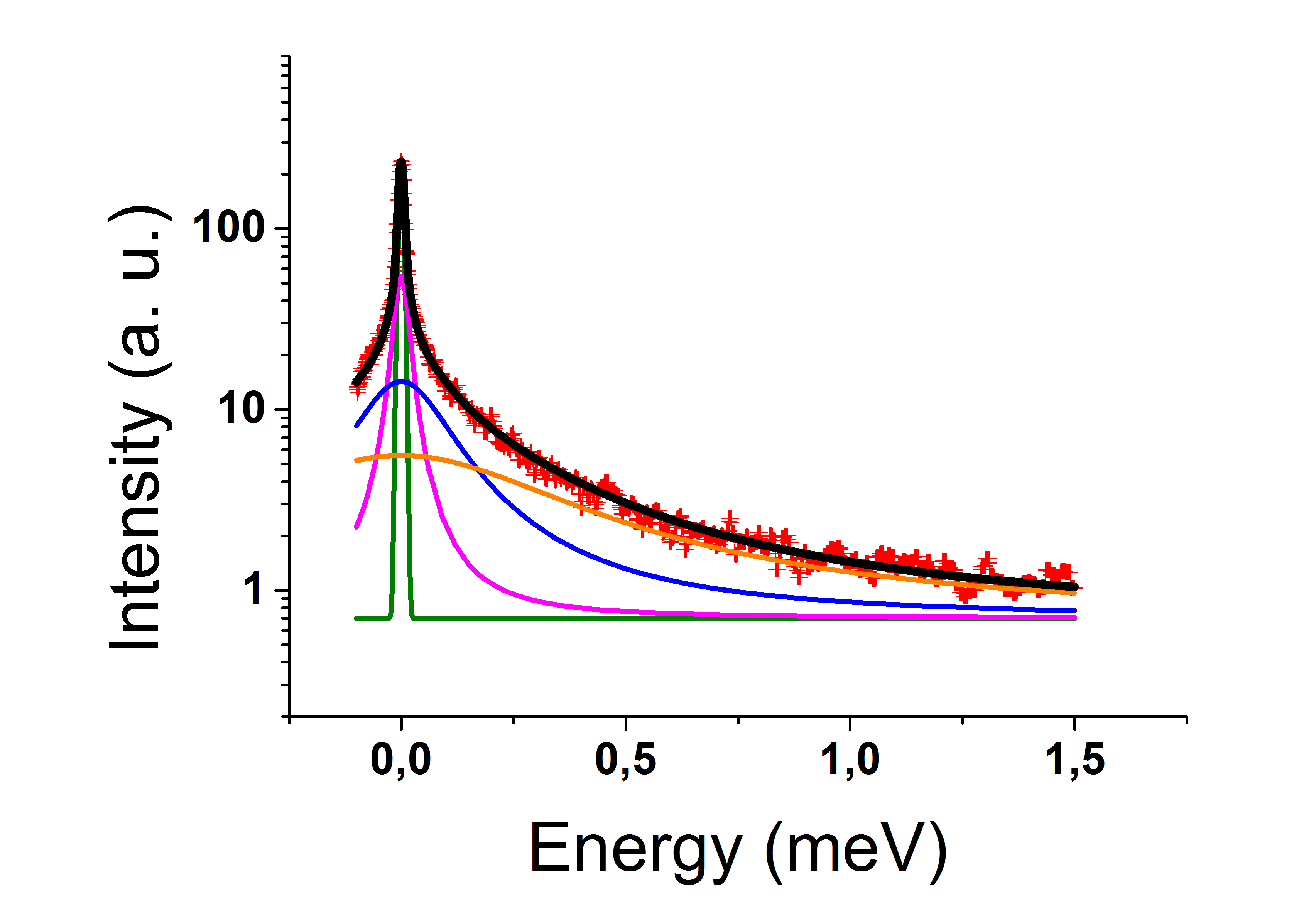 | 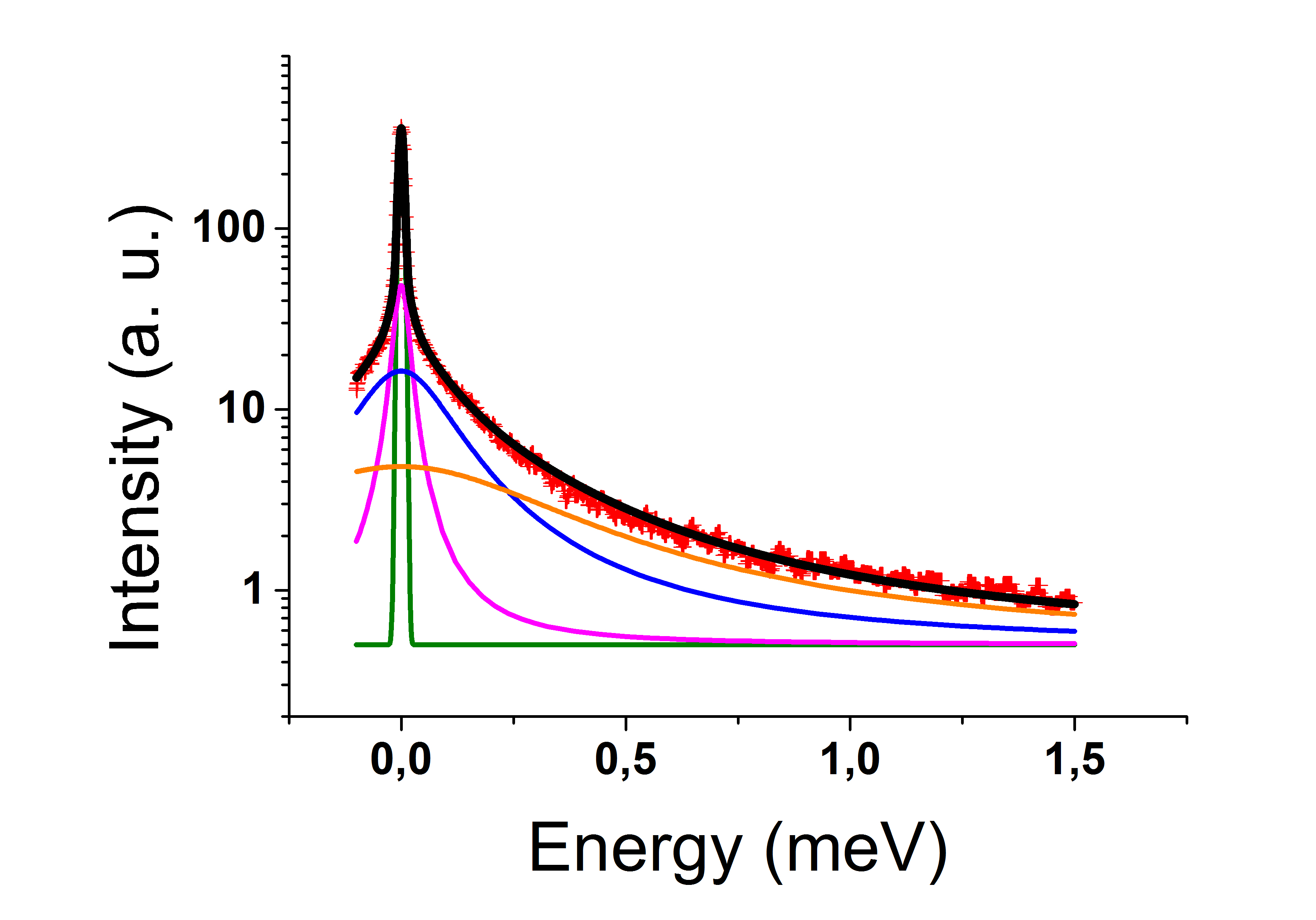 |
|  | 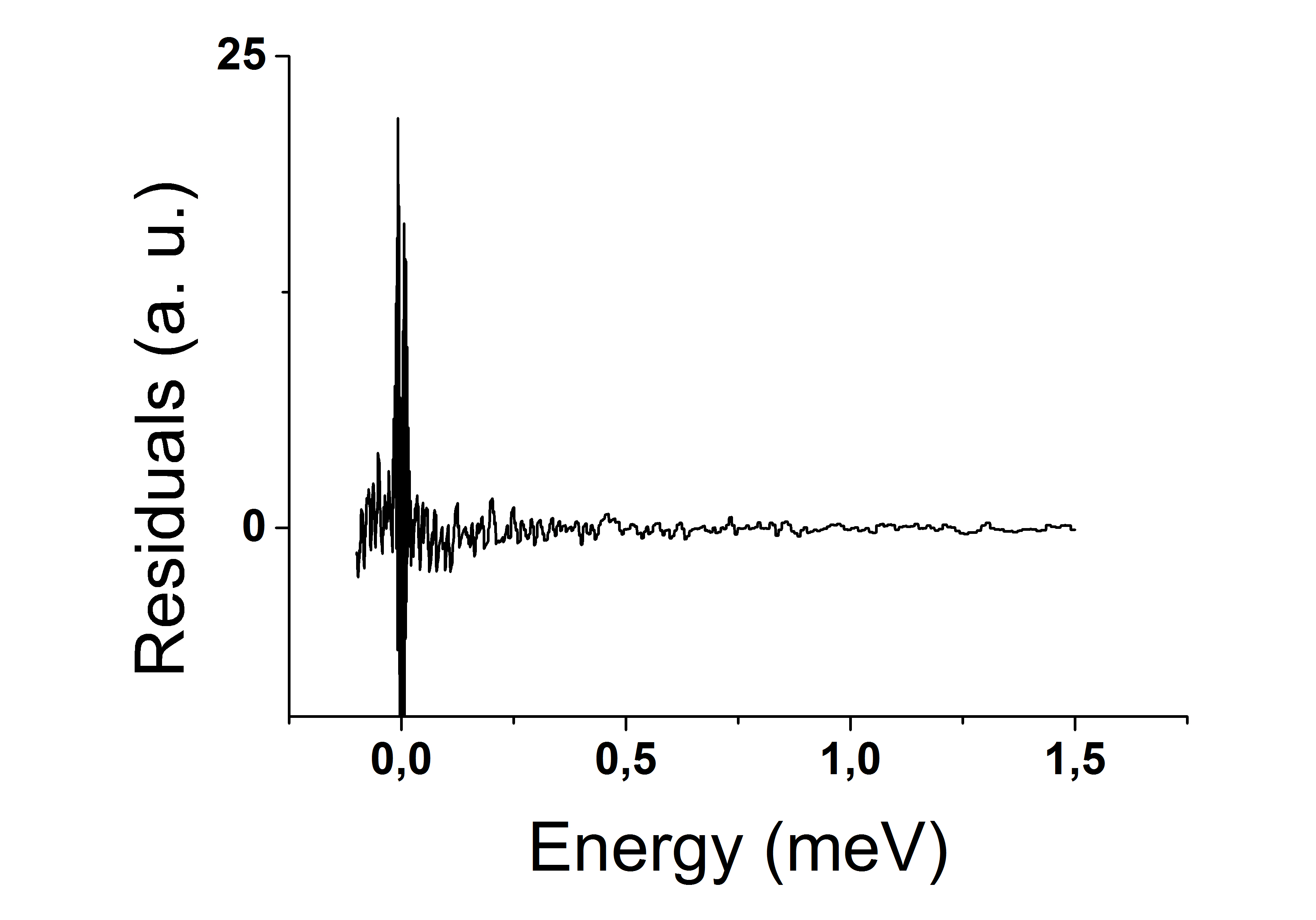 | 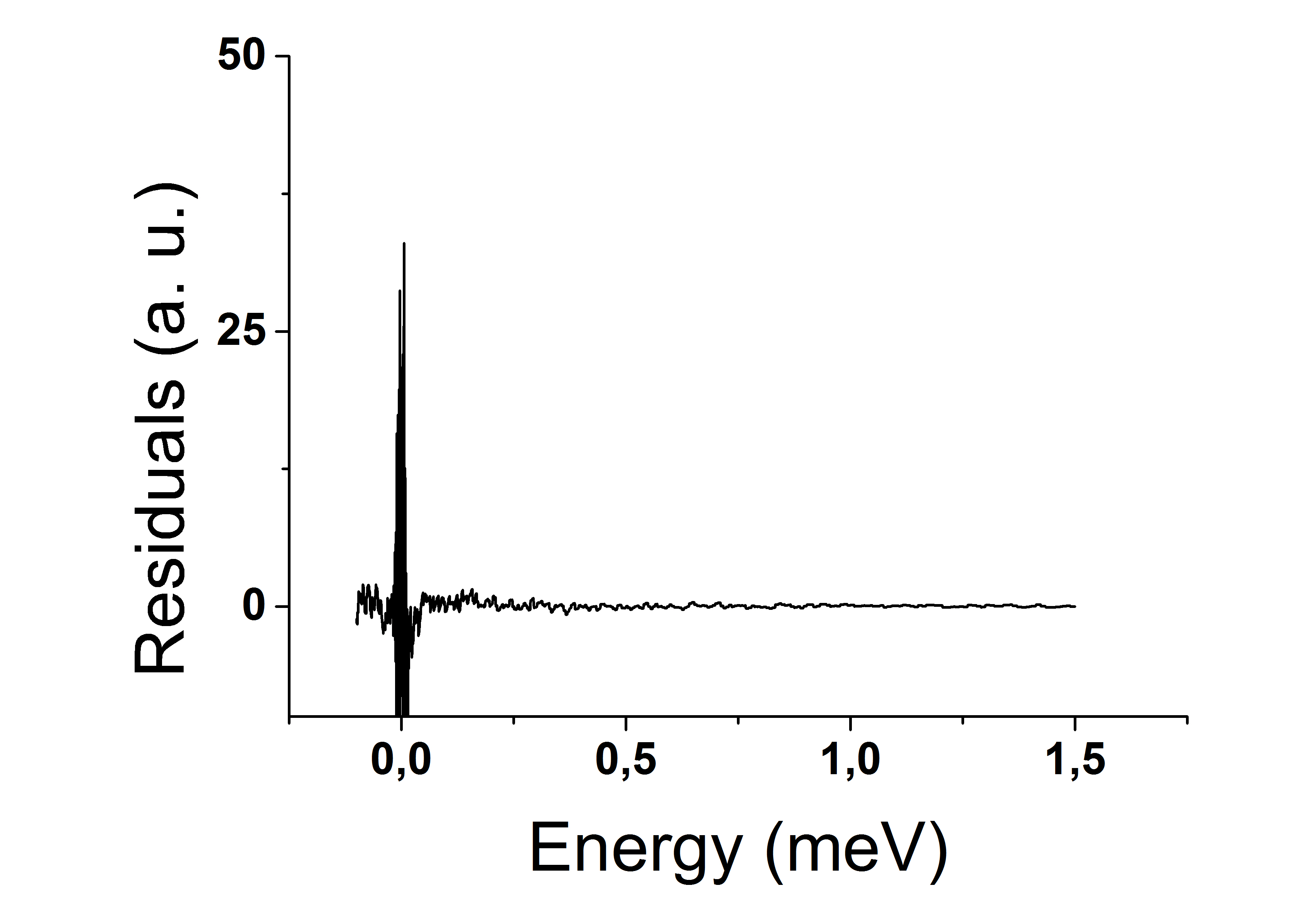 |
| 1.00 | 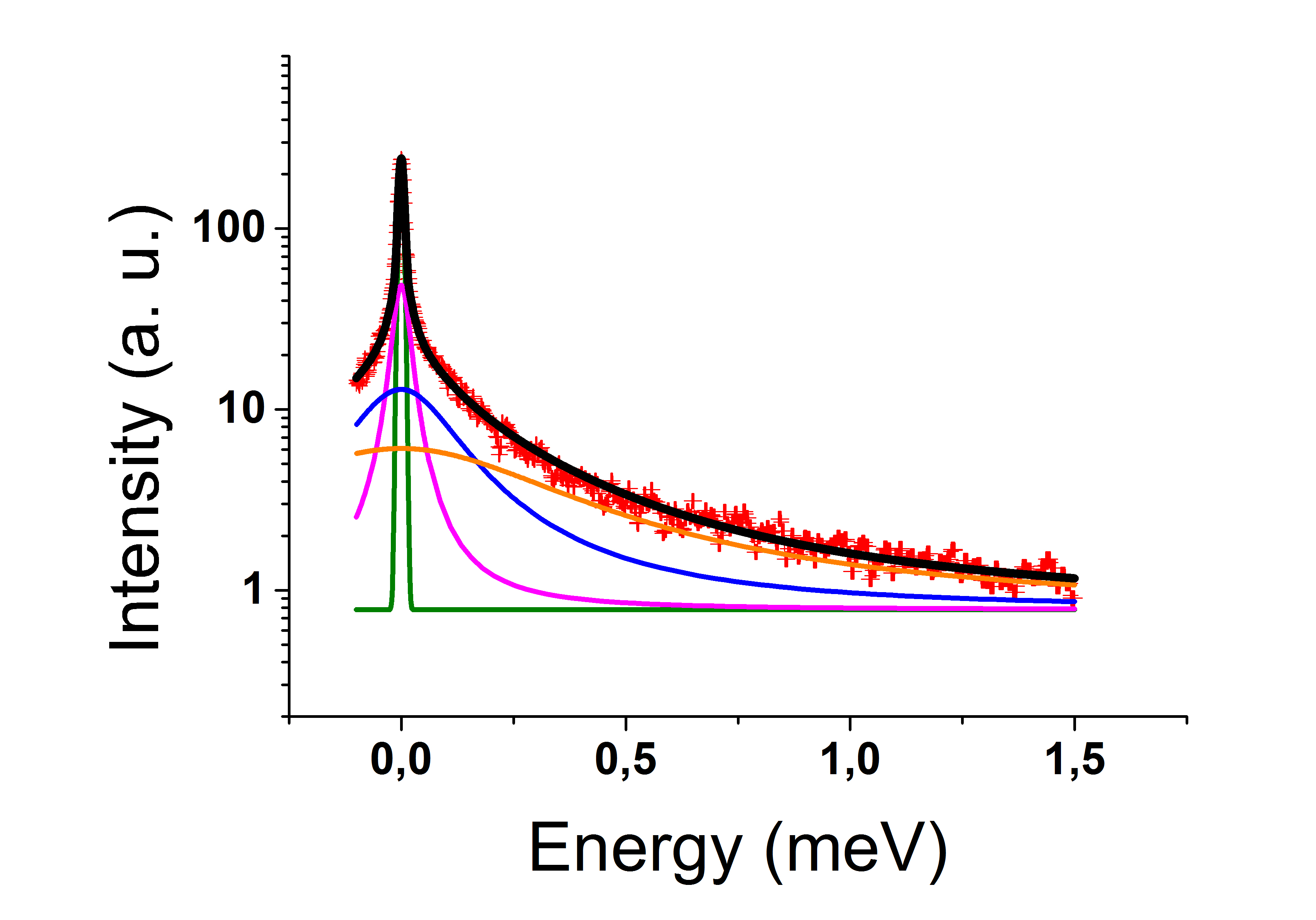 | 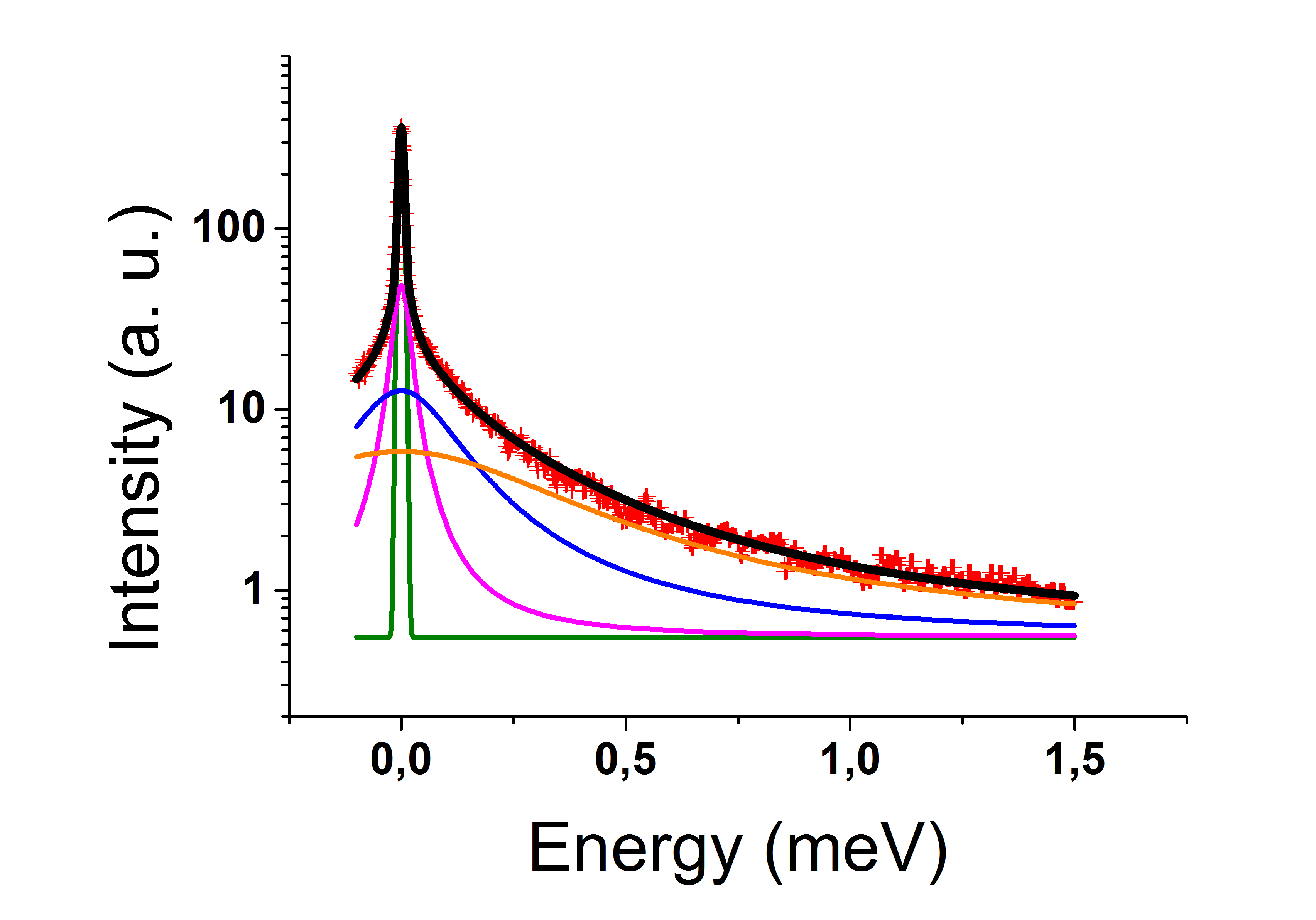 |
|  | 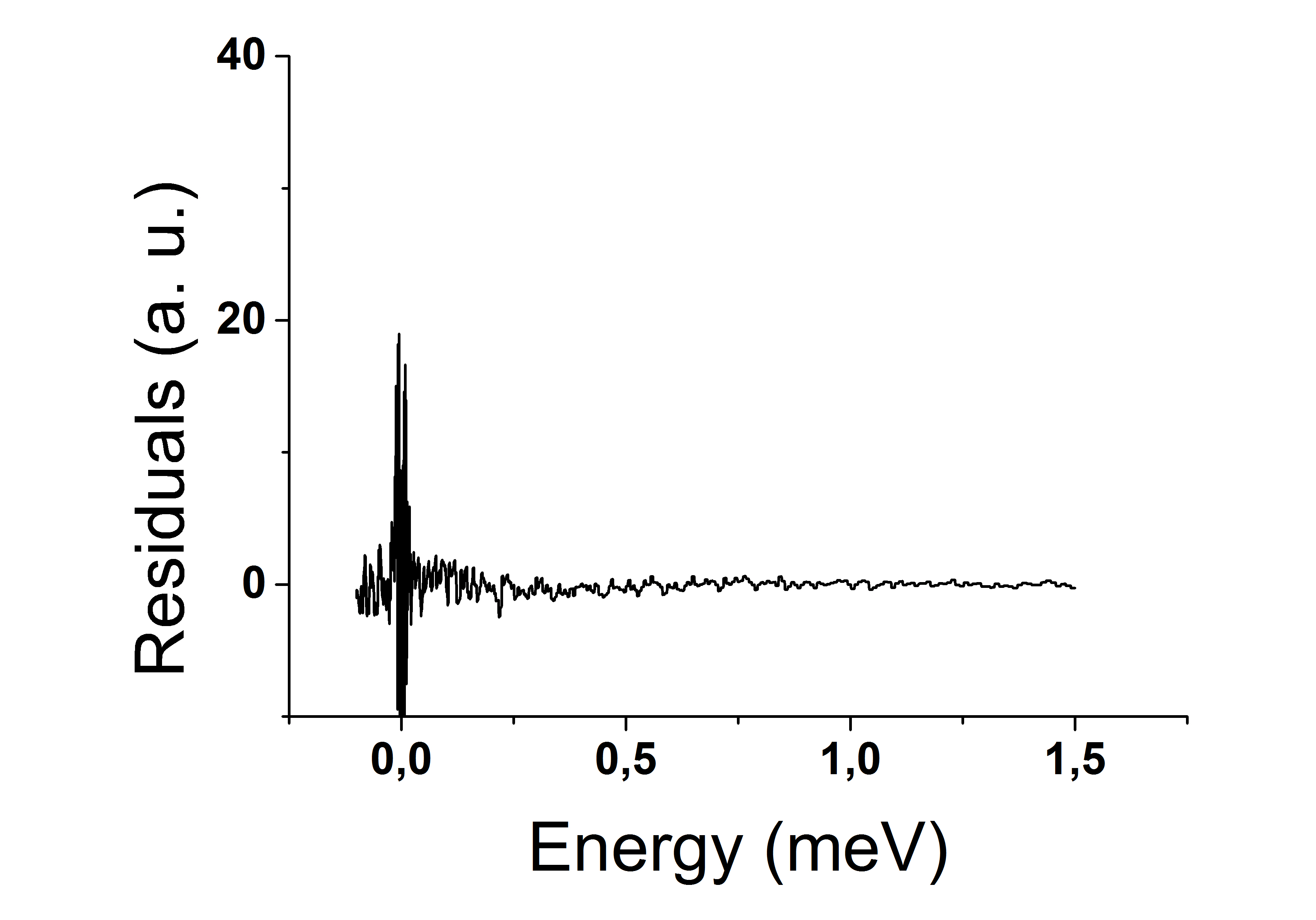 | 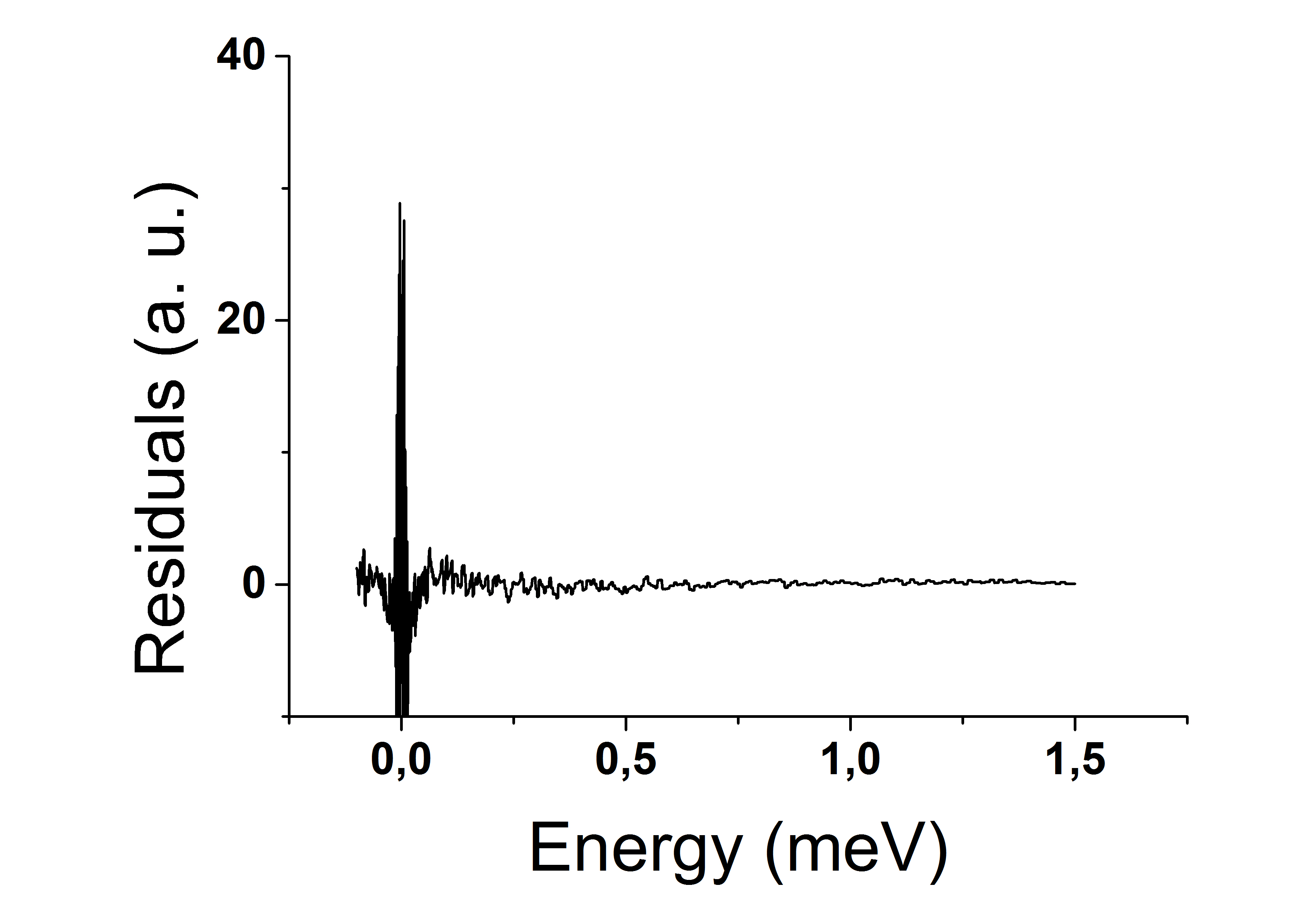 |

IN6

| TG-LDL sample | | |
| --- | --- | --- |
| Q (Å^-1^) | 20 bar | 3000 bar |
| 0.37 | 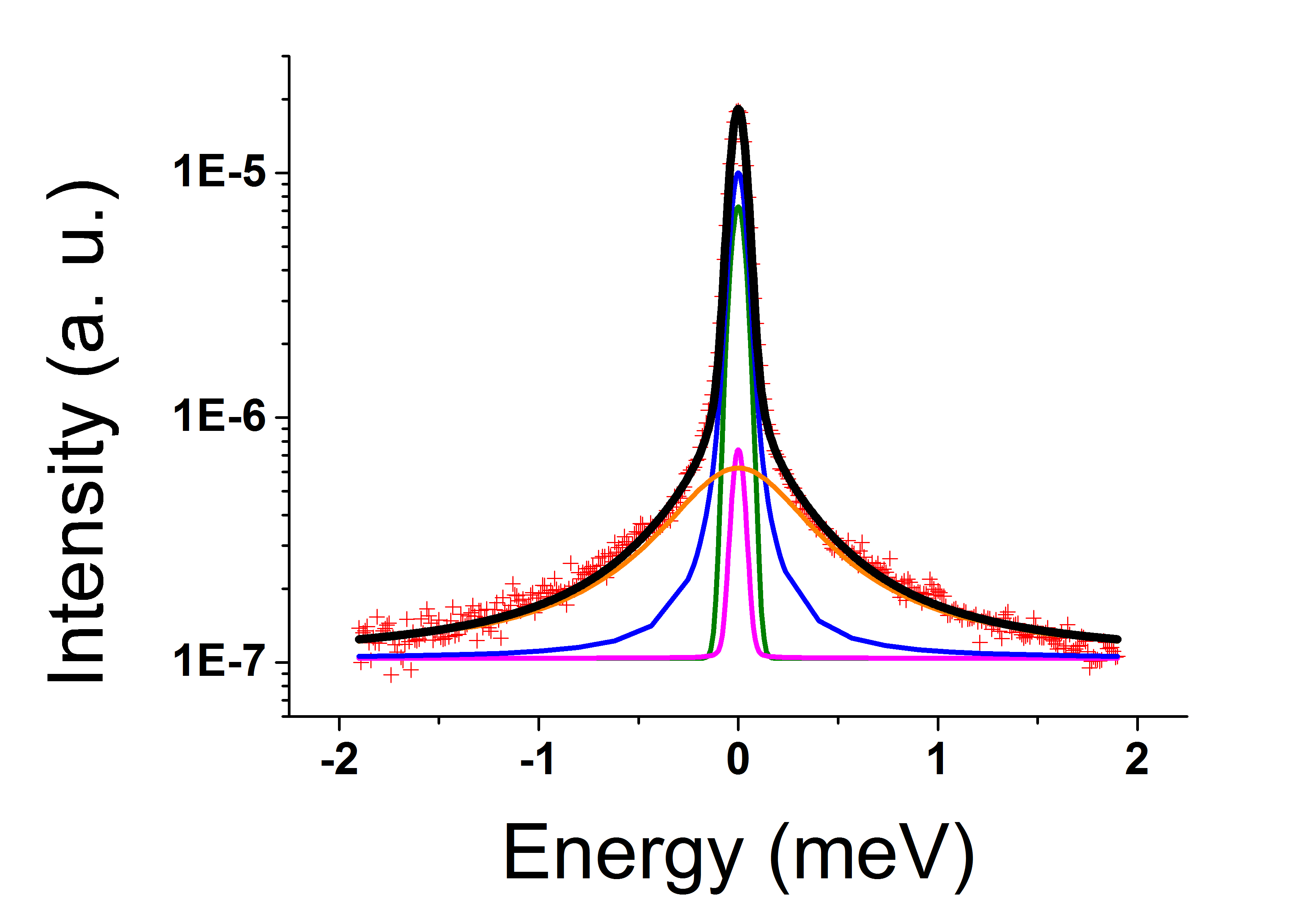 | 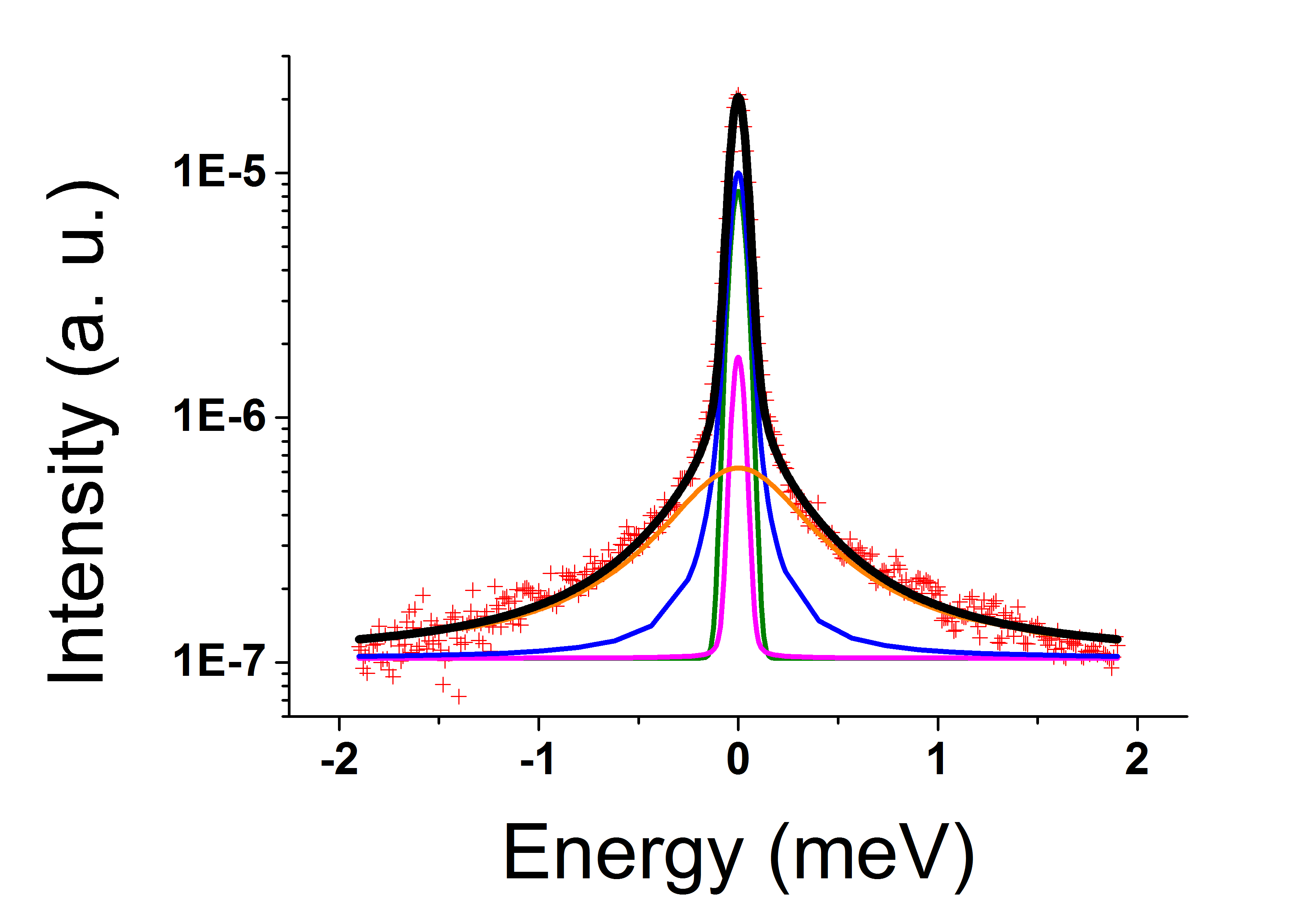 |
|  | 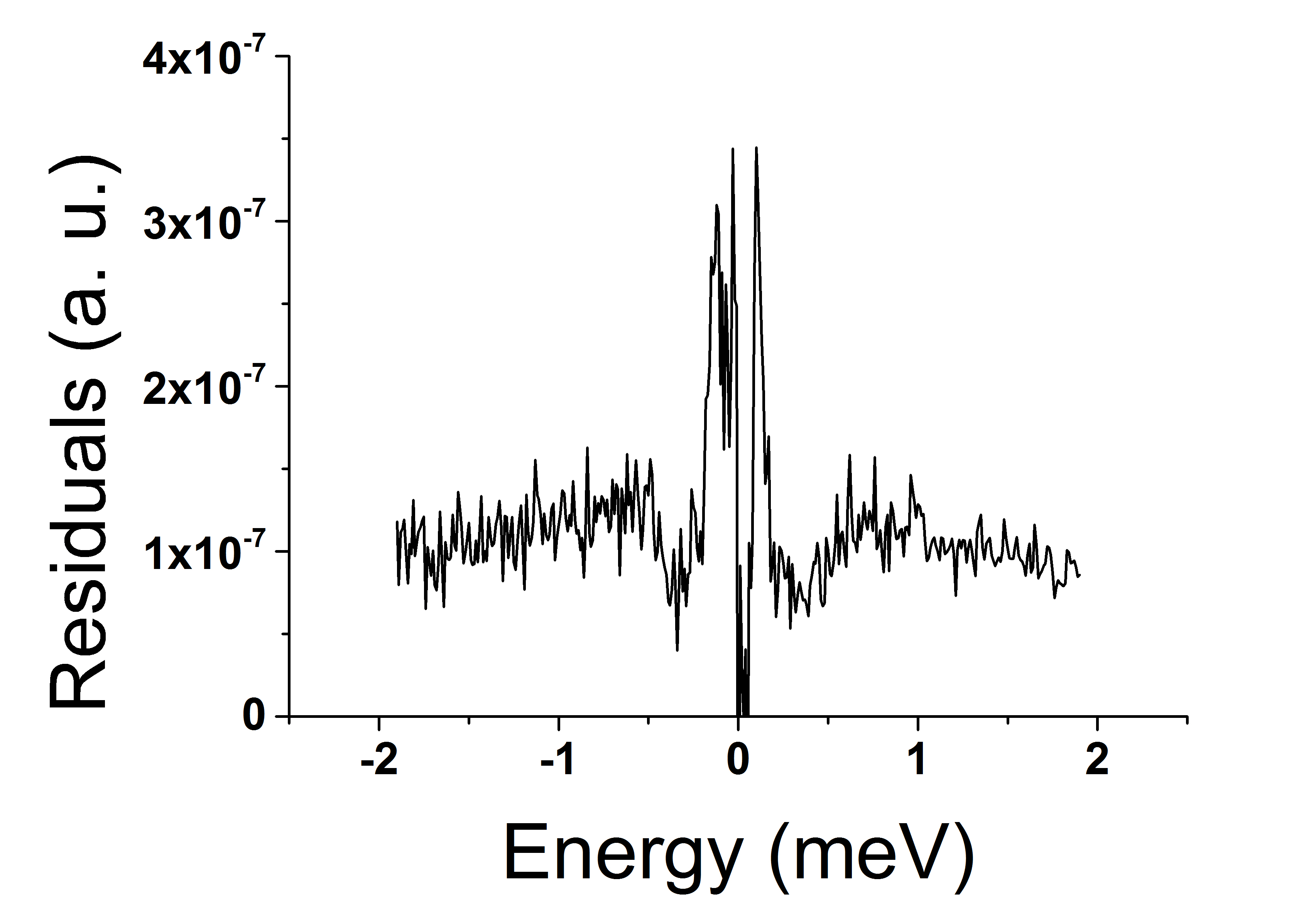 | 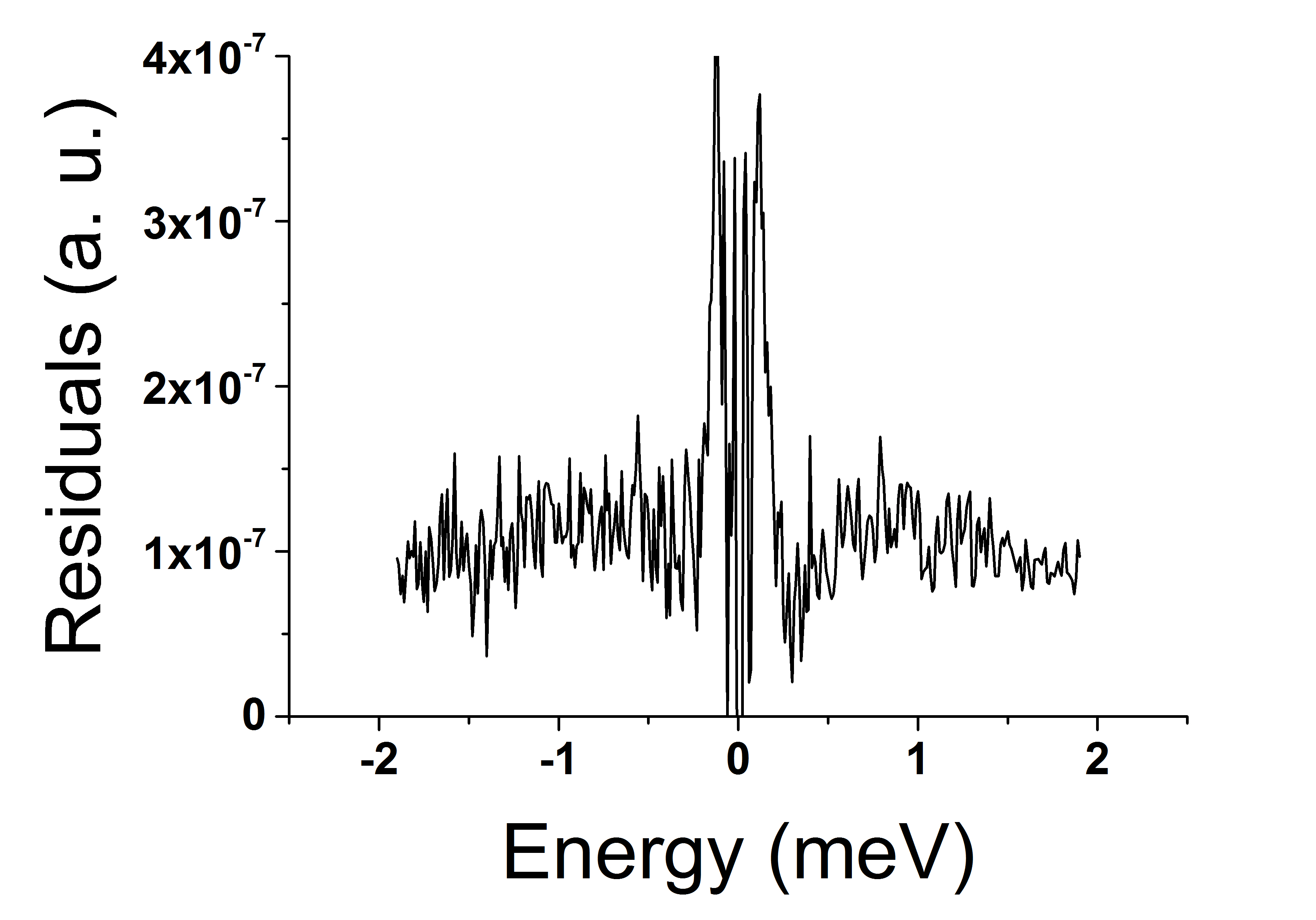 |
| 0.55 | 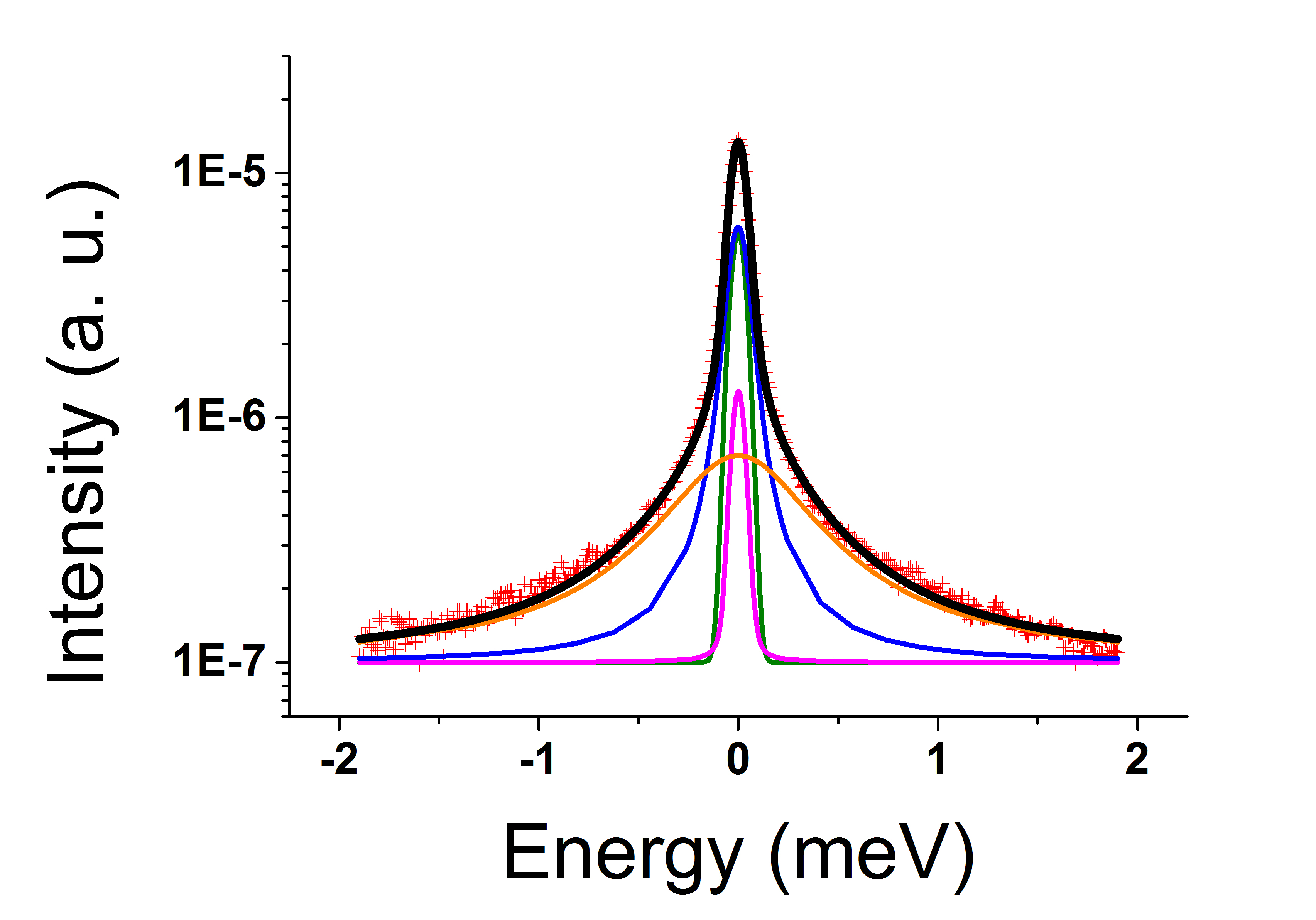 | 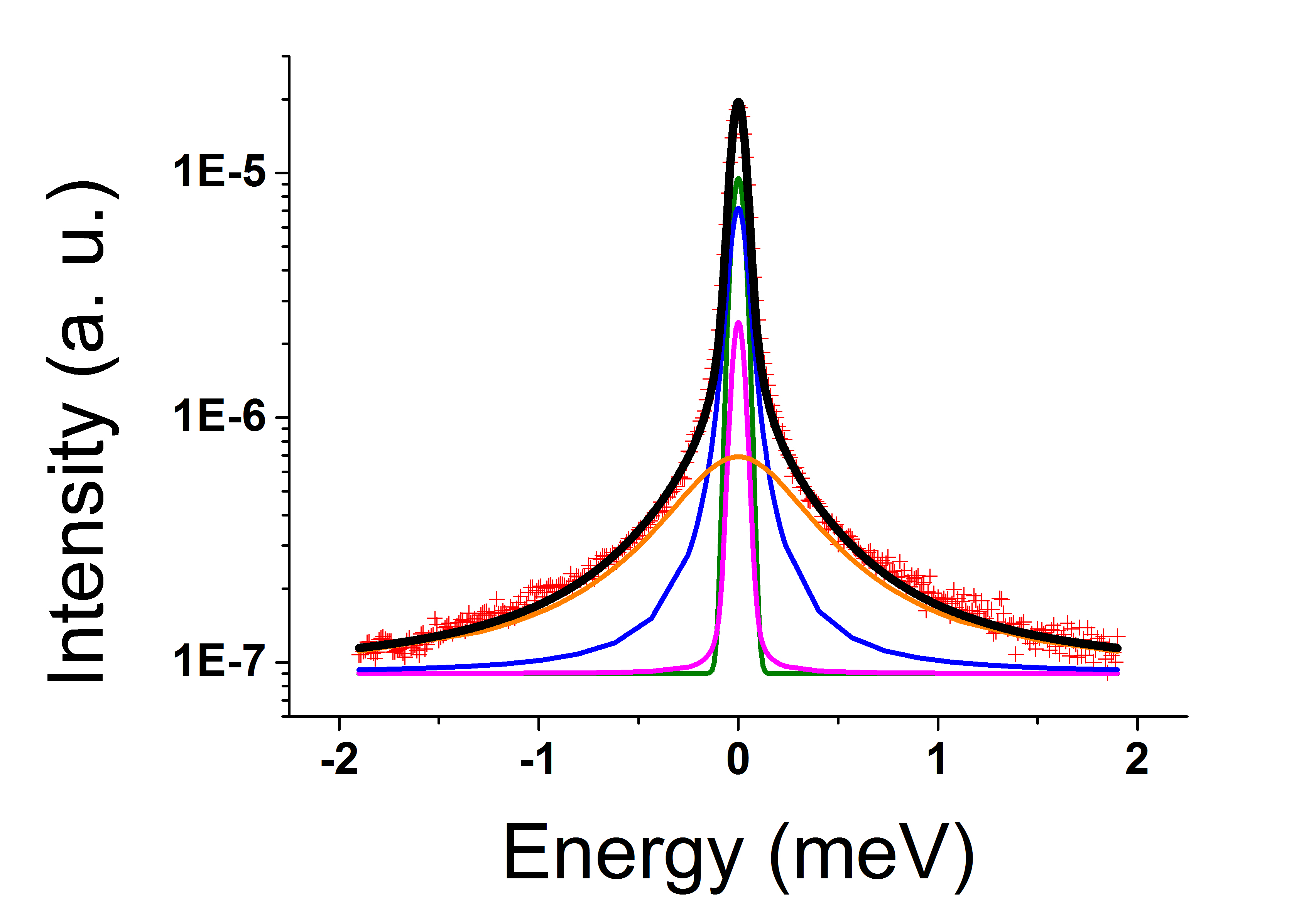 |
|  | 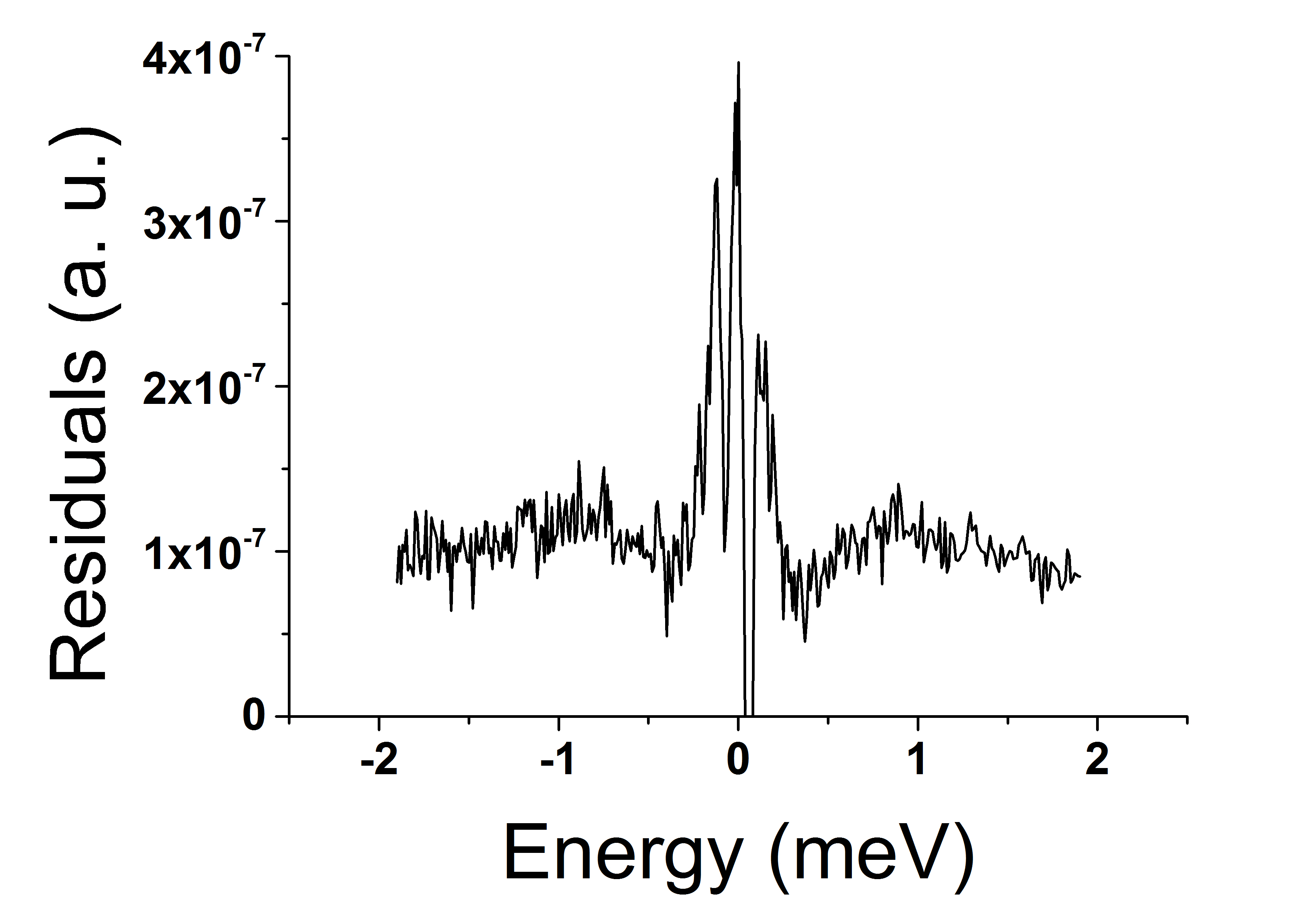 | 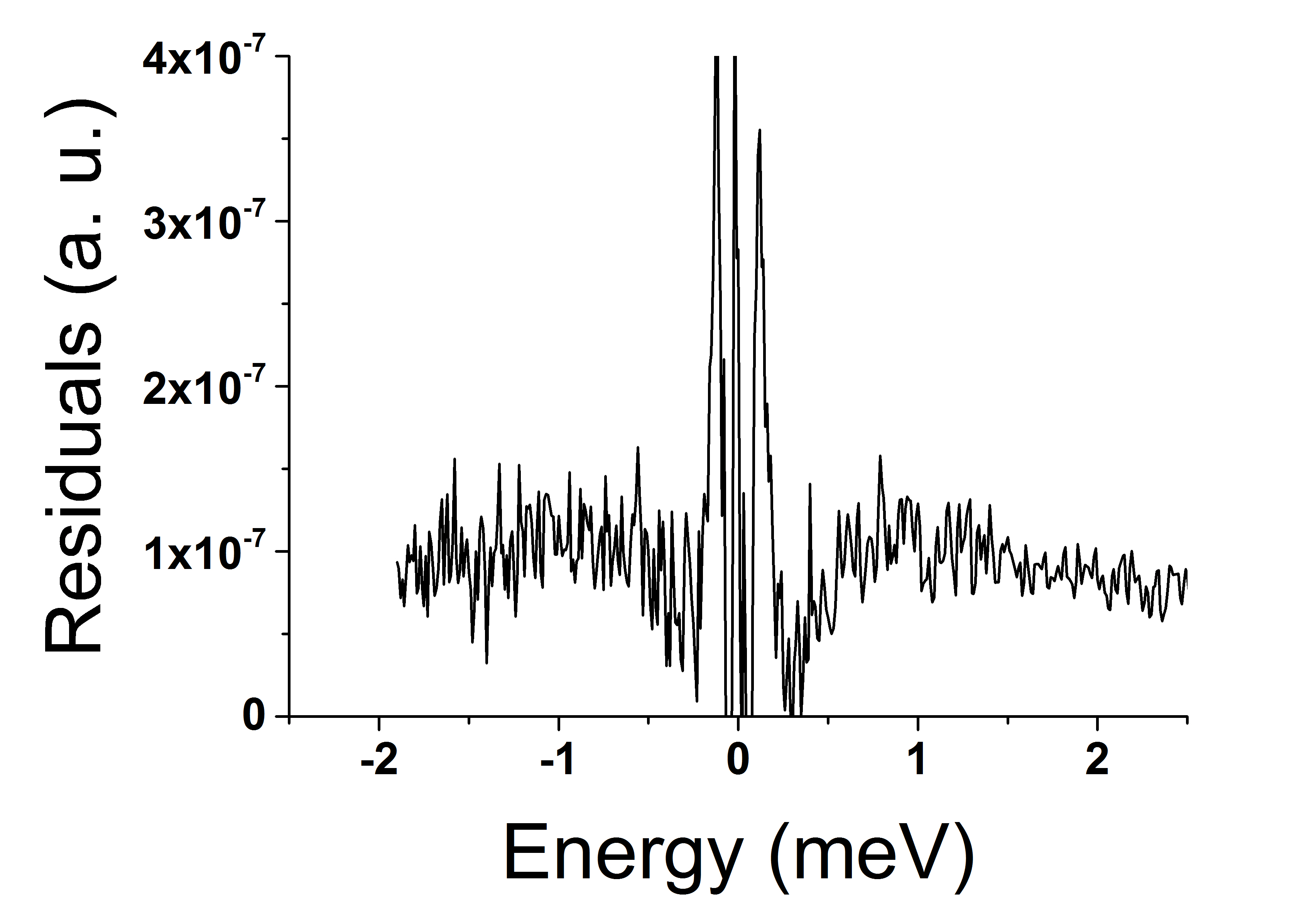 |
| 0.73 | 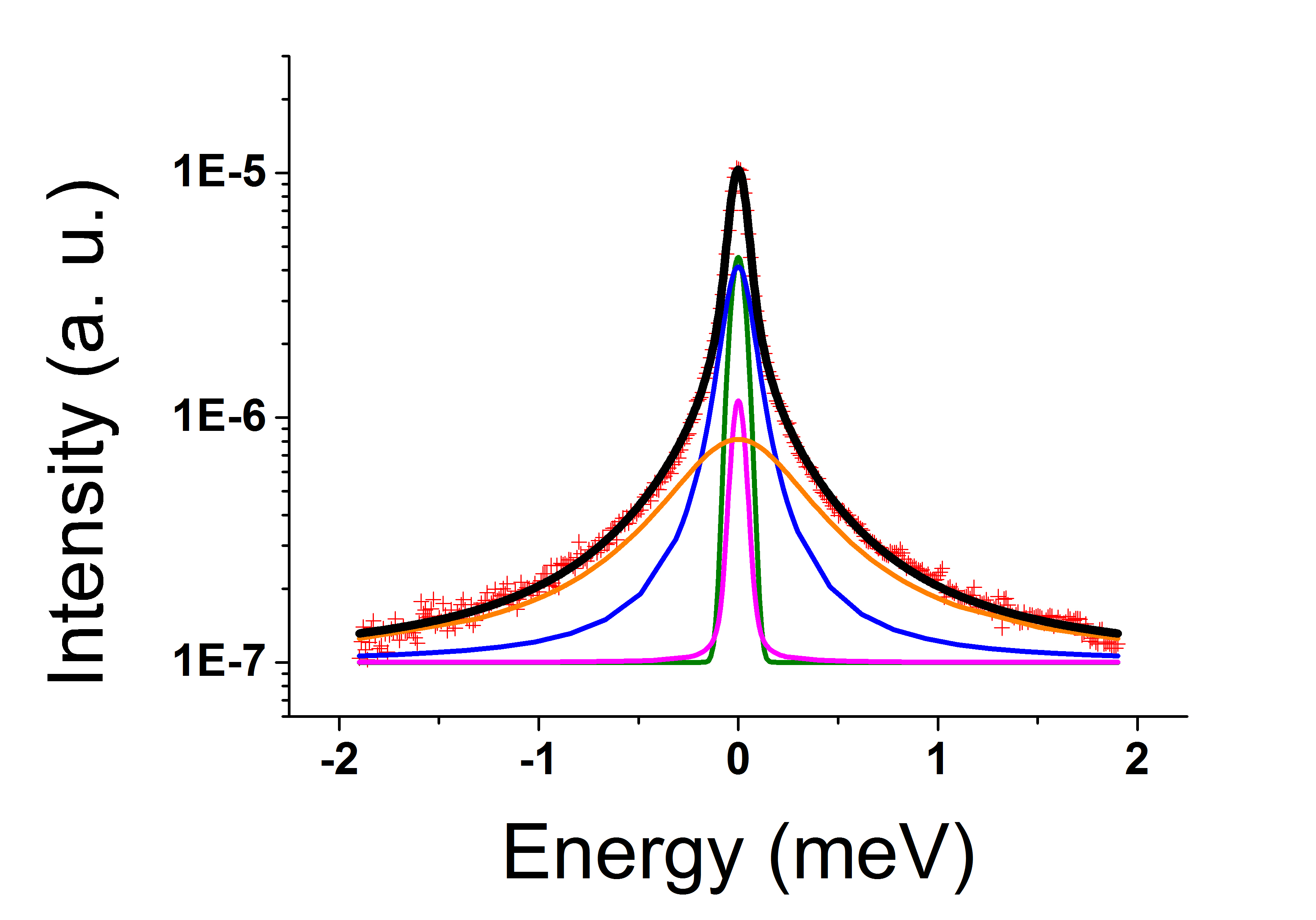 | 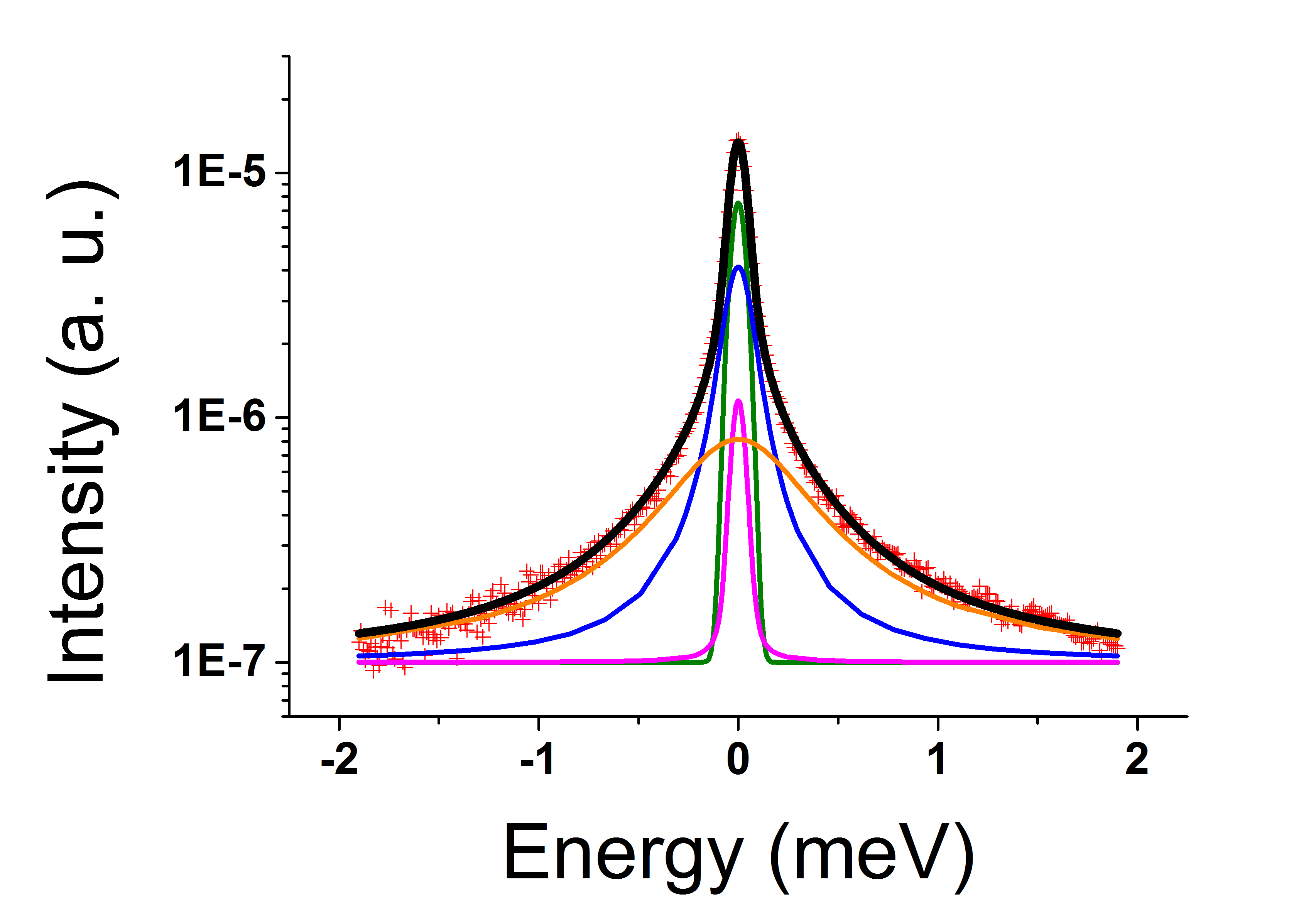 |
|  | 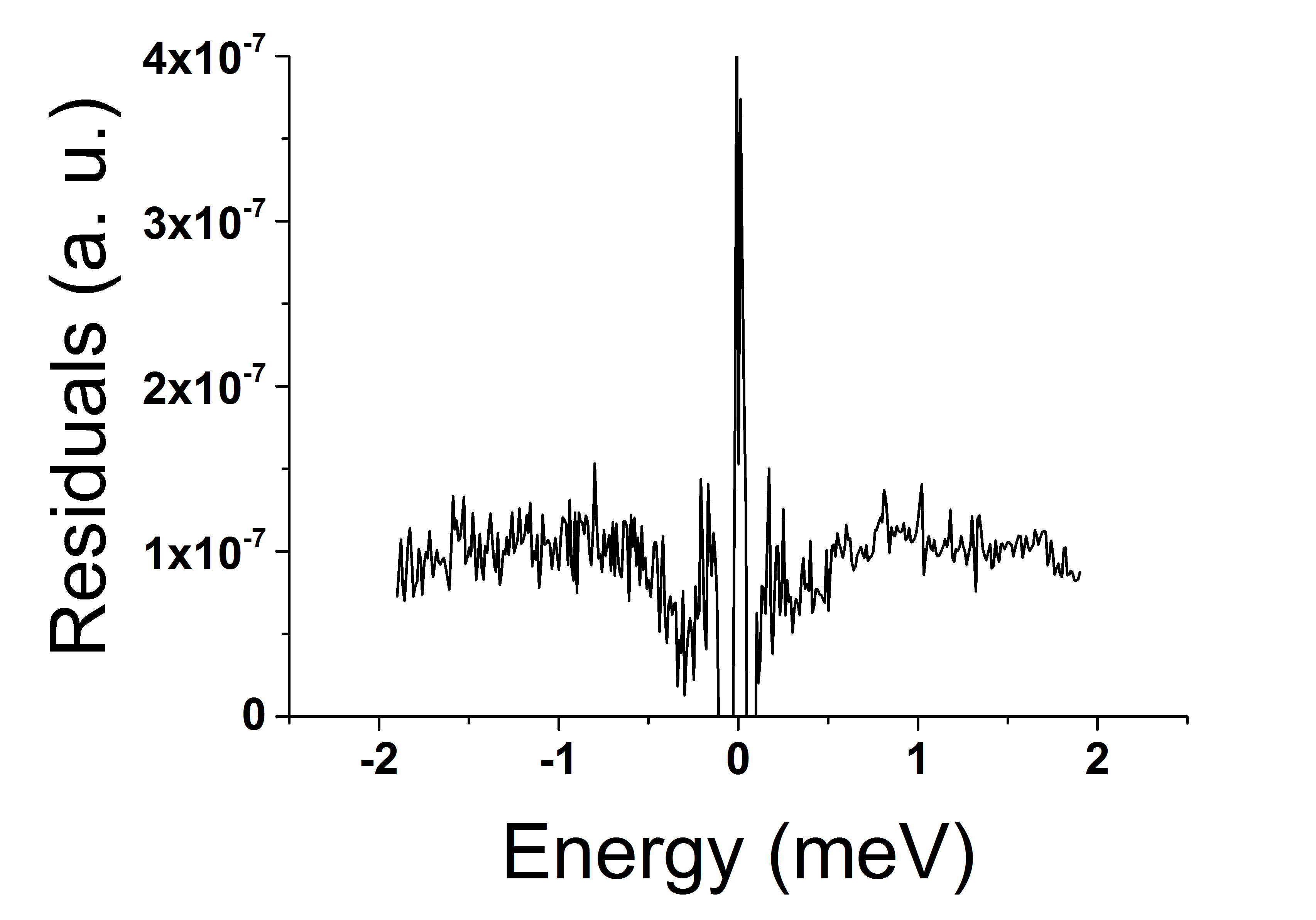 | 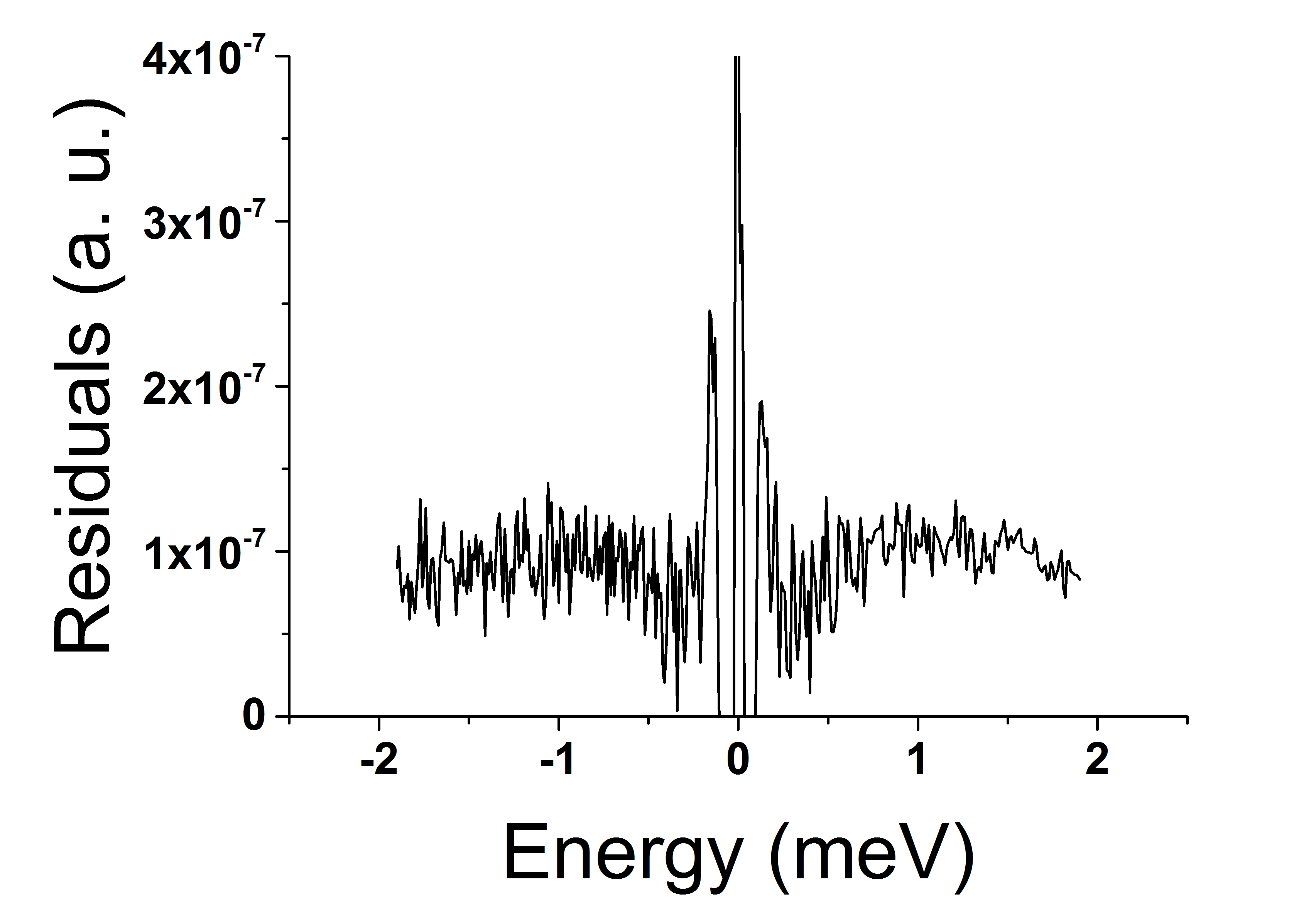 |
| 0.9 | 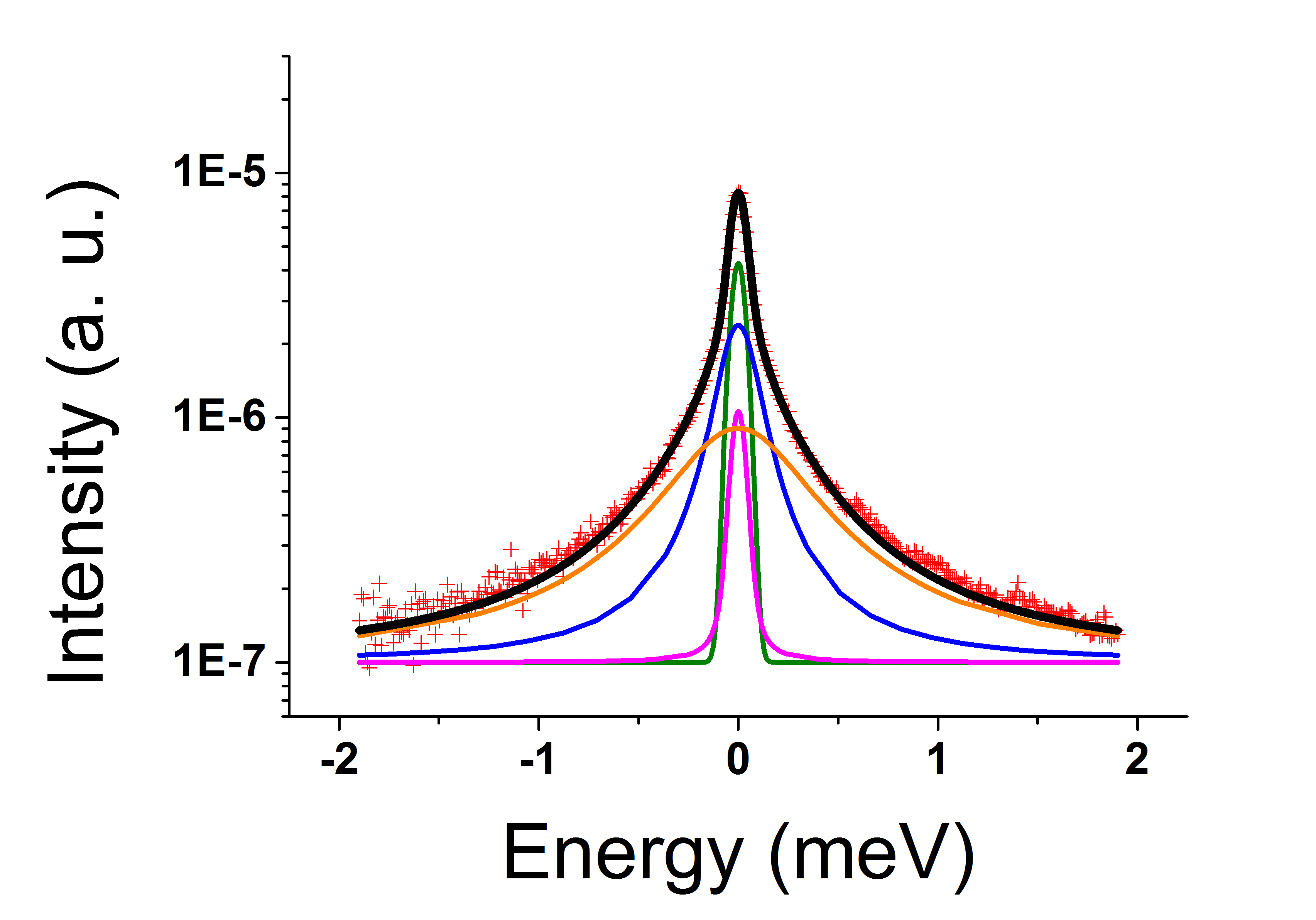 | 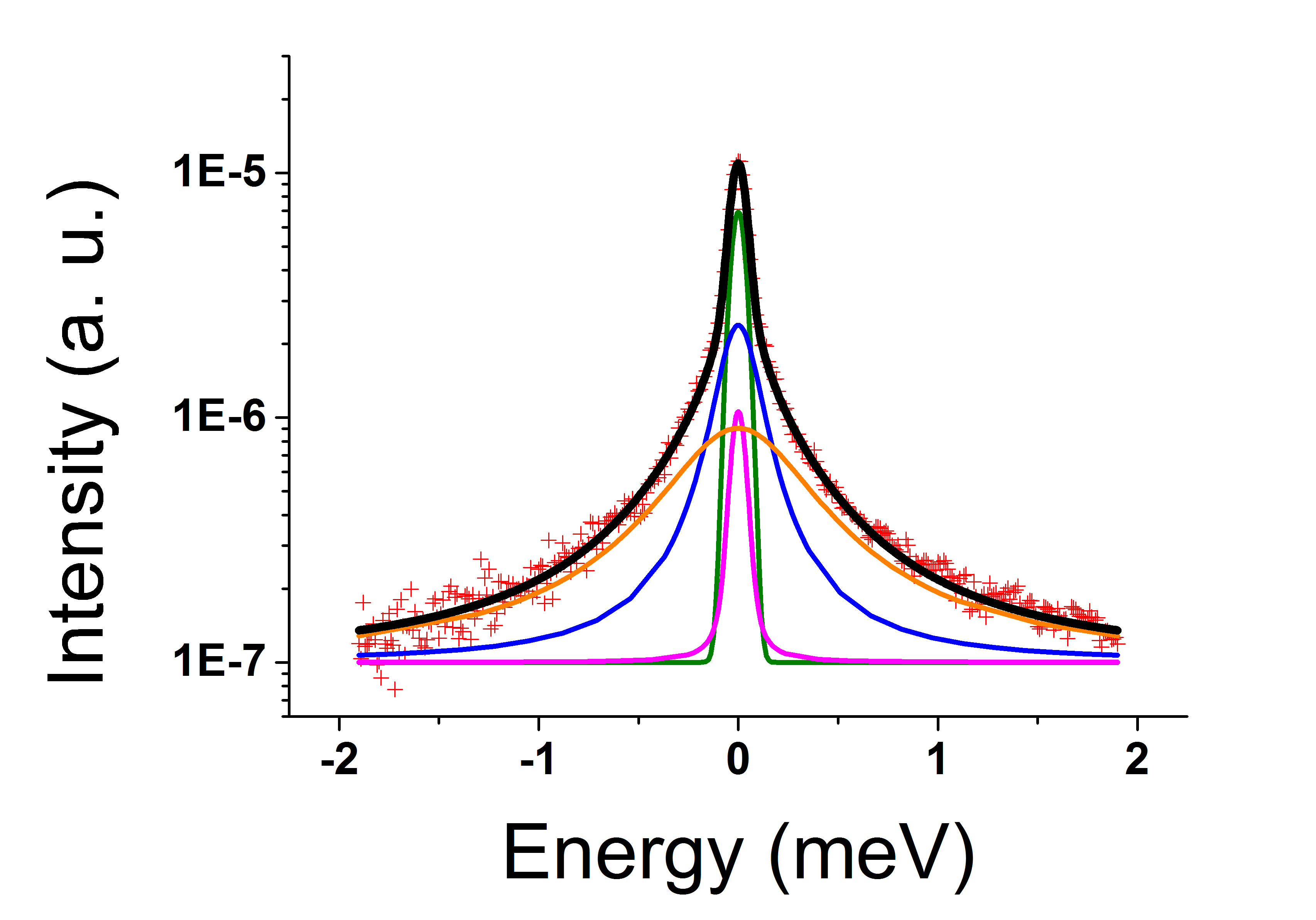 |
|  | 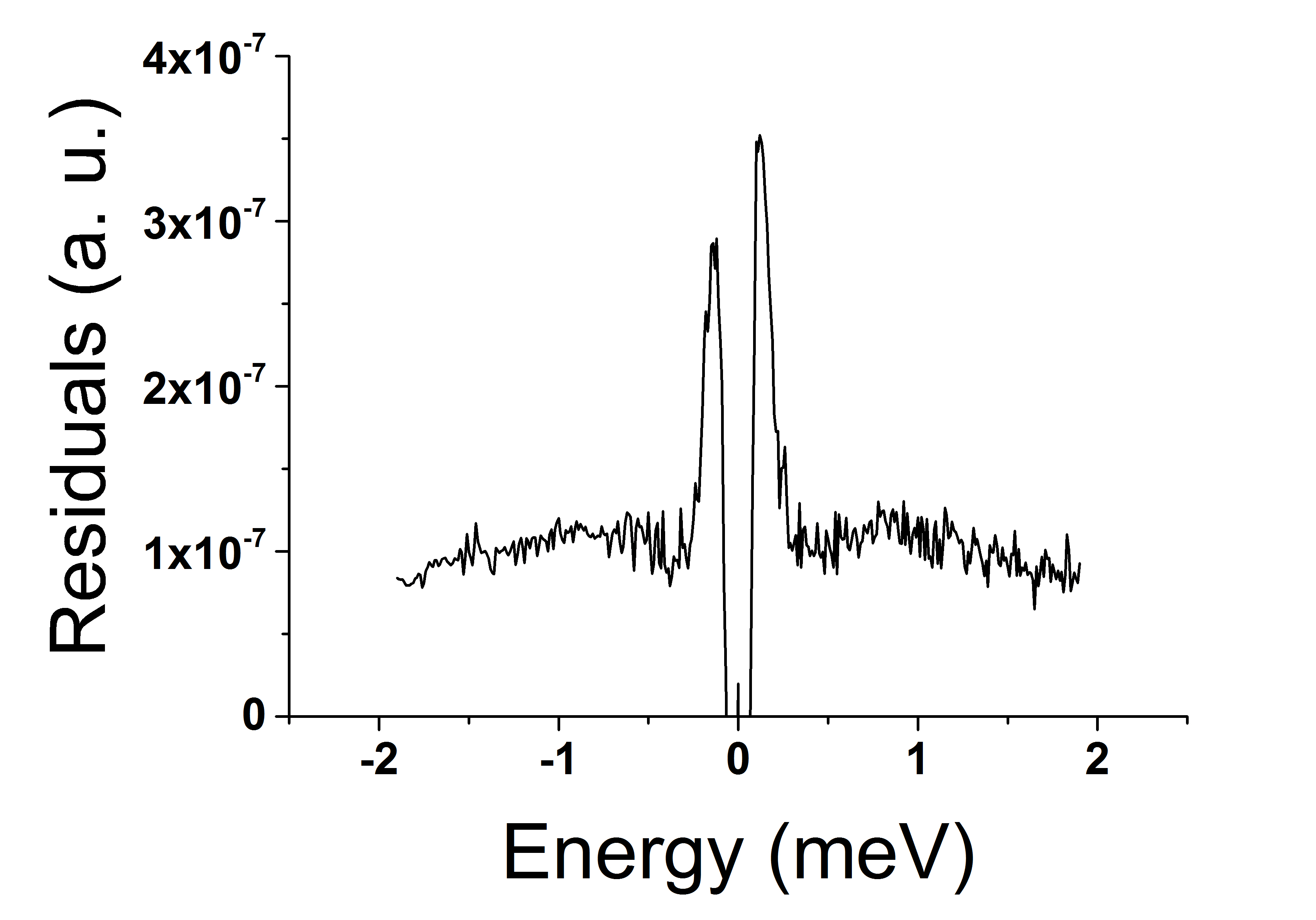 | 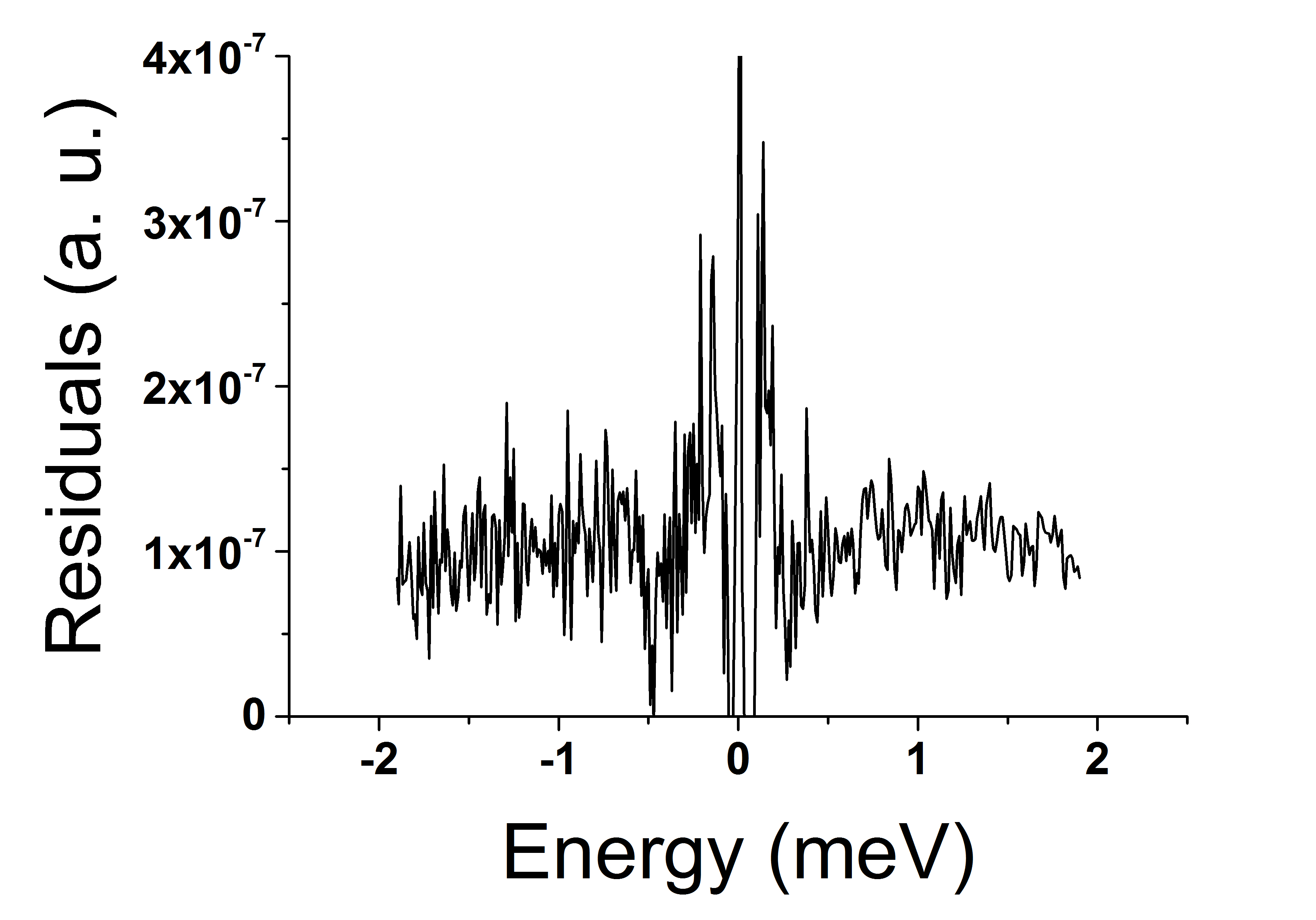 |
| 1.07 | 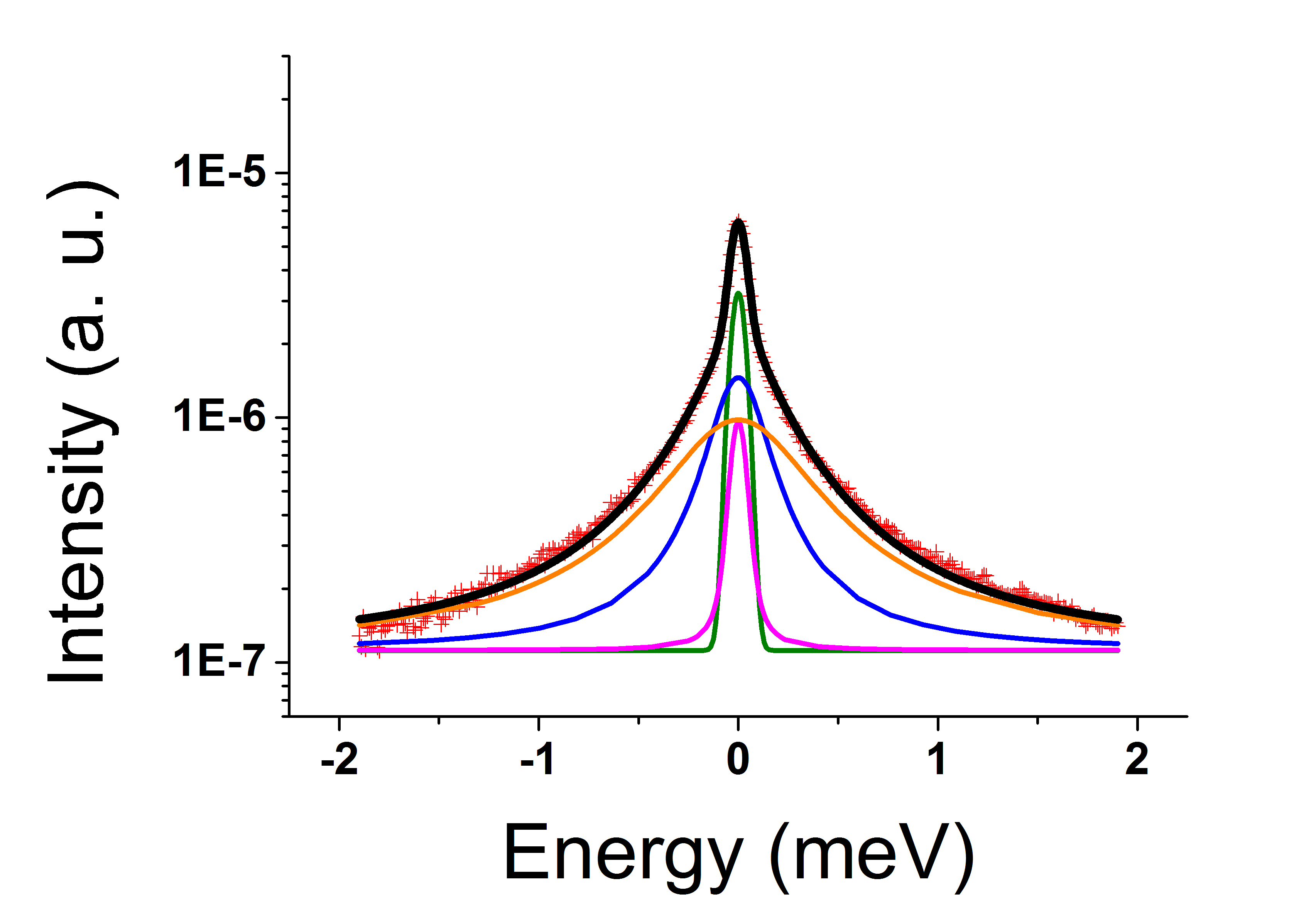 | 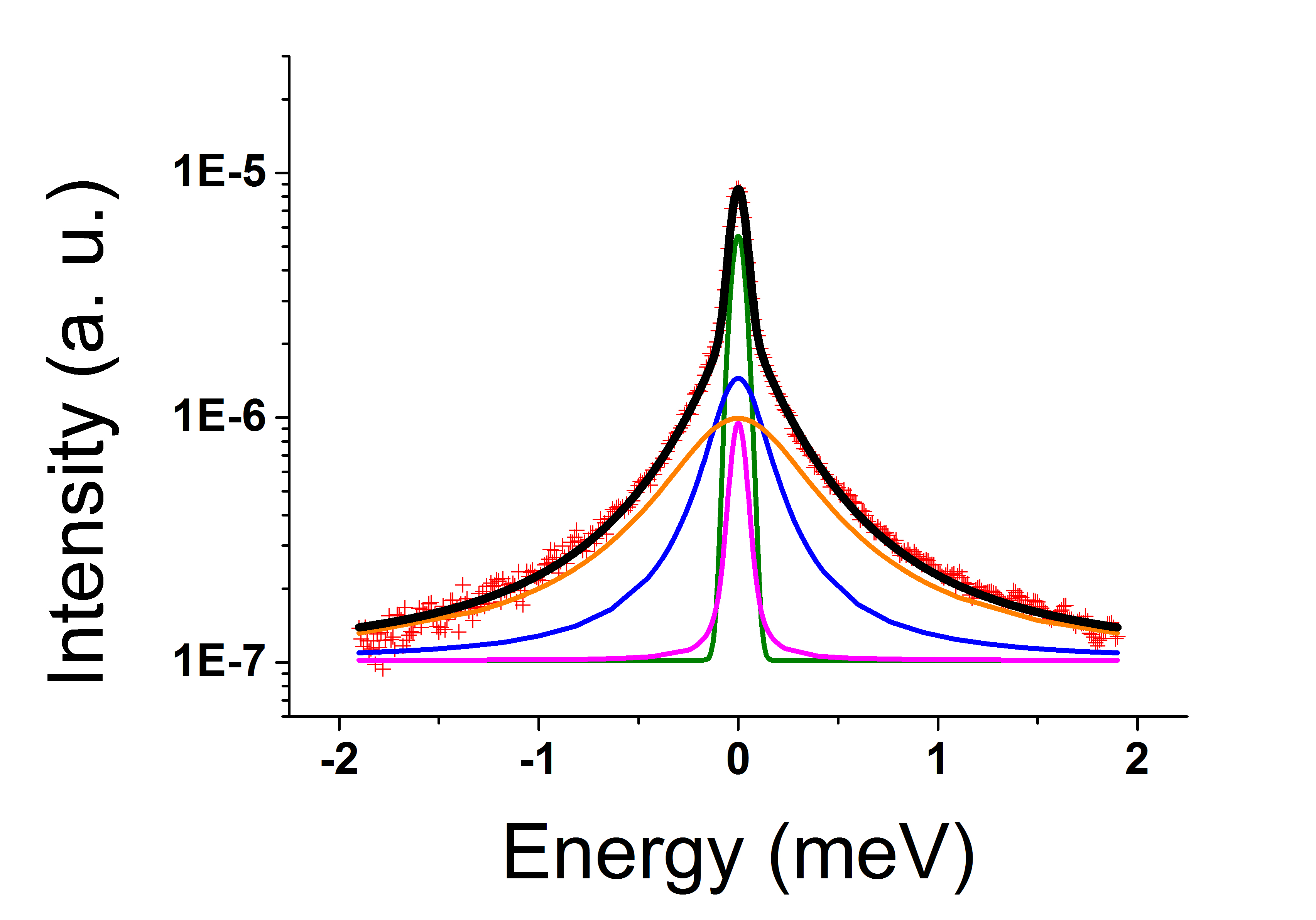 |
|  | 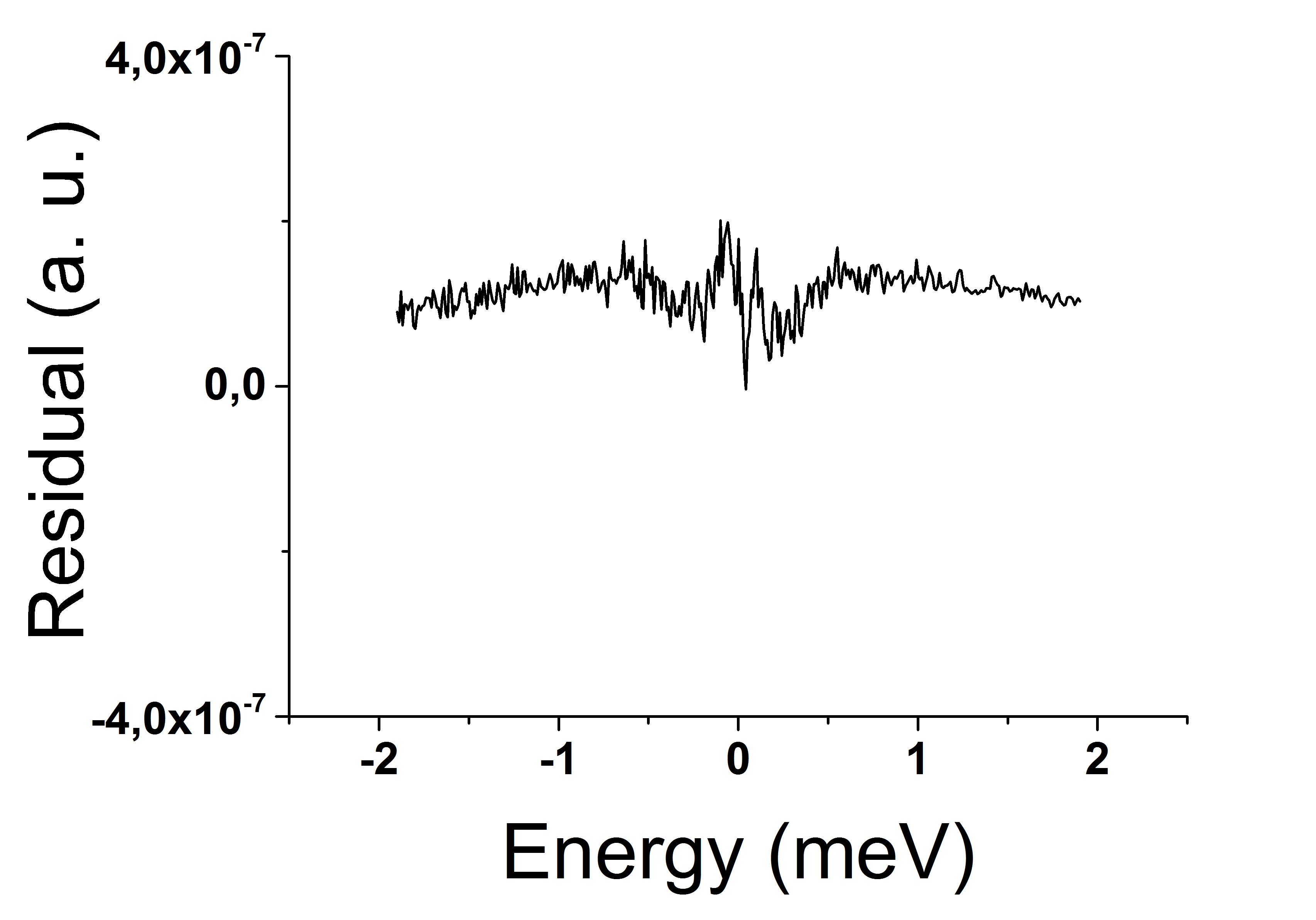 | 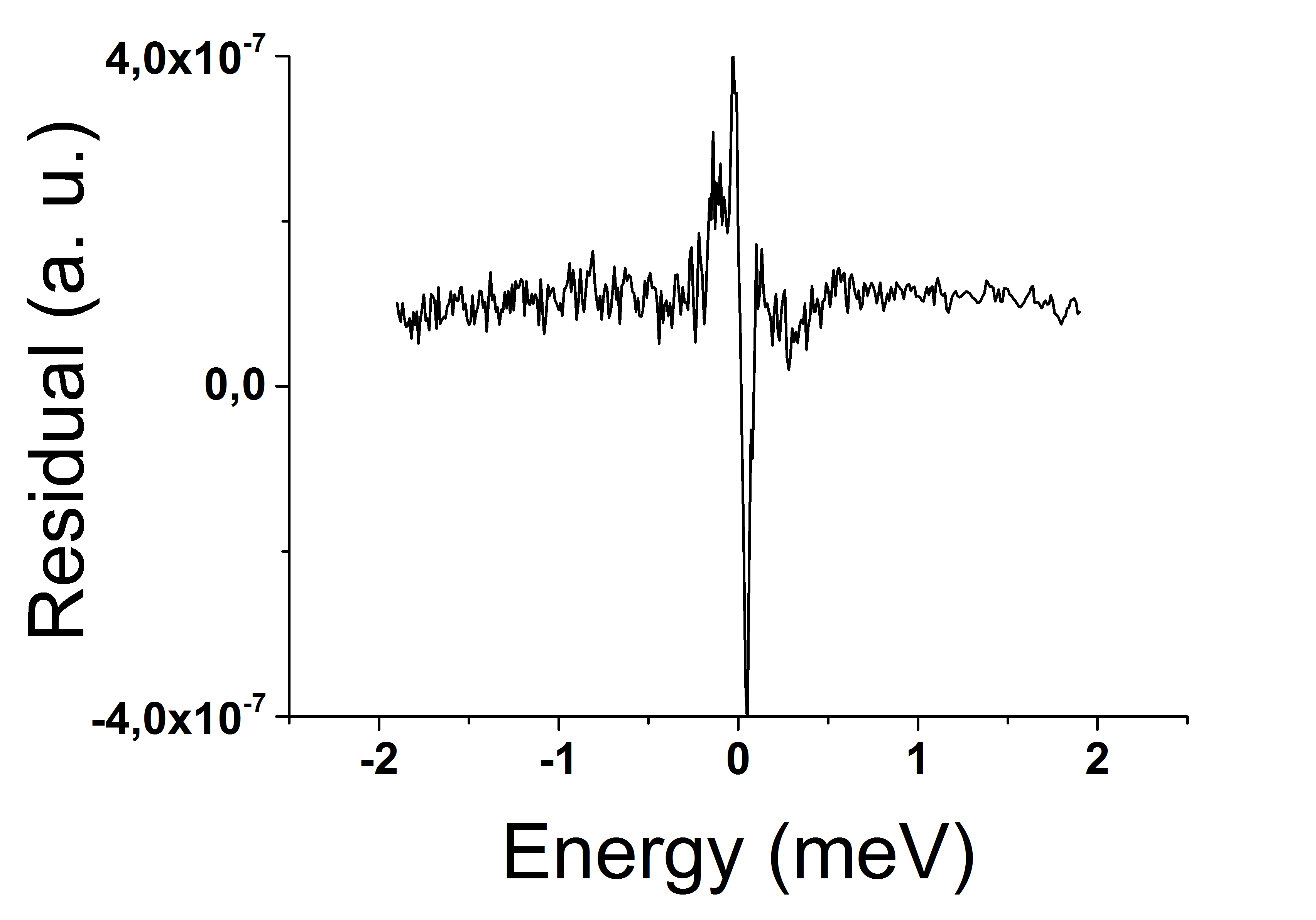 |
| 1.25 | 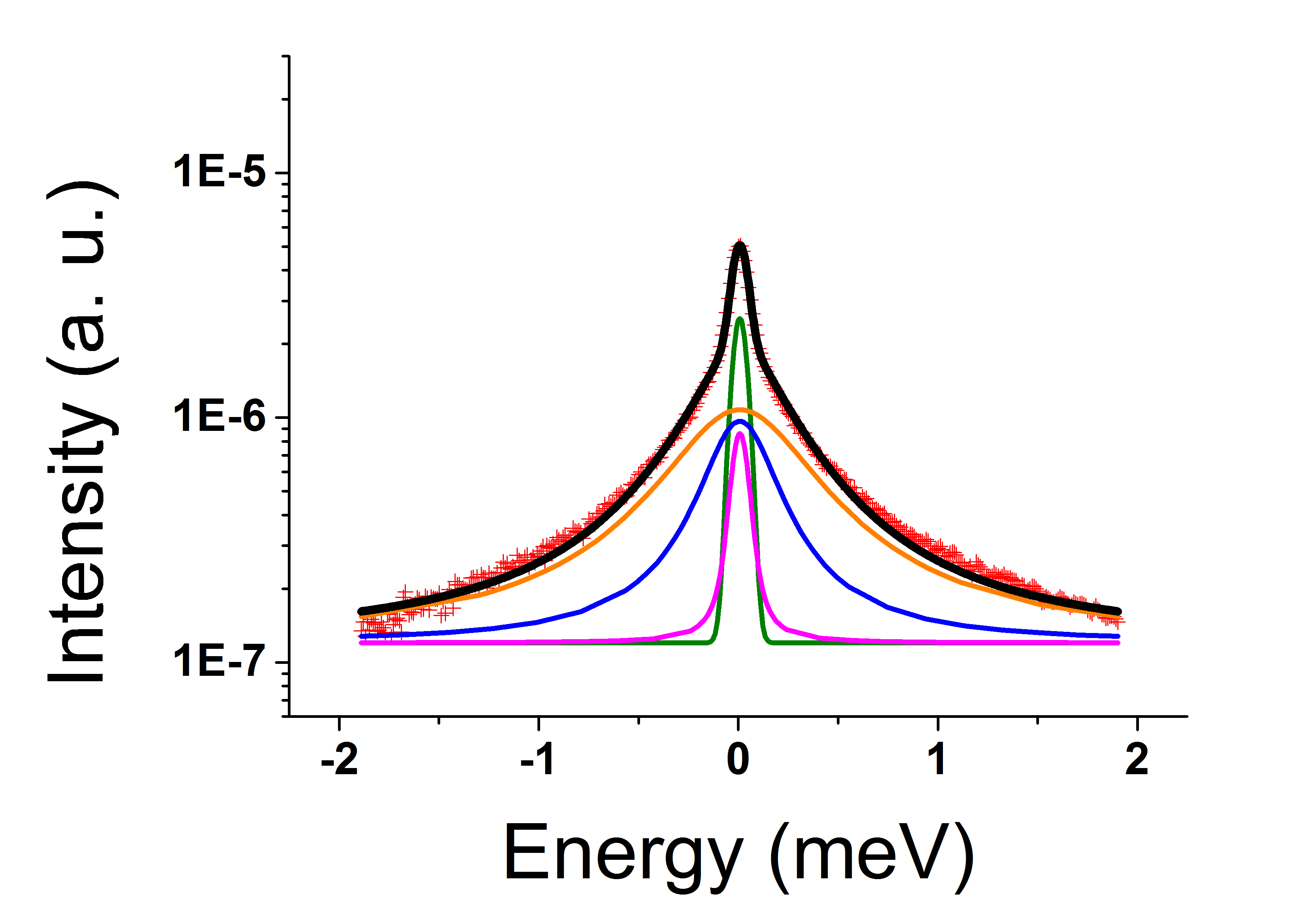 | 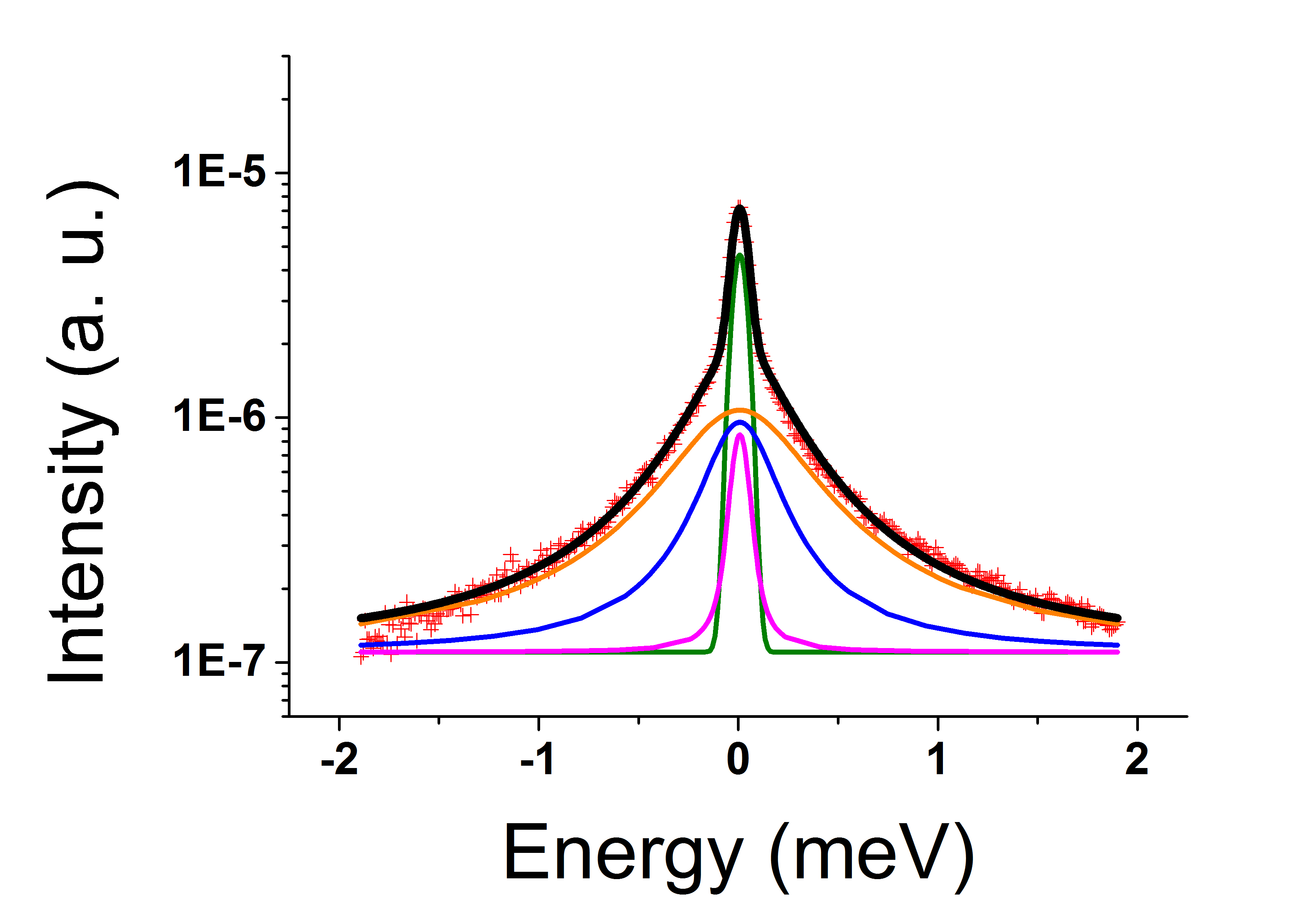 |
|  | 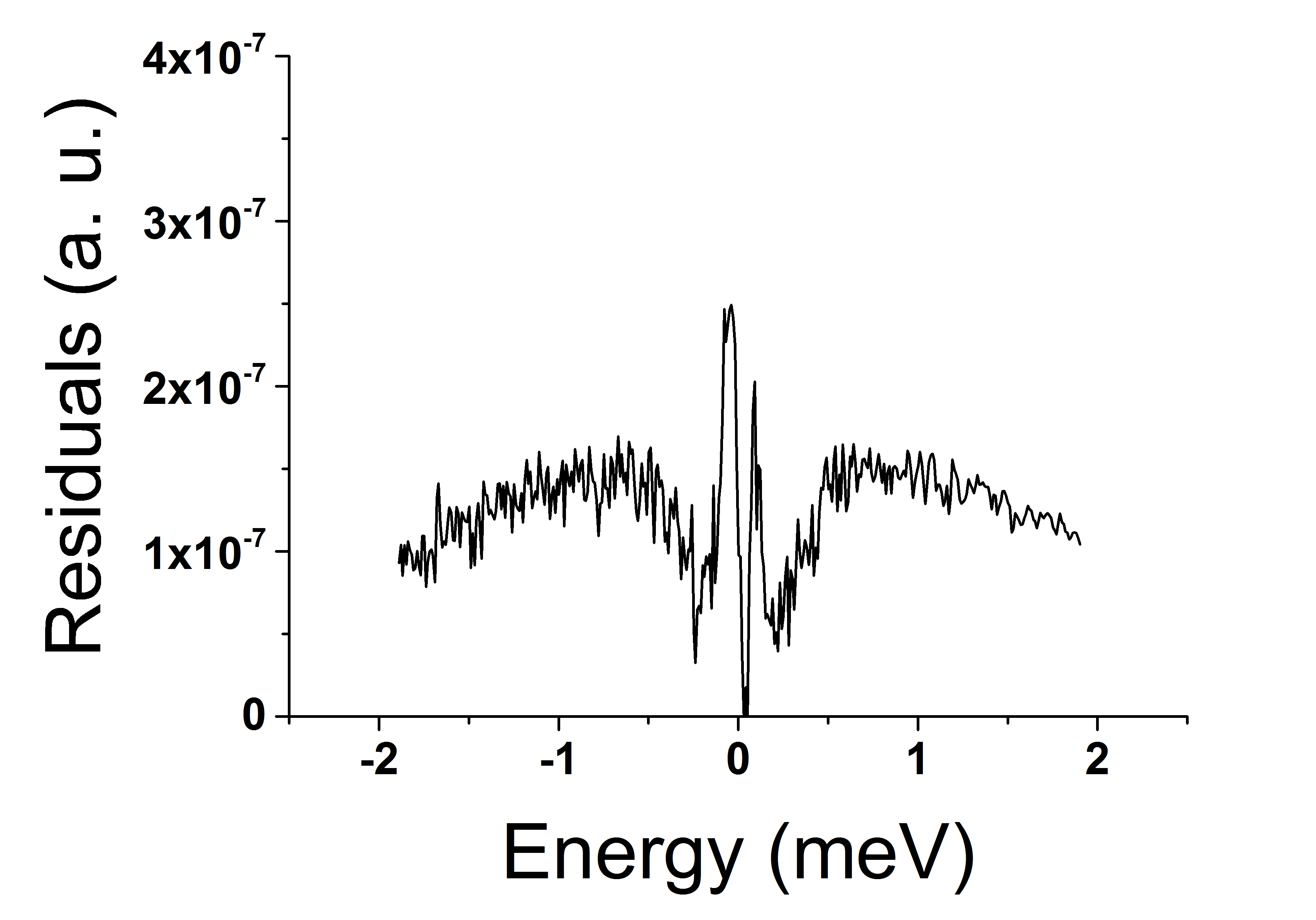 | 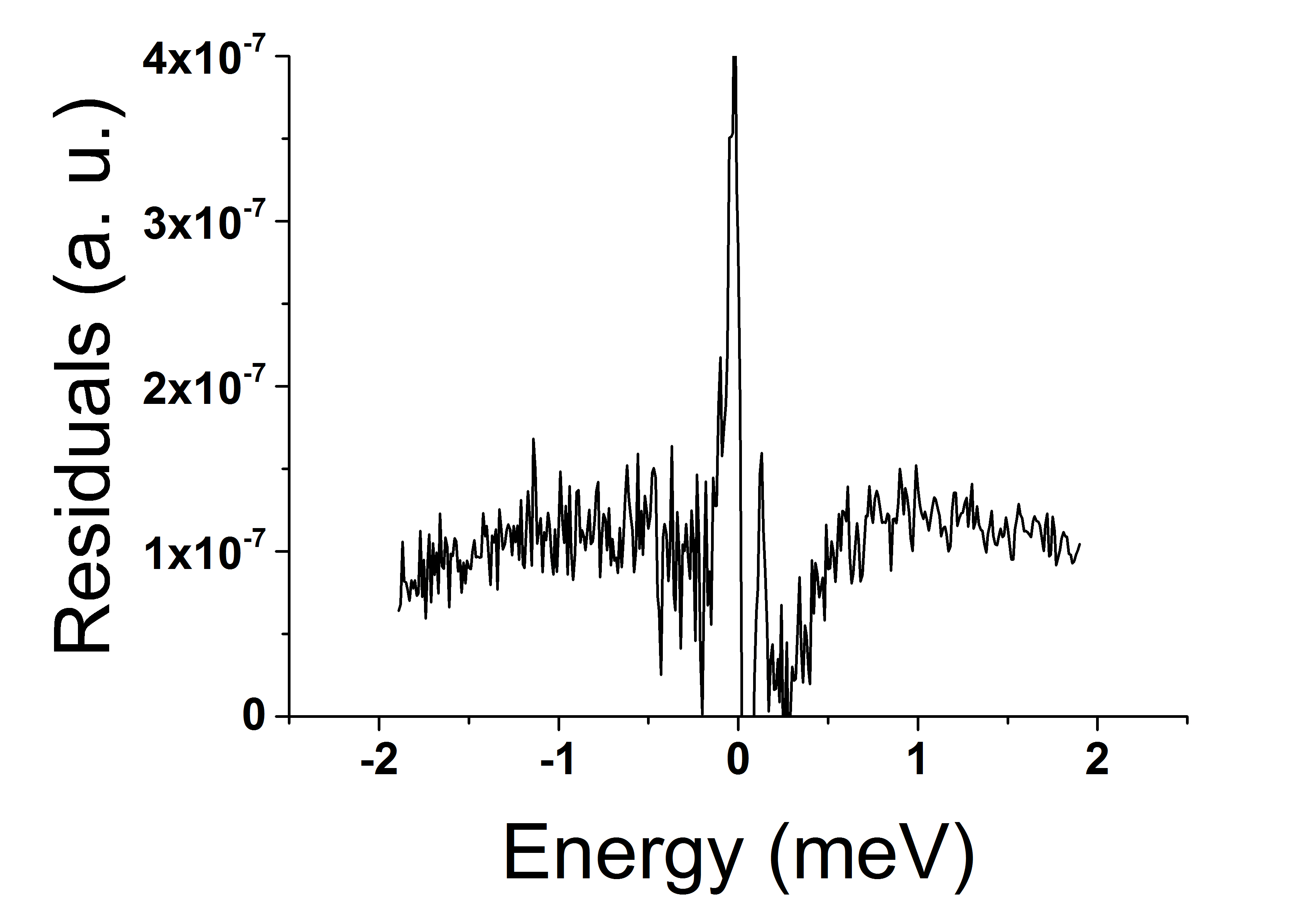 |
| 1.42 | 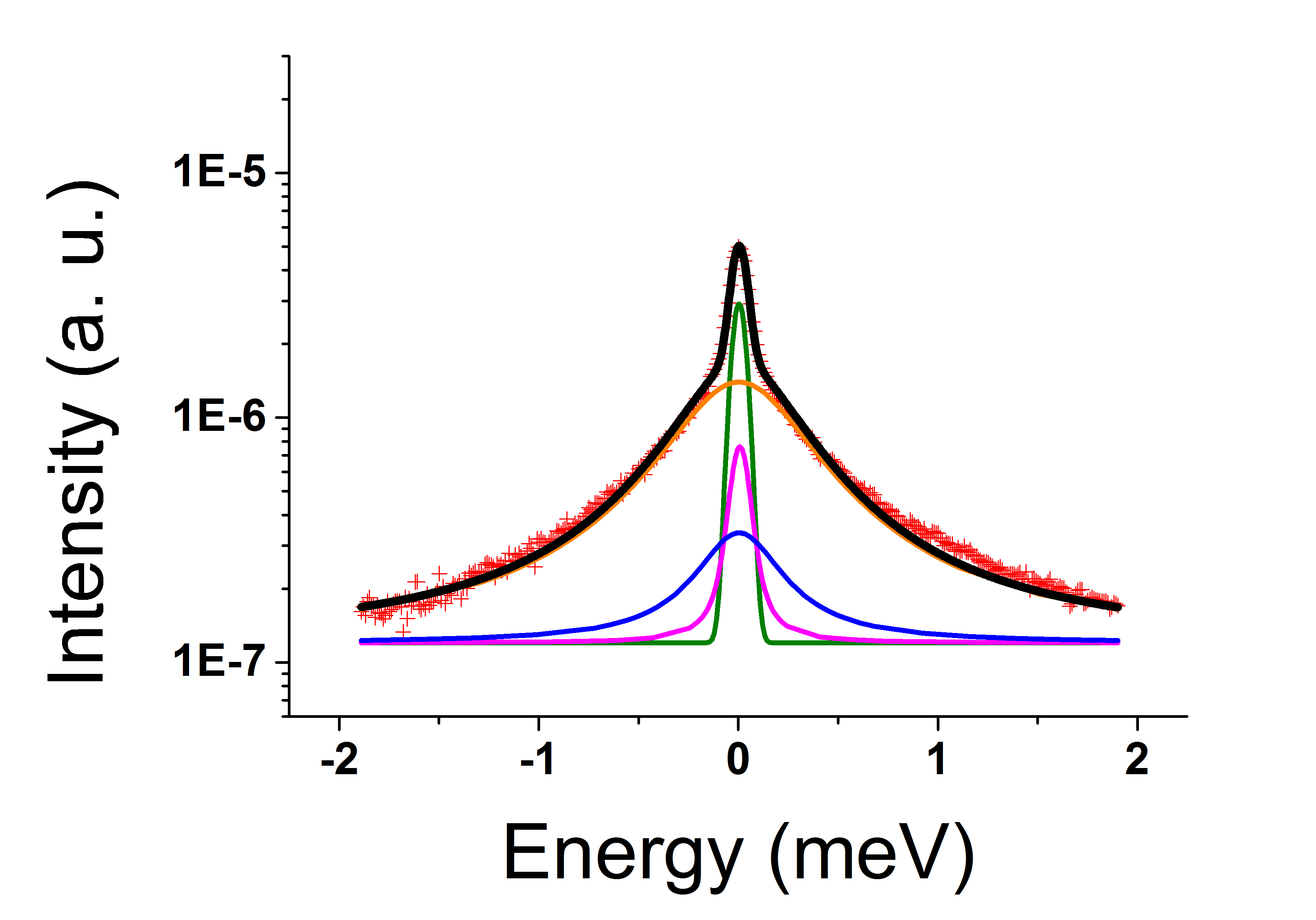 | 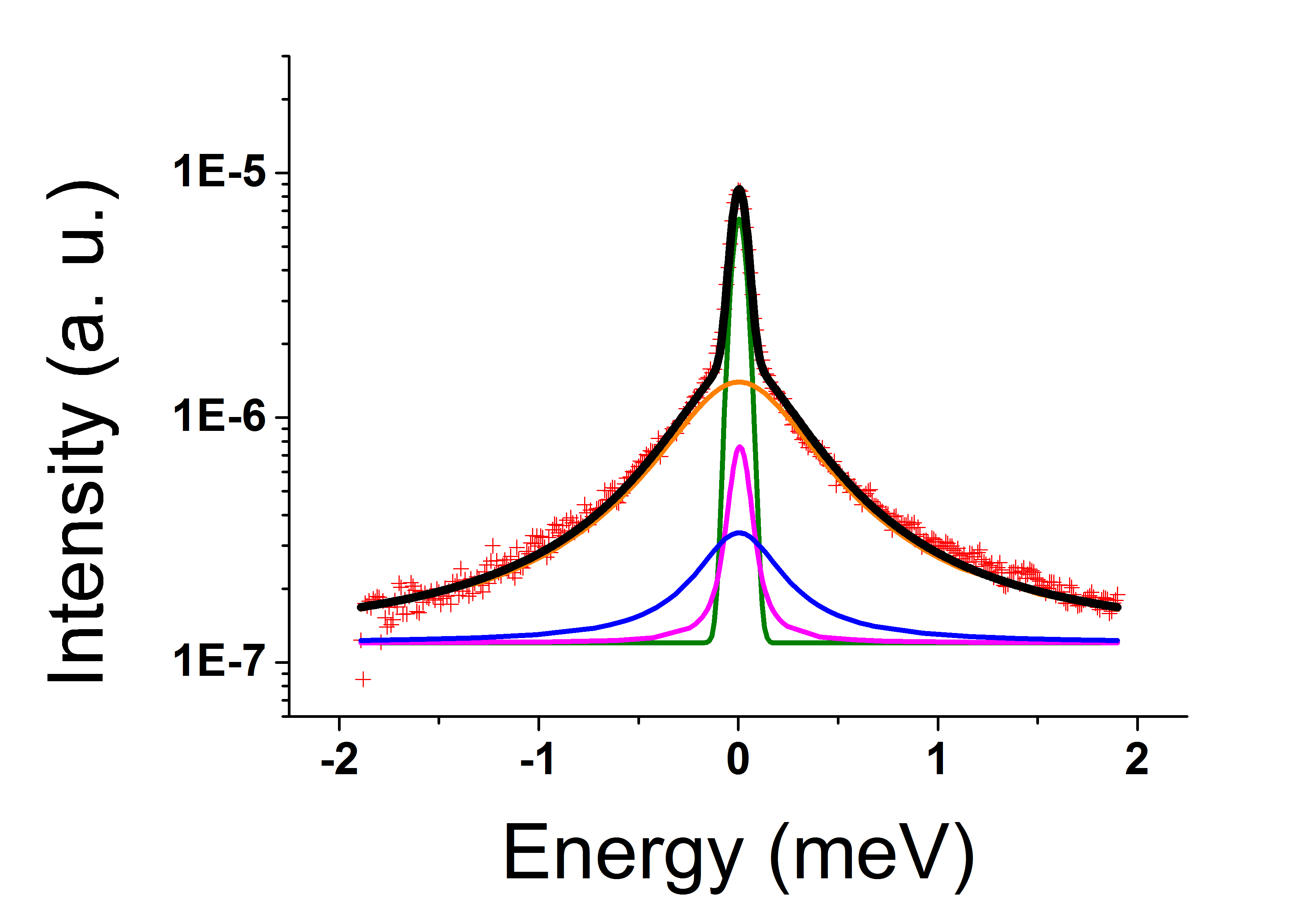 |
|  | 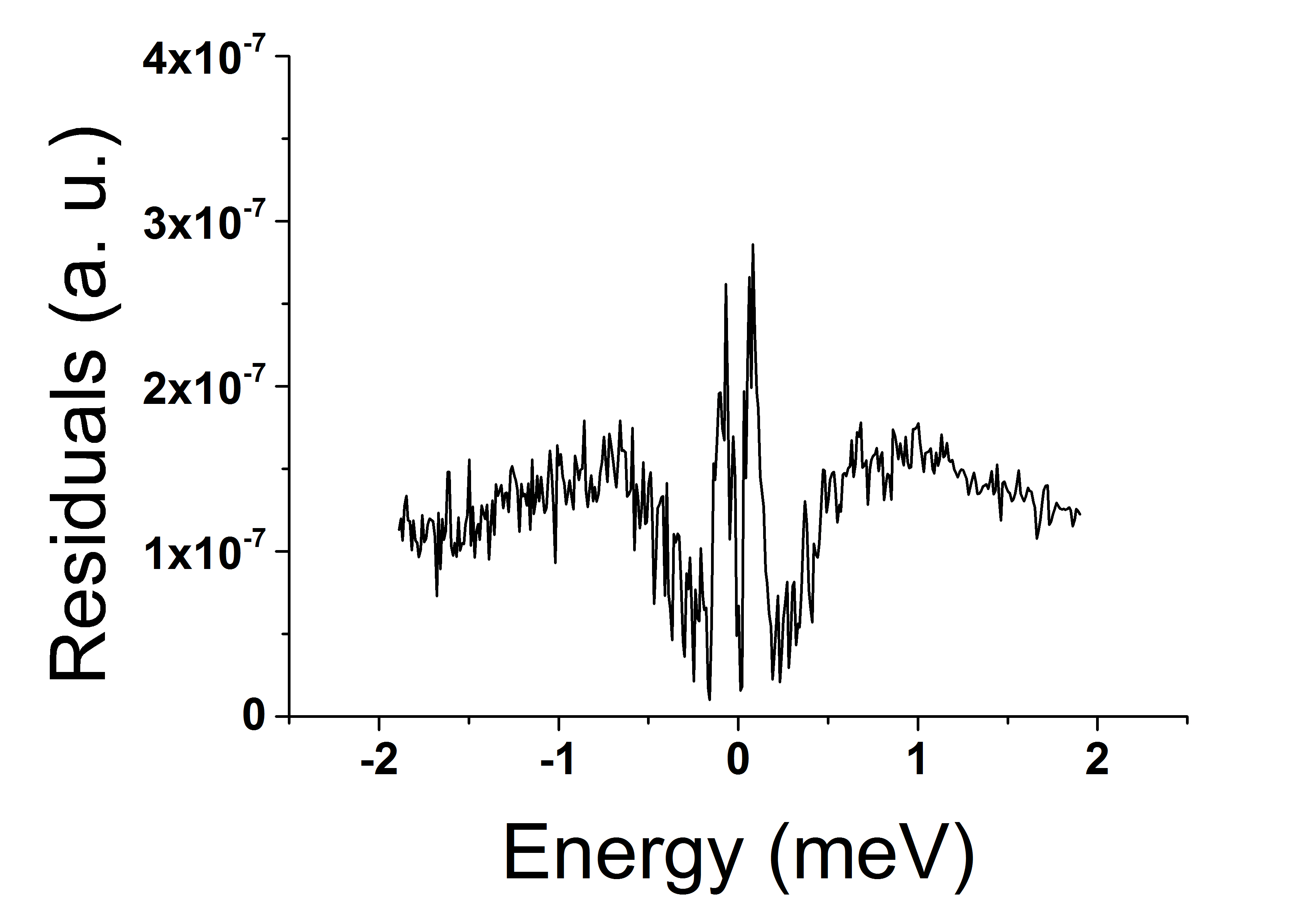 | 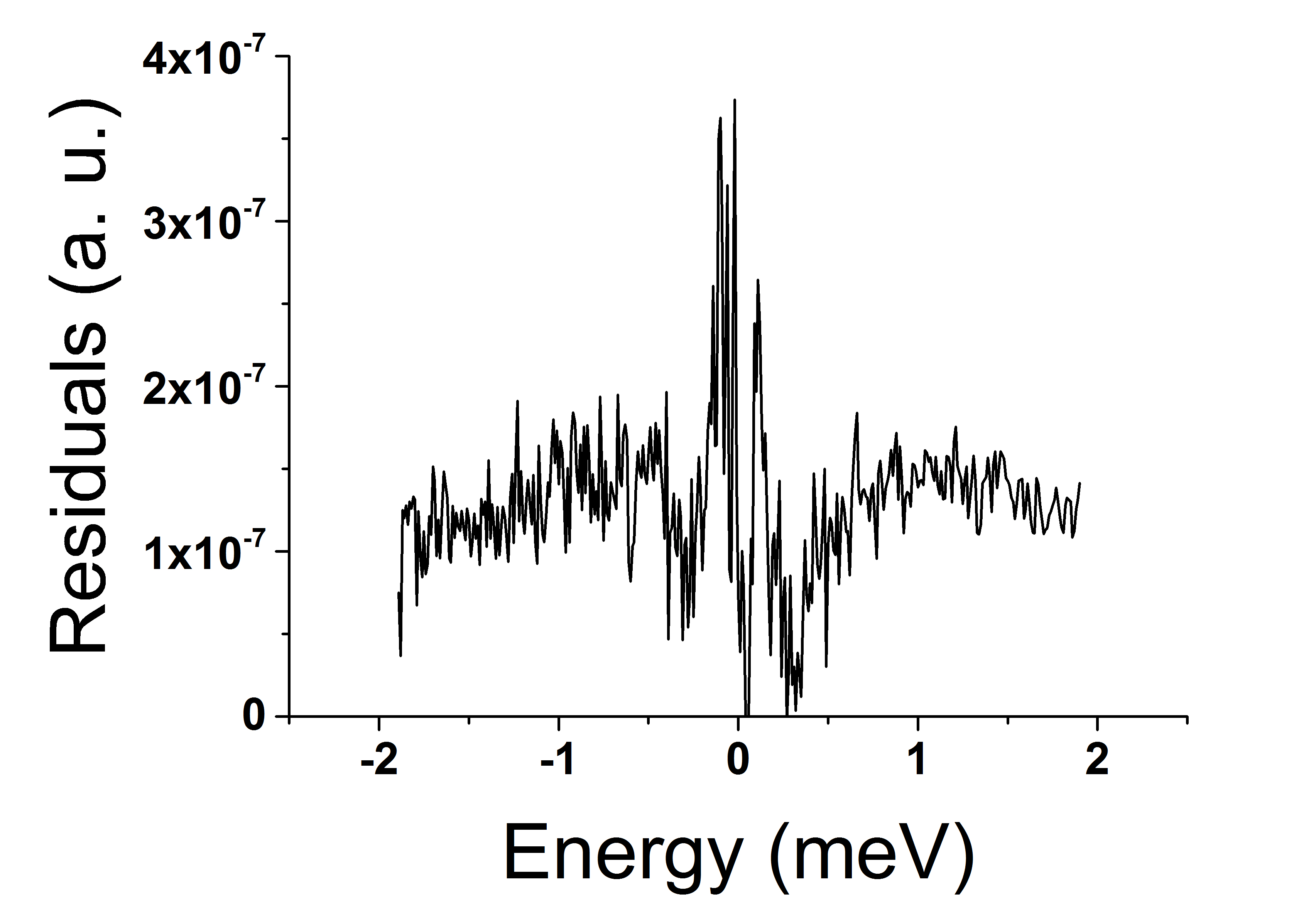 |

**Figure S1.** IN5 and IN6 QENS fits of TG-LDL at 20 bar and 3000 bar including fit residuals. The curves are only shown for TG-LDL as they are virtually identical for N-LDL.

**Table S1.** FWHM values evaluated from data taken on IN5 (a) and IN6 (b) (see also Figure 2b). The estimated error is ± 2 on the last given digit.

(a)

| Q^2^ (Å^-2^) | Γ_small_ (meV) | Γ_intermediate_ (meV) | Γ_big_ (meV) |
| --- | --- | --- | --- |
| 0.07 | 0.001 | 0.025 | 0.72 |
| 0.11 | 0.003 | 0.037 | 0.72 |
| 0.17 | 0.006 | 0.046 | 0.72 |
| 0.23 | 0.008 | 0.062 | 0.72 |
| 0.31 | 0.011 | 0.084 | 0.72 |
| 0.38 | 0.012 | 0.105 | 0.72 |
| 0.47 | 0.017 | 0.128 | 0.72 |
| 0.56 | 0.020 | 0.145 | 0.72 |
| 0.65 | 0.023 | 0.165 | 0.72 |
| 0.74 | 0.026 | 0.185 | 0.72 |
| 0.83 | 0.029 | 0.208 | 0.72 |
| 0.92 | 0.033 | 0.231 | 0.72 |
| 1.02 | 0.038 | 0.252 | 0.72 |

(b)

| Q^2^ (Å^-2^) | Γ_small_ (meV) | Γ_intermediate_ (meV) | Γ_big_ (meV) |
| --- | --- | --- | --- |
| 0.14 | 0.0048 | 0.034 | 0.72 |
| 0.30 | 0.0106 | 0.080 | 0.72 |
| 0.53 | 0.0187 | 0.138 | 0.72 |
| 0.81 | 0.0285 | 0.200 | 0.72 |
| 1.14 | 0.0400 | 0.275 | 0.72 |
| 1.55 | 0.0542 | 0.355 | 0.72 |
| 2.02 | 0.0709 | 0.430 | 0.72 |


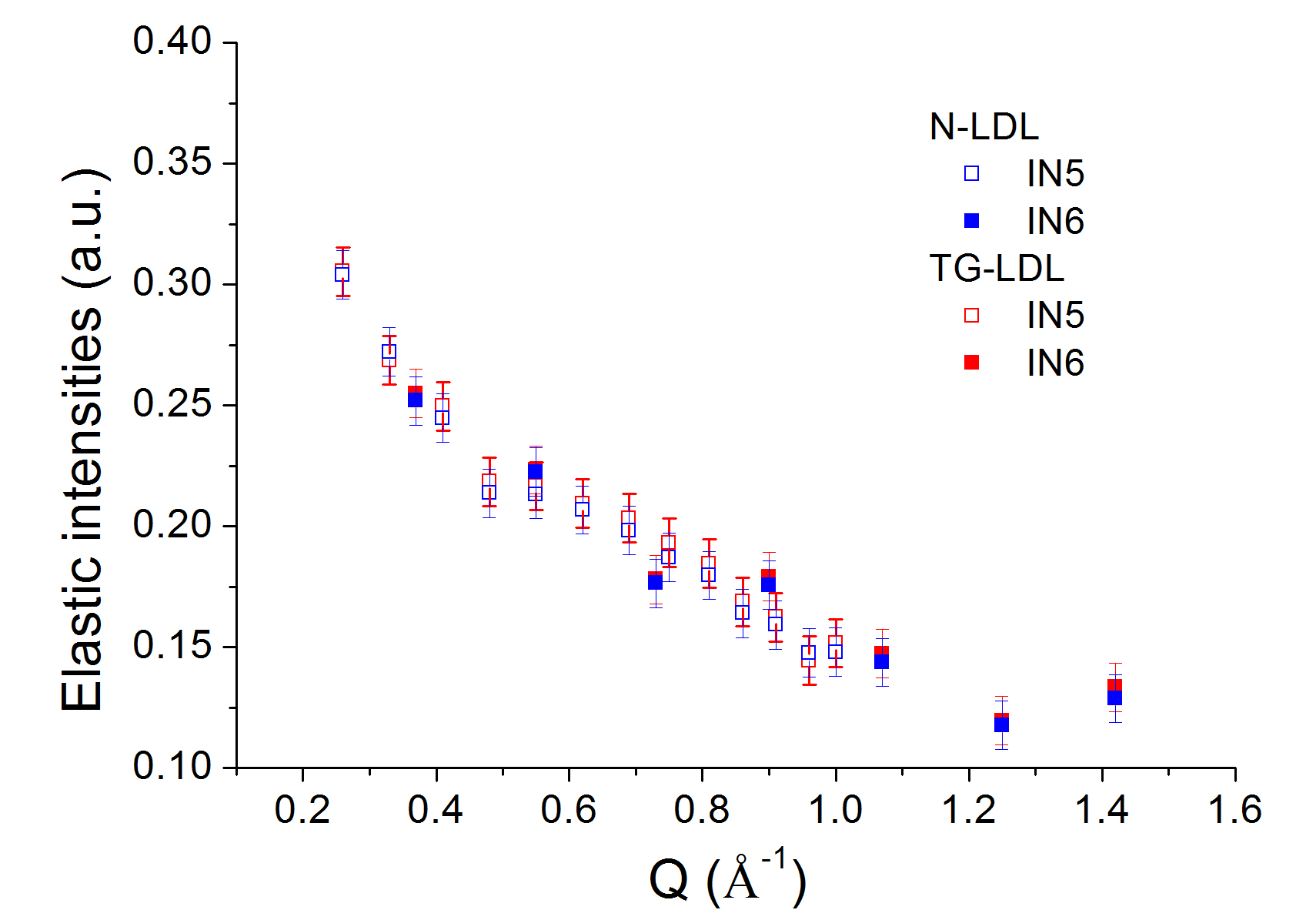


**Figure S2.** Elastic intensities extracted from QENS data as function of Q: N-LDL (blue) and TG-LDL (red) at 20 bar. All data were measured at 310 K on IN5 and IN6.

**Table S2.** MSD values evaluated from data taken on IN13 (see Figure 4a).

|  | MSD (Å^2^) | Error (Å^2^) |
| --- | --- | --- |
| N-LDL 20 bar | 2.83 | 0.21 |
| N-LDL 3000 bar | 3.10 | 0.26 |
| TG-LDL 20 bar | 2.26 | 0.11 |
| TG-LDL 3000 bar | 1.17 | 0.05 |

**Table S3.** Elastic intensities evaluated from QENS data taken on IN5 (a) and IN6 (b) (see also Figure S2) at 20 bar and on IN5 (c) and IN6 (d) (see also Figure 4b) at 3000 bar. The estimated error is 5%.

(a)

| Q (Å^-1^) | N-LDL | TG-LDL |
| --- | --- | --- |
| 0.26 | 0.30388 | 0.30539 |
| 0.33 | 0.27211 | 0.26853 |
| 0.41 | 0.2447 | 0.24964 |
| 0.48 | 0.21363 | 0.21842 |
| 0.55 | 0.21308 | 0.21666 |
| 0.62 | 0.20669 | 0.20942 |
| 0.69 | 0.19823 | 0.20328 |
| 0.75 | 0.18705 | 0.19312 |
| 0.81 | 0.17969 | 0.18444 |
| 0.86 | 0.16403 | 0.16868 |
| 0.91 | 0.1592 | 0.16234 |
| 0.96 | 0.14766 | 0.14444 |
| 1.00 | 0.14782 | 0.15161 |

(b)

| Q (Å^-1^) | N-LDL | TG-LDL |
| --- | --- | --- |
| 0.37 | 0.25182 | 0.25498 |
| 0.55 | 0.22249 | 0.22328 |
| 0.73 | 0.17632 | 0.17801 |
| 0.90 | 0.17564 | 0.17914 |
| 1.07 | 0.14371 | 0.14724 |
| 1.25 | 0.11767 | 0.11951 |
| 1.42 | 0.12873 | 0.13339 |

(c)

| Q (Å^-1^) | N-LDL | TG-LDL |
| --- | --- | --- |
| 0.26 | 0.31 | 0.31 |
| 0.33 | 0.27 | 0.30 |
| 0.41 | 0.25 | 0.27 |
| 0.48 | 0.22 | 0.26 |
| 0.55 | 0.22 | 0.26 |
| 0.62 | 0.21 | 0.26 |
| 0.69 | 0.20 | 0.27 |
| 0.75 | 0.19 | 0.26 |
| 0.81 | 0.18 | 0.27 |
| 0.86 | 0.17 | 0.26 |
| 0.91 | 0.16 | 0.25 |
| 0.96 | 0.14 | 0.23 |
| 1.00 | 0.15 | 0.23 |

(d)

| Q (Å^-1^) | N-LDL | TG-LDL |
| --- | --- | --- |
| 0.37 | 0.26 | 0.29 |
| 0.55 | 0.22 | 0.26 |
| 0.73 | 0.18 | 0.27 |
| 0.90 | 0.18 | 0.26 |
| 1.07 | 0.15 | 0.23 |
| 1.25 | 0.12 | 0.20 |
| 1.42 | 0.13 | 0.23 |

**Table S4. Fitted radii of a triaxial ellipsoid extracted from the SANS curves. The error of the radii was estimated to ± 1 Å. For technical reasons we could not measure TG-LDL at 20 bar.**

| N-LDL | | | | | | | |
| --- | --- | --- | --- | --- | --- | --- | --- |
|  | 313 K | | | | | | |
| Pressure (bar) | R_3_ (Å) | | R_2_ (Å) | | R_1_ (Å) | | Rg (Å) |
| 20 | 78 | | 101 | | 123 | | 79 |
| 300 | 77 | | 101 | | 123 | | 79 |
| 600 | 77 | | 101 | | 123 | | 79 |
| 1200 | 75 | | 101 | | 121 | | 78 |
| 2000 | 74 | | 100 | | 121 | | 78 |
| 3000 | 72 | | 99 | | 120 | | 77 |
| TG-LDL | | | | | | | |
|  | 313 K | | | | | | |
| Pressure (bar) | R_3_ (Å) | R_2_ (Å) | | R_1_ (Å) | | Rg (Å) | |
| 20 | N.D.* | N.D.* | | N.D.* | | N.D.* | |
| 600 | 82 | 99 | | 117 | | 78 | |
| 1200 | 78 | 99 | | 118 | | 77 | |
| 2000 | 79 | 98 | | 118 | | 77 | |
| 3000 | 77 | 98 | | 120 | | 76 | |

* not determined


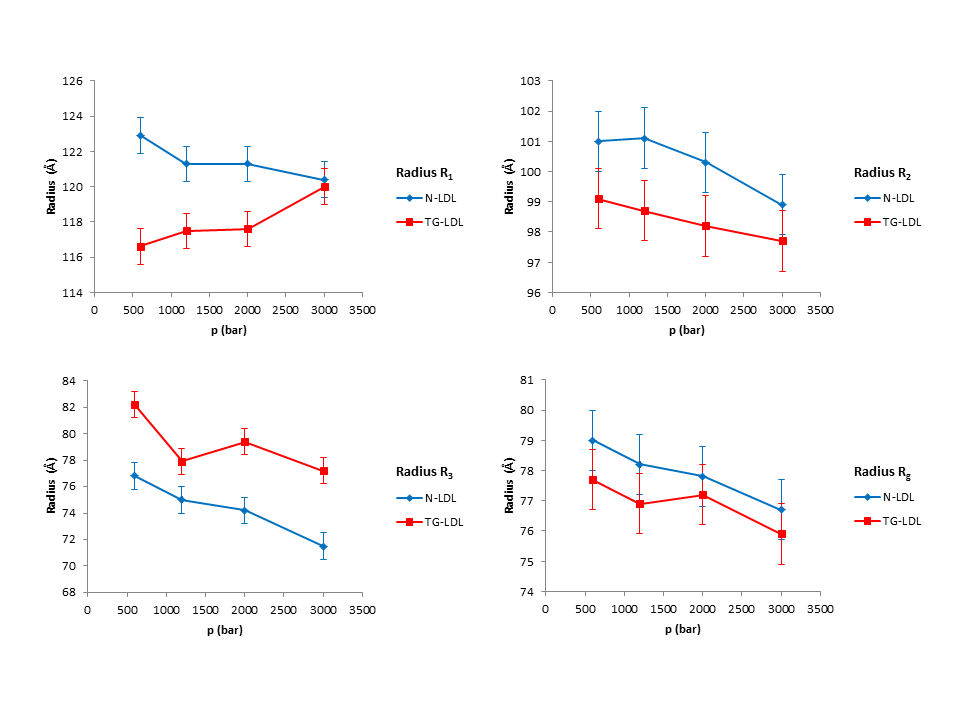


**Figure S3.** Separate graphs for the changes of the individual radii (R1, R2 and R3) derived by fitting the SANS curves with an ellipsoidal model as well as the R_g_ are shown.

**Table S5: Chemical compositions (wt%) of normolipidemic N-LDL and triglyceride-rich TG-LDL. Values are means ± SD of duplicate or triplicate determinations of each component.**

| **Component** | **QENS** | | **EINS** | | **SANS** | |
| --- | --- | --- | --- | --- | --- | --- |
|  | **N-LDL** | **TG-LDL** | **N-LDL** | **TG-LDL** | **N-LDL** | **TG-LDL** |
| Protein | 21.1 ± 0.8 | 19.3 ± 0.8 | 20.5 ± 3.2 | 16.6 ± 0.2 | 19.7 ± 0.2 | 19.2 ± 0.8 |
| Phospholipids | 22.0 ± 0.1 | 19.9 ± 0.4 | 20.9 ± 2.3 | 21.9 ± 1.7 | 22.6 ± 1.1 | 20.9 ± 2.0 |
| Unesterified cholesterol | 8.3 ± 0.1 | 6.1 ± 0.3 | 9.3 ± 0.7 | 7.0 ± 0.8 | 7.4 ± 0.3 | 5.9 ± 0.6 |
| Cholesteryl esters | 43.9 ± 1.3 | 43.6 ± 0.8 | 41.9 ± 1.1 | 44.4 ± 0.7 | 44.0 ± 0.5 | 43.0 ± 1.2 |
| Triglycerides | 4.7 ± 0.1 | 11.2 ± 0.1 | 7.4 ± 0.5 | 10.1 ± 0.8 | 6.4 ± 0.1 | 11.0 ± 0.2 |

**Table S6:** Protein concentrations (mg/mL) of the measured LDL samples. Values are means ± SD of duplicate or triplicate determinations. All samples were in the buffer system 10 mM NaPi (1.44 g/L Na_2_HPO_4_∙2H_2_O, 0.26 g/L KH_2_PO_4_), 0.1 % EDTA, pH 7.4 in D_2_O. A remaining amount of approximately 5-10 % of H_2_O was in the buffer system due to the isolation procedure from a natural source.

| **Method** | **N-LDL** | **TG-LDL** |
| --- | --- | --- |
| QENS | 17.4 ± 1.3 | 23.0 ± 1.8 |
| EINS | 12.2 ± 0.4 | 24.7 ± 1.7 |
| SANS | 2.9 ± 0.13 | 2.9 ± 0.19 |

**Table S7:** Instrumental characteristics of the spectrometers IN5, IN6 and IN13 at the ILL.

| Spectrometer | IN5 | IN6 | IN13 |
| --- | --- | --- | --- |
| Wavelength [Å] | 10 | 5.1 | 2.23 |
| Accessible Q-range [Å^-1^] | 0.3 – 1.0 | 0.4 - 2.0 | 0.2 - 4.9 |
| Length scale [Å] | 6 - 21 | 3 – 16 | 1 - 30 |
| Energy resolution [μeV] | 10 | 75 | 10 |
| Time scale [ps] | 100 | 15 | 100 |


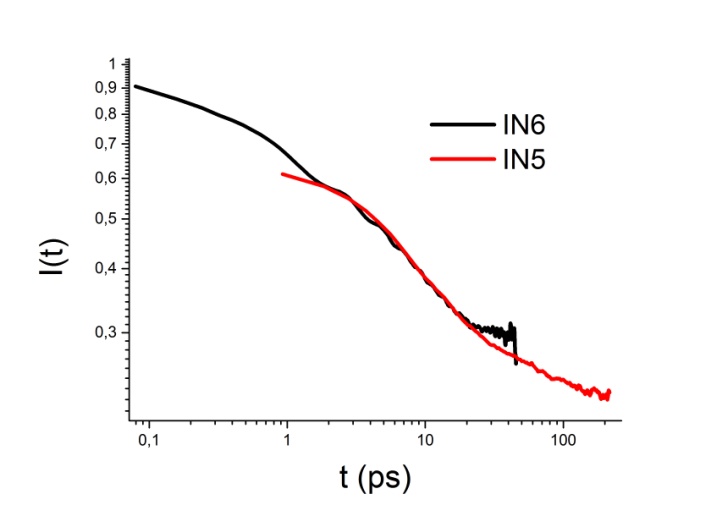


**Figure S4.** Indirect Fourier Transformation for Q = 0.73 Å^-1^, processed with the program Dave^S1^, for the QENS spectra of N-LDL, measured at 3000 bar on IN5 (red line) and IN6 (black line) spectrometers.


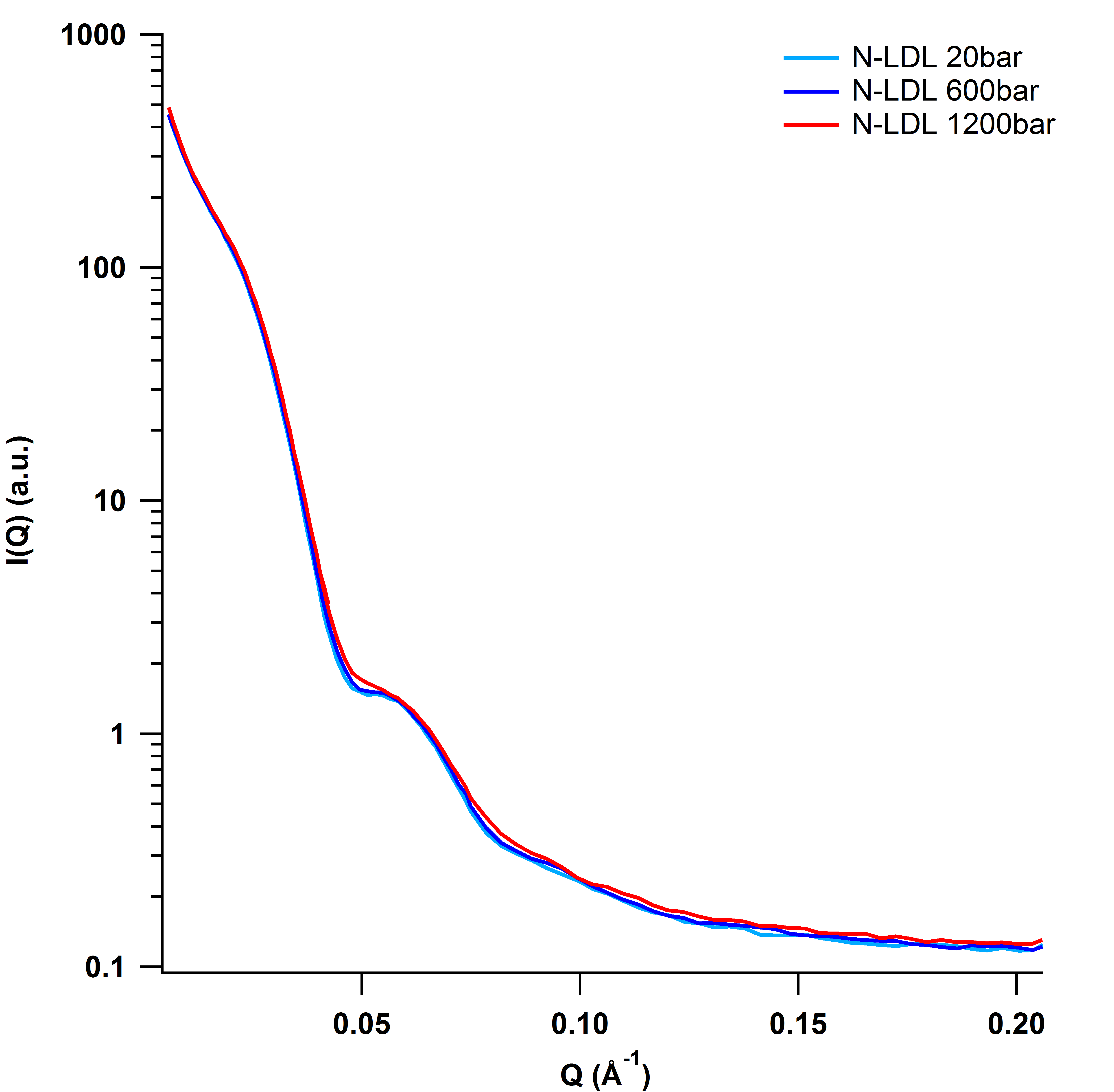


**Figure S5.** The measured SANS curves for N-LDL at pressure points 20 bar (light blue), 600 bar (dark blue) and 1200 bar (red).

**Reference**

S1. Azuah, R.T, Kneller, L.R., Qiu, Y., Tregenna-Piggott, P.L.W., Brown, C.M., Copley, J.R.D., and Dimeo, R.M. [DAVE: A comprehensive software suite for the reduction, visualization, and analysis of low energy neutron spectroscopic data](http://dx.doi.org/10.6028/jres.114.025), *J. Res. Natl. Inst. Stan. Technol.* **114**, 341 (2009).
